# Secondary Metabolites from the Endophytic Fungus *Xylaria* sp. hg1009

**DOI:** 10.1007/s13659-018-0158-x

**Published:** 2018-03-20

**Authors:** Rong Chen, Jian-Wei Tang, Xing-Ren Li, Miao Liu, Wen-Ping Ding, Yuan-Fei Zhou, Wei-Guang Wang, Xue Du, Han-Dong Sun, Pema-Tenzin Puno

**Affiliations:** 1 School of Chemical Science and Technology, Yunnan University, Kunming, 650091 People’s Republic of China; 2 State Key Laboratory of Phytochemistry and Plant Resources in West China, Kunming Institute of Botany, Chinese Academy of Sciences, Kunming, 650201 People’s Republic of China; 3 University of Chinese Academy of Sciences, Beijing, 100049 People’s Republic of China

**Keywords:** Endophytic fungus, *Xylaria* sp., Secondary metabolites, Cytotoxicity

## Abstract

A detailed chemical investigation of the secondary metabolites produced by the endophytic fungus *Xylaria* sp. isolated from the stems of *Isodon sculponeatus* afforded six new compounds, xylariahgins A–F (**1**–**6**), two new natural products (**7** and **8**), along with two known compounds (**9** and **10**) (Fig. 1). The structures of all compounds were unambiguously established by analyzing their spectroscopic data or referring to pertinent literature. Compounds **1**–**8** were tested for their cytotoxic activity against five human tumor cell lines.

**Electronic supplementary material:**

The online version of this article (10.1007/s13659-018-0158-x) contains supplementary material, which is available to authorized users.

## Introduction

Endophytic fungi are microorganisms, parasitizing plants without causing any obvious disease at any specific moment of their colonizing period [1]. Although interactions between endophytes with their hosts are not fully understood in most cases, many endophytes produce bioactive secondary metabolites that may protect hosts from herbivores, plant pathogens, and abiotic stressors [2, 3]. The bioactive secondary metabolites produced by endophytes may be useful in medicine, industry and agriculture [4–8]. In addition, plants which possess significant biological activity have the potential of harbouring endophytes with great biodiversity [9]. The genus *Isodon* is an abundant source of diterpenoids with diverse chemical structures, of which some ones exhibit antitumor and anti-inflammatory activities [10–12]. Therefore, we assumed that the secondary metabolites produced by endophytic fungi in *Isodon* species could be functional in biology and structural diversity. Our previous investigation had already testified that some secondary metabolites secreted by endophytic fungus from *I. eriocalyx* var. *laxiflora* possessed interesting structure and diverse bioactivities, such as penicilfuranone A isolated from *Penicillium* sp. sh18 [13], phomopchalasins A and B isolated from *Phomopsis* sp. shj2 [14] and phomopsiketones A–C isolated from *Phomopsis* sp. sh917 [15]. Accordingly, we have extended our attention to endophytic fungus in *Isodon* species. In this research, we have conducted our research with the fungus *Xylaria* sp., isolated from the stems of *I*. *sculponeatus*. Fungi in the genus *Xylaria* have been a rich source of compounds with intriguing structure, including pestalotin 4′-*O*-methyl-*β*-mannopyranoside and 3*S*,4*R*-(+)-4-hydroxymellein from *Xylaria feejeensis* [16], nigriterpenes A–F from *X. nigripes* YMJ653 [17], cytochalasin Z_27_ and cytochalasin Z_28_ from *Xylaria* sp. XC-16 [18].

**Fig. 1:**
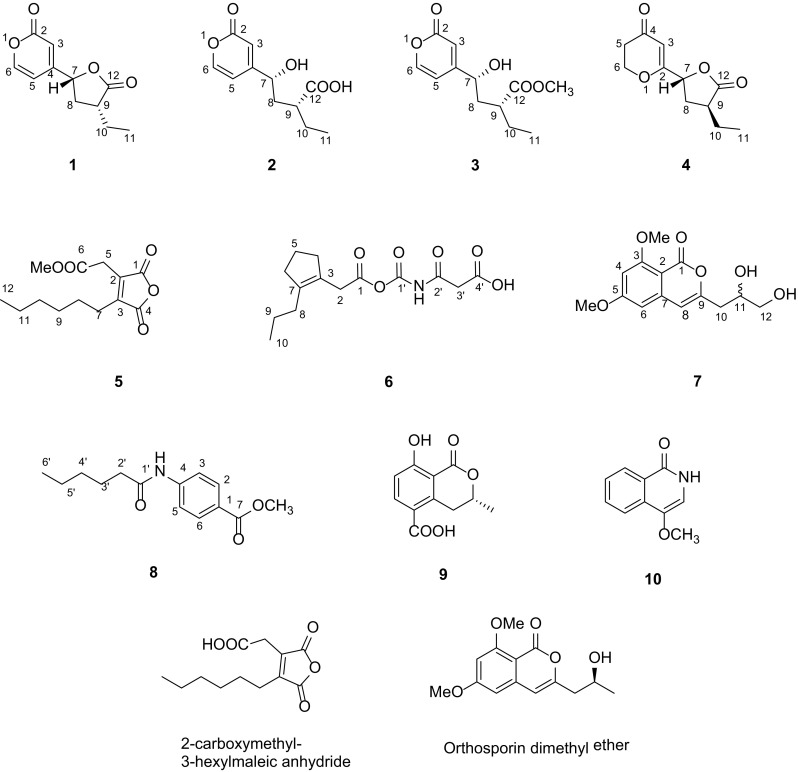
Structures of compounds **1**–**10** and orthosporin dimethyl ether

**Fig. 2:**
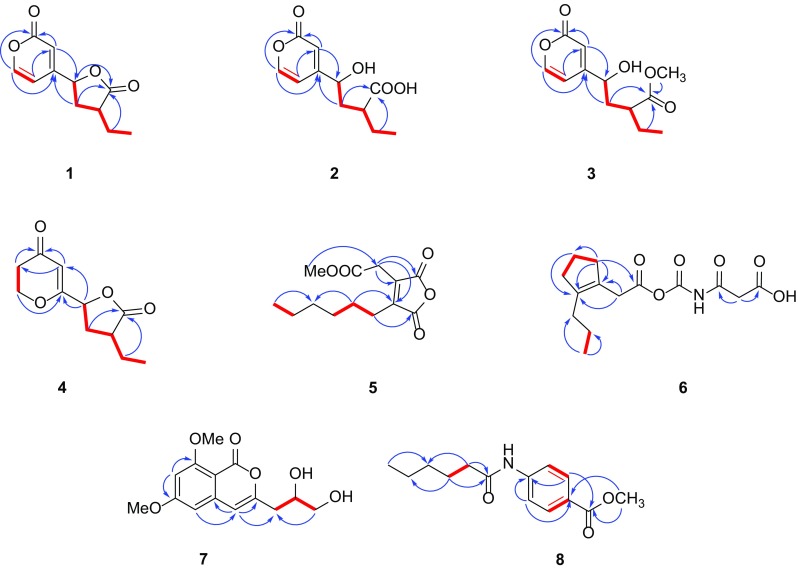
Key HMBC (blue arrows H → C), ^1^H–^1^H COSY (red lines) correlations of **1**–**8**

Through OSMAC strategy analysis [19], Rice and MEA media were selected as the larger fermentation media for subsequent study. As a result, ten compounds, including six new ones, named xylariahgins A–F (**1**–**6**), two new natural products, 3-(2,3-dihydroxypropyl)-6,8-dimethoxyisocoumarin and methyl 4-(hexanoylamino) benzoate (**7** and **8)**, and two known ones, (−)-5-carboxylmellein and 4-methoxyisoquinolin-1(2*H*)-one (**9** and **10**), were discovered from two culture extract. The isolation, structure elucidation and bioactivity of these compounds, are herein described (Figs. 1, 2).

## Results and Discussion

**Fig. 3:**
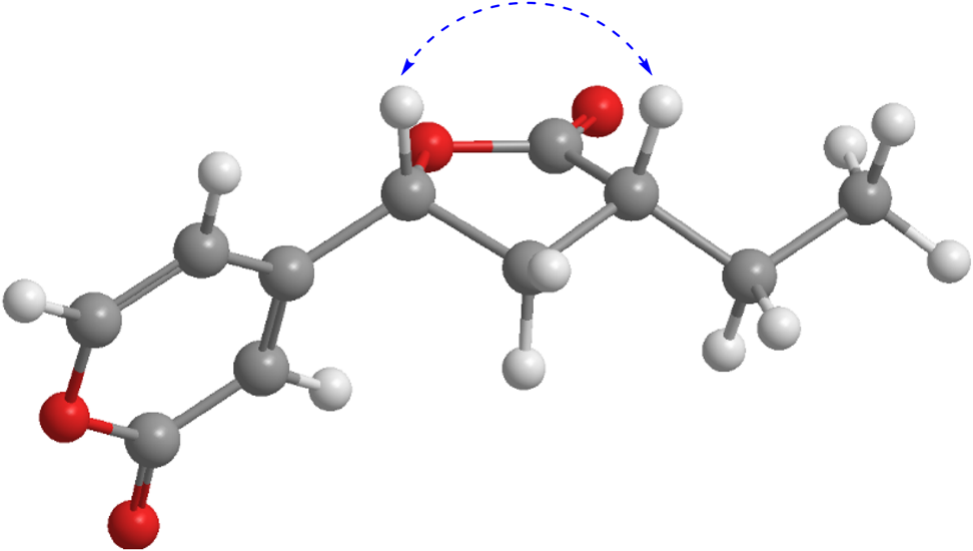
ROESY correlation (blue dashed double-headed arrow) of **1**

On the basis of HRESIMS ([M+Na]^+^ 231.0624, calcd for 231.0628) and ^13^C NMR data, xylariahgin A (**1)** has the molecular formula C_11_H_12_O_4_, indicating six degrees of unsaturation. Its IR absorptions at 1781 and 1663 cm^−1^ implied the existence of lactone and C=C groups. The ^1^H, ^13^C NMR and DEPT spectra revealed the presence of one methyl group, two methylenes, five methines including three olefinic methines, three quarternary carbons (two carbonyls, one olefinic group) (Table 1). The ^1^H–^1^H COSY spectrum of **1** exhibited the correlations of H_3_-11/H_2_-10/H-9/H_2_-8/H-7. While its HMBC spectrum showed the correlations from H_b_-10 (*δ*_H_ 1.53, m) to C-8 and C-12, H_b_-8 (*δ*_H_ 2.06, m) to C-10 and C-12, H-7 (*δ*_H_ 5.28, dd, 9.9, 6.1) to C-12. These results indicated that **1** has a furanone moiety. Similarly, the structure of pyrone is supported by the HMBC correlations from H-5 (*δ*_H_ 6.26, dd, 5.9, 2.5) to C-6 and C-3, from H-3 (*δ*_H_ 6.37, d, 2.5) to C-4, C-7 and C-2, from H-6 (*δ*_H_ 8.05, d, 5.9) to C-4, C-2 and C-5 (Fig. 2). Furthermore, the HMBC correlations from H_b_-8 to C-4, from H-3 to C-7 determined the direct connection of the furanone moiety with the pyrone moiety. Additionally, the key ROESY correlation between H-7 and H-9 supported that the H-7 and H-9 were cofacial and were arbitrarily assigned as *β*-oriented (Fig. 3). Therefore, the structure and relative configuration of compound **1** was determined.

**Table 1:** NMR data of **1**–**4** (*δ* in ppm, *J* in Hz)

No	**1** ^a^		**2** ^b^		**3** ^c^		**4** ^b^	
	*δ*_H_	*δ*_C_	*δ*_H_	*δ*_C_	*δ*_H_	*δ*_C_	*δ*_H_	*δ*_C_
1								
2		178.3, s		180.4, s		178.6, s		171.6, s
3	6.37, 1H (d, 2.5)	115.1, d	6.59, 1H (d, 2.4)	112.7, d	6.30, 1H (d, 2.5)	113.5, d	5.61, 1H (s)	103.7, d
4		165.2, s		171.3, s		171.2, s		191.6, s
5	6.26, 1H (dd, 5.9, 2.5)	117.7, d	6.34, 1H (dd, 5.8, 2.4)	116.7, d	6.17, 1H (dd, 5.8, 2.5)	117.4, d	2.60, 2H (m)	36.1, t
6	8.05, 1H (d, 5.9)	156.9, d	7.77, 1H (d, 5.8)	155.6, d	7.97, 1H (d, 5.8)	156.3, d	4.56, 2H (m)	68.7, t
7	5.28, 1H (dd, 9.9, 6.1)	75.4, d	4.56, 1H (dd, 8.6, 4.2)	69.7, d	4.53, 1H (m)	69.1, d	4.80, 1H (dd, 9.5, 6.5)	75.0, d
8	2.82, 1H (m)	32.8, t	2.13, 1H (m)	36.8, t	2.15, 1H (m)	37.7, t	2.62, 1H (m)	32.3, t
	2.06, 1H (m)		1.96, 1H (m)		1.93, 1H (m)		1.96, 1H (m)	
9	2.83, 1H (m)	41.9, d	2.49, 1H (m)	44.3, d	2.55, 1H (m)	44.3, d	2.61, 1H (m)	41.3, d
10	1.86, 1H (m)	23.9, t	1.70, 1H (m)	26.1, t	1.62, 2H (m)	26.1, t	1.95, 1H (m)	23.5, t
	1.53, 1H (m)		1.58, 1H (m)				1.55, 1H (m)	
11	0.99, 3H (t, 7.5)	11.7, q	0.96, 3H (t, 7.4)	11.5, q	0.88, 3H (t, 7.4)	11.7, q	1.01, 3H (t, 7.5)	11.6, q
12		177.8, s		179.9, s		176.3, s		177.2, s
12-OMe					3.60, 3H (s)	51.7, q		

The assignments were based on HSQC, ^1^H-^1^H COSY, and HMBC experiments

^a1^H and ^13^C NMR data were recorded at 500 MHz and 125 MHz in Acetone-*d*_6_, respectively

^b1^H and ^13^C NMR data were recorded at 500 MHz and 125 MHz in CDCl_3_, respectively

^c1^H and ^13^C NMR data were recorded at 600 MHz and 150 MHz in Acetone-*d*_6_, respectively

Xylariahgin B (**2**) was obtained as colorless oil. Its IR absorptions at 3422, 2932, 1657 and 1595 cm^−1^ implied the presence of OH, COOH, lactone and C=C groups. Its HRESIMS displayed a molecular ion peak [M+Na]^+^ at *m*/*z* 249.0730 (calcd for 249.0733), corresponding to the molecular formula C_11_H_14_O_5_. Comparison of its ^1^H and ^13^C NMR spectra (Table 1) with those of **1** indicated that these two compounds were closely related. The only difference was that **2** had less one degree of unsaturation than **1**. Furthermore, the HMBC correlations from H_3_-11 (*δ*_H_ 0.96, t, 7.4) to C-10 and C-9, H_2_-10 (*δ*_H_ 1.70, m; 1.58, m) to C-9, C-8 and C-12, H_2_-8 (*δ*_H_ 2.13, m; 1.96, m) to C-10, C-9, C-12 and C-4 suggested that the furanone ring of **1** was opened in **2** (Fig. 2). Above data together with ^1^H-^1^H COSY and other key HMBC correlations from H-5 (*δ*_H_ 6.34, dd, 5.8, 2.4) to C-6 and C-3, from H-3 (*δ*_H_ 6.59, d, 2.4) to C-4, C-7 and C-2, from H-6 (*δ*_H_ 7.77, d, 5.8) to C-4, C-2 and C-5 elucidated the planar structure of **2.** The relative configuration of **2** was determined by a hydrolysis reaction of **1**. **1** was exposed to K_2_CO_3_/MeOH at room temperature for 4 h, with HPLC–DAD analysis detecting conversion to **2** (Fig. S31).

Xylariahgin C (**3**) was found by HRESIMS ([M+Na]^+^ *m/z* 263.0884, calcd for 263.0890) to possess the molecular formula C_12_H_16_O_6_ as determined. Its IR absorptions at 3395, 1732 and 1660 cm^−1^ implied the presence of OH, lactone and C=C groups. The ^1^H and ^13^C NMR spectra (Table 1) of **3** were similar to those of **2** except that there was one more methoxy group in compound **3**. The HMBC correlation from CH_3_O-12 to C-12 verified the presence of methoxy signal. The ^1^H–^1^H COSY correlations of H_3_-11/H_2_-10/H-9/H_2_-8/H-7 and H-5/H-6, coupled with the key HMBC correlations from H-8 (*δ*_H_ 2.15, m; 1.93, m) to C-4 and C-12, and from H-10 (*δ*_H_ 1.62, m) to C-12 determined the structure of **3** (Fig. 2). Similarly, the relative configuration of **3** was also supported by the hydrolysis reaction of **1.** Similar treatment of **1** with K_2_CO_3_/MeOH resulted in conversion to **2** and **3**, simultaneously. Therefore, the relative configuration of **3** was confirmed (Fig. S31).

Xylariahgin D (**4**) was found to have the molecular formula C_11_H_14_O_4_ established by HRESIMS at *m/z* 233.0784 [M+Na]^+^ (calcd for 233.0784), indicating five degrees of unsaturation. Its IR absorptions at 1779 and 1671 cm^−1^ implied the presence of lactone and C=C groups. The ^1^H NMR spectrum (Table 1) showed typical signals assignable to one methyl at *δ*_H_ (1.01, t, 7.5), one olefinic methine at *δ*_H_ (5.61, s). The ^13^C NMR and DEPT spectra of **4** displayed resonances for 11 carbons, ascribed to one methyls, four methylenes (including one oxygenated methylene), three methines (one olefinic, one oxygenated methine), and three quaternary carbons (two carbonyls). These data accounted for all ^1^H and ^13^C NMR resonances and indicated **4** is a polyketide. The ^1^H-^1^H COSY correlations of H_2_-5/H_2_-6, suggested the connection from C-5 to C-6. The detailed planar structure of **4** was further constructed by the HMBC correlations. The HMBC correlations from H-3 (*δ*_H_ 5.61, s) to C-2, C-4 and C-5, H_2_-5 (*δ*_H_ 2.60, m) to C-4, and H_2_-6 (*δ*_H_ 4.56, m) to C-4, C-2, indicated the presence of pyrone. In the HMBC spectrum of **4**, correlations from H_2_-8 (*δ*_H_ 2.62, m; 1.96, m) to C-7, C-12 and C-10, coupled with the ^1^H–^1^H COSY correlations of H-7/H_2_-8/H-9/H_2_-10/H_3_-11 proved the presence of furanone moiety (Fig. 2).

In **4**, the pyrone moiety with the connection to furanone moiety was confirmed from key HMBC correlations from H-7 to C-2, C-3, and H-8 to C-2. Herein, the structure of pyranone derivative was elucidated. In addition, there was no obvious ROESY correlation of H-7/H-9, indicative of their opposite oriented, and H-7 were arbitrarily assigned as *β*-oriented, H-9 were arbitrarily assigned as *α*-oriented.

Xylariahgin E (**5**) was isolated as yellowish oil, and its molecular formula was established to be C_13_H_18_O_5_ by HRESIMS at *m/z* 253.1081 [M−H]^−^ (calcd for 253.1087). Its IR absorptions at 2928, 1773 and 1745 cm^−1^ implied the presence of lactone and C=C groups. Comparison of the ^1^H and ^13^C NMR data (Table 2) of compound **5** with that of 2-carboxymethyl-3-hexylmaleic anhydride indicated that both compounds had identical skeleton and substitution patterns, differing only in that compound **5** was esterified in C-6 [20]. This conclusion was verified by the key HMBC correlations from H_2_-5 (*δ*_H_ 3.52, s) to C-1 and C-6, from H_2_-7 (*δ*_H_ 2.48, m) to C-4, and from 6-OMe (*δ*_H_ 3.75, s) to C-6 (Fig. 2). Herein, **5** was assigned as a new dialkylmaleic anhydride devirative.

**Table 2:** NMR data of **5**-**8** (*δ* in ppm, *J* in Hz)

No	**5** ^b^		No	**6** ^b^		No	**7** ^a^		No	**8** ^c^	
	*δ*_H_	*δ*_C_		*δ*_H_	*δ*_C_		*δ*_H_	*δ*_C_		*δ*_H_	*δ*_C_
1		165.2, s	1		170.2, s	1		167.8, s	1		125.3, s
2		136.2, s	2	3.49, 2H (s)	28.8, t	2		103.5, s	2	8.92, 1H (d, 8.7)	131.1, d
3		147.7, s	3		132.8, s	3		164.8, s	3	7.77, 1H (d, 8.7)	119.1, d
4		165.2, s	4	2.42, 2H (t, 7.5)	24.2, t	4	6.58, 1H (d, 1.9)	99.7, d	4		144.7, s
5	3.52, 2H (s)	29.2, t	5	1.52, 2H (m)	28.0, t	5		162.2, s	5	7.77, 1H (d, 8.7)	119.1, d
6		167.7, s	6	1.30, 2H (overlap)	29.2, t	6	6.55, 1H (d, 1.9)	101.5, d	6	8.92, 1H (d, 8.7)	131.1, d
7	2.48, 2H (m)	24.9, t	7		145.3, s	7		144.1, s	7		166.8, s
8	1.58, 2H (m)	27.5, t	8	1.28, 2H (overlap)	31.3, t	8	6.41, 1H (s)	106.8, d	7-OMe	3.82, 3H (s)	52.0, q
9	1.33, 2H (overlap)	29.1, t	9	1.29, 2H (overlap)	22.4, t	9		157.2, s	1′		172.5, s
10	1.30, 2H (overlap)	31.3, t	10	0.85, 3H (t, 6.4)	14.0, q	10	2.53, 1H (dd, 4.7, 8.7)	38.8, t	2′	2.38, 2H (t, 7.5)	37.7, t
11	1.31, 2H (overlap)	22.4, t	1′		174.2, s		2.74, 1H (dd, 4.7, 4.2)		3′	1.67, 2H (m)	25.7, t
12	0.88, 3H (t, 6.9)	14.0, q	2′		172.8, s	11	4.02, 1H (m)	70.8, d	4′	1.33, 2H (m)	32.1, t
6-OMe	3.75, 3H (s)	52.9, q	3′	4.27, 2H (s)	38.7, t	12	3.55, 2H (d, 5.2)	67.0, t	5′	1.31, 2H (m)	23.0, t
			4′		170.3, s	3-OMe	3.90, 3H (s)	56.5, q	6′	0.87, 3H (t, 7.0)	14.2, q
						5-OMe	3.91, 3H (s)	56.7, q			

The assignments were based on HSQC, ^1^H-^1^H COSY, and HMBC experiments

^a1^H and ^13^C NMR data were recorded at 600 MHz and 150 MHz in CD_3_OD, respectively

^b1^H and ^13^C NMR data were recorded at 500 MHz and 125 MHz in CDCl_3_, respectively

^c1^H and ^13^C NMR data were recorded at 600 MHz and 150 MHz in Acetone-*d*_6_, respectively

Xylariahgin F (**6**) was obtained as a yellowish amorphous powder and gave a HRESIMS ion peak at *m/z* 320.1108 ([M+Na]^+^, calcd for 320.1105), which corresponded to a molecular formula of C_14_H_19_NO_6_ with six degrees of unsaturation. Its IR absorptions at 2928, 1717 and 1429 cm^−1^ implied the presence of COOH, C=O and C=C groups. The ^1^H and ^13^C NMR spectra (Table 2) exhibited 14 carbon resonances including one methyl (*δ*_C_ 14.0), seven methylenes (*δ*_C_ 22.4, 24.2, 28.0, 28.8, 29.2, 31.3, and 38.7), two olefinic quaternary carbons (*δ*_C_ 132.8, 145.3), four carbonyl carbons (*δ*_C_ 170.2, 170.3, 172.8, and 174.2). The ^1^H-^1^H COSY correlations of H_2_-4/H_2_-5/H_2_-6, and H_2_-9/H_3_-10 were observed, HMBC correlations from H_3_-10 (*δ*_H_ 0.85, t, 6.4) to C-9 and C-8, from H_2_-4 (*δ*_H_ 2.42, t, 7.5) to C-3, C-5 and C-7, from H-5 (*δ*_H_ 1.52, m) to C-7, from H-2 (*δ*_H_ 3.49, s) to C-1, C-3 and C-7 suggested compound **6** was a 2-propylcyclopenteneacetic acid derivative. The HMBC correlations of H_2_-3′ (*δ*_H_ 4.27, s) with C-2′ and C-4′, revealed compound **6** was a propanedioic acid derivative (Fig. 2). Herein, the structure of two units was elucidated, and suggested compound **6** was a monocyclic polyketide derivative. The rest of units and the connections of the all the units in **6** was supported by EI^+^ mass spectrum. In EI^+^ mass spectrum, it seemed certain that *m/z* 297 is the molecular ion. The spectrum displayed the fragment ion *m/z* 252 due to the loss of COOH on the base of the molecular ion. And the ion at *m/z* 209 was strong in spectrum, probably was a daughter of *m/z* 251 via loss of C_2_H_2_O. The major fragment ions at *m/z* 123, 169, 195 which were due to the processes (M^+^-COOCONHCOCH_2_COOH), (M^+^-CONHCOCH_2_COOH + 2H^+^), (M^+^-NHCOCH_2_COOH), respectively. Then the peak at *m/z* 181 was a daughter of *m/z* 195 by means of losing CH_2_. Similarly, the ion at *m/z* 137 was a daughter of *m/z* 181 via loss CO_2_. Herein, the ion *m/z* at 123, 137, 181, 252, 251, 169, and 209 in EI^+^ mass spectrum proved the presence of the fragment ion I–VII (Fig. S61). Therefore, the structure of compound **6** was elucidated.

Compound **7** had a molecular formula of C_14_H_16_O_6_ based on HRESIMS ([M+Na]^+^ *m/z* 303.0838, calcd for 303.0839). Its IR absorptions at 3416, 1711, 1602, 1570, 1457 and 1429 cm^−1^ implied the presence of OH, lactone and phenyl groups. Comparison of the ^1^H and ^13^C NMR data (Table 2) of compound **7** with that of orthosporin dimethyl ether indicated that both compounds had identical skeletons and substitution patterns, differing only in that compound **7** was hydroxylated at C-12 rather than a methyl group in orthosporin dimethyl ether [21, 22]. This conclusion was verified by the HMBC correlation from H_2_-12 (*δ*_H_ 3.55, d, 5.2) to C-11, and the ^1^H–^1^H COSY correlations of H_a_-10/H-11/H_2_-12 (Fig. 2). Accordingly, the planar structure of compound **7** was elucidated. For its absolute configuration, it was hardly to identify the configuration of **7** ([*α*] − 6.7, *c* 0.14, MeOH) by comparison with the values of specific rotation of orthosporin dimethyl ether ([*α*] +18.9, *c* 0.03, MeOH) on account of the values of specific rotation was too small to discriminate whether it was experimental error or not [15]. Morover, **7** was further subjected to the chiral chromatographic column (DAICEL AD-RH) (4.6 mm × 250 mm) to exclude the possibility of racemate. Unfortunately, studies about the absolute configuration of **7** was restricted due to the sample shortage (1.1 mg), therefore, the configuration of C-11 couldn’t be determined under the current situation. Consequently, **7** was named 3-(2,3-dihydroxypropyl)-6,8-dimethoxyisocoumarin, and defined as a new natural product, previously reported as a reactant for the total synthesis of desmethyldiaportinol [23].

Compound **8** was obtained as a yellowish solid. Its IR absorptions at 1720, 1664, 1601, 1534 and 1407 cm^−1^ implied the presence of C=O and phenyl groups. It had the molecular formula of C_14_H_19_NO_3_, established by HRESIMS ([M+H]^+^ 250.1439, calcd for C_14_H_20_NO_3_, 250.1438) with six degrees of unsaturation. The ^1^H and ^13^C NMR data (Table 2) of **8** revealed the presence of one methyl, one methoxy group, four methylenes, four methines, and four quaternary carbons. All the above data suggested that **8** is a *p*-substituted phenyl derivative containing one nitrogen. The HMBC correlations from H_2_-2′ (*δ*_H_ 2.38, t, 7.5) to C-4′ and C-1′, from H_2_-3′ (*δ*_H_ 1.67, m) to C-5′ and C-1′, from H-6′ (*δ*_H_ 0.87, t, 7.0) to C-5′ and C-4′, coupled with the H^1^–H^1^ COSY correlation of H_2_-2′/H_2_-3′ proved the presence of the hexanamide group. Furthermore, the HMBC spectrum showed the correlations from H-3 (*δ*_H_ 7.77, d, 8.7) to C-1, from H-2 (*δ*_H_ 8.92, d, 8.7) to C-4 and C-7, from CH_3_O-7 (*δ*_H_ 3.82, s) to C-7 and C-1, corresponding with the H^1^–H^1^ COSY correlation of H-2/H-3 established the *p*-substituted phenyl with methoxycarbonyl group (Fig. 2). Herein, the structure of **8** was elucidated, and named as methyl 4-(hexanoylamino) benzoate. **8** was defined as a new natural product, previously reported as a reactant for the preparation of oxindole hydrazide modulators of protein tyrosine phosphatases [24].

The structures of known compounds (**9** and **10**) were identified by comparison of their spectroscopic data with the literature as (−)-5-carboxylmellein (**9**) [25], 4-methoxyisoquinolin-1(2*H*)-one (**10**) [26], respectively.

Compounds **1**–**8** were tested for their cytotoxicity against HL-60, A-549, SMMC-7721, MCF-7, and SW-480 human tumor cell lines by MTS methods using cisplatin and paclitaxel as positive controls. Neither of them showed cytotoxic activity against all cell lines.

## Experimental

### General

Optical rotations were measured in MeOH with JASCO P-1020 polarimeters. 1D and 2D NMR spectra were recorded on Advance III 500 or 600 spectrometers using pyridine-*d*_5_ as the internal standard. Chemical shifts (*δ*) are expressed in ppm relative to the pyridine-*d*_5_ signals. HRESIMS was performed on an API QSTAR spectrometer. UV spectra were obtained on a Shimadzu UV-2401PC spectrophotometer. IR spectra were obtained on a Bruker Tensor-27 FT-IR spectrometer using KBr pellets. Column chromatography (CC) was performed with silica gel (100–200 mesh; Qingdao Marine Chemical, Inc., Qingdao, People’s Republic of China), MCI gel (CHP20P, 75–150 μm, Mitsubishi Chemical Corporation, Tokyo, Japan). Semi-preparative HPLC was performed on an Agilent 1200 liquid chromatograph with a Zorbax SB-C18 (9.4 mm × 250 mm) column. Fractions were monitored by thin layer chromatography, spots were visualized by UV light (254 nm and 365 nm) and by heating silica gel plates sprayed with 10% H_2_SO_4_ in EtOH. All solvents used in column chromatography were distilled including petroleum ether.

### Fungal Material and Identification

The fungal strain of *Xylaria* sp. hg1009 was isolated from fresh stems of *I*. *sculponeatus* collected from Weishan, DaLi, People’s Republic of China, in December 2012. Fungal identification was based on sequence analysis (GenBank Accession No. MG739623) of internal transcribed spacer (ITS) regions of the rDNA.

### Fermentation, Extraction and Isolation

The fungus was cultivated in solid rice medium in 135 Fernbach flasks (500 mL, 60 mL distilled water was added to 80 g rice and kept overnight before autoclaving) for 40 days at 28 °C in a static incubator. The fungus was cultivated in 200 mL MEA liquid medium in 58 Erlenmeyer flasks (500 mL) for two weeks at 28 °C on a rotary shaker at 180 rpm.

The rice medium was overlaid and extracted with 70% aqueous acetone. The solvent was then evaporated in vacuo to afford a crude extract (80 g). The extraction was subjected to column chromatography on silica gel with a CHCl_3_/Me_2_CO gradient system (1:0, 9:1, 8:2, 7:3, 6:4, 1:1, 0:1) to yield seven fractions, A–G. Fraction A (CHCl_3_/Me_2_CO 1:0, 12 g) was subjected to column chromatography on silica gel with a petroleum ether/CH_3_CH_2_OAc gradient system (from 10:1 to 0:1) to afford fractions A1–A9, fraction A7 was purified by semipreparative HPLC to yield **1** (25 mg), fraction A8 was purified by semipreparative HPLC to yield **9** (2.3 mg) and **10** (1.9 mg).

Fraction B (CHCl_3_/Me_2_CO 9:1, 19 g) was chromatographed on RP-18 with MeOH/H_2_O (30:70, 40:60, 50:50, 60:40, 70:30, 100:0) to obtain B1-B11, B3 (65 mg) and B4 (80 mg)was purified by semipreparative HPLC to afford **2** (5.9 mg), **3** (30 mg), and **7** (1.1 mg), respectively. B8 (1.2 g) chromatographed on a silica gel column with a petroleum ether/Me_2_CO gradient system (from 20:1 to 1:1) to afford fractions B8-1 to B8-7. Fraction B8-5 was purified by preparative HPLC to yield **4** (3.5 mg). Fraction C (CHCl_3_/Me_2_CO 8:2, 15 g) was purified by silica gel column with a petroleum ether/CH_3_CH_2_OAc gradient system (from 10:1 to 1:2) to afford fractions C1-C9. Fraction C6 was purified by preparative HPLC yield subfractions C6-1 to C6-5, then subfraction C6-2 were purified by semipreparative HPLC to yield **6** (60 mg).

The MEA culture broth was extracted with EtOAc 3 times to yield a crude extract (3.2 g). The extraction was subjected to RP-18 with MeOH/H_2_O (20:80, 30:70, 50:50, 70:30, and 100:0) to yield five fractions, A-E. Fraction B (0.6 g) was chromatographed on a silica gel column with a petroleum ether/Me_2_CO gradient system (from 10:1 to 0:1) to afford fractions B1-B3. Fraction B1 was subsequently purified by semipreparative HPLC to yield **5** (1.4 mg) and **8** (2.1 mg).

### The Cytotoxicity Assay

The human tumor cell lines HL-60, SMMC-7721, A-549, MCF-7, and SW-480 were used in the cytotoxic assay. These cell lines were obtained from ATCC (Manassas, VA, USA). Cells were cultured in RMPI-1640 or DMEM medium (Biological Industries, Kibbutz Beit-Haemek, Israel) supplemented with 10% fetal bovine serum (Biological Industries) at 37 °C in a humidified atmosphere with 5% CO_2_. The cytotoxicity assay was evaluated by the 3-(4,5-dimethylthiazol-2-yl)-5-(3-carboxymethoxyphenyl)-2-(4-sulfophenyl)-2H-tetrazolium, inner salt (MTS) (Promega, Madison, WI, USA) assay [27]. Briefly, cells were seeded into each well of a 96-well cell culture plate. After 12 h of incubation at 37 °C, the test compound (40 μM) was added. After incubated for 48 h, cells were subjected to the MTS assay [28, 29]. Compounds with a growth inhibition rate of 50% were further evaluated at concentrations of 0.064, 0.32, 1.6, 8, and 40* μ*M in triplicate, with cisplatin and paclitaxel (Sigma, St. Louis, MO, USA) as positive controls. The IC_50_ value of each compound was calculated with Reed and Muench’s method [30].

Xylariahgin A (**1)** yellow oil. [*α*]_D_^23^ − 56.9 (*c* 0.18, MeOH), UV (MeOH) *λ*_max_ (log *ε*) 414 (1.70), 327 (2.48), 248 (3.97), 207 (3.86) nm, IR (KBr) *v*_max_ 2967, 2878, 1781, 1663, 1623, 1414, 1386, 1342, 1153, 1029, 956, 931, 871 cm^−1^. ESIMS *m/z* 231 [M+Na]^+^, HRESIMS *m/z* 231.0624 [M+Na]^+^ (calcd for C_11_H_12_O_4_Na 231.0628). ^1^H NMR (Acetone-*d*_6_, 500 MHz) and ^13^C NMR (Acetone-*d*_6_, 125 MHz), see Table 1.

Xylariahgin B (**2)** colorless oil. [*α*]_D_^20^ − 20.0 (*c* 0.18, MeOH), UV (MeOH) *λ*_max_ (log *ε*) 248 (3.97), 208 (3.83) nm, IR (KBr) *v*_max_ 3422, 2932, 1657, 1595, 1422, 1384, 1328, 1236, 1103, 935, 874 cm^−1^. ESIMS *m/z* 249 [M+ Na]^+^, HRESIMS *m/z* 249.0730 [M+Na]^+^ (calcd for C_11_H_14_O_5_Na 249.0733). ^1^H NMR (CDCl_3_, 500 MHz) and ^13^C NMR (CDCl_3_, 125 MHz), see Table 1.

Xylariahgin C (**3)** yellow oil. [*α*]_D_^24^ − 23.1 (*c* 0.14, MeOH), UV (MeOH) *λ*_max_ (log *ε*) 248 (4.15), 207 (3.99) nm, IR (KBr) *v*_max_ 3395, 2964, 2933, 1783, 1732, 1660, 1422, 1384, 1157, 1103, 933, 873 cm^−1^. ESIMS *m/z* 263 [M+Na]^+^, HRESIMS *m/z* 263.0884 [M + Na]^+^ (calcd for C_12_H_16_O_6_Na 263.0884). ^1^H NMR (Acetone-*d*_6,_ 600 MHz) and ^13^C NMR (Acetone-*d*_6_, 150 MHz), see Table 1.

Xylariahgin D (**4)** yellow oil. [*α*]_D_^23^ − 44.9 (*c* 0.10, MeOH), UV (MeOH) *λ*_max_ (log *ε*) 295 (3.07), 260 (3.98) nm, IR (KBr) *v*_max_ 2966, 2934, 2878, 1779, 1671, 1618, 1464, 1384, 1291, 1229, 1186, 1163, 1076, 984 cm^−1^. ESIMS *m/z* 233 [M + Na]^+^, HRESIMS *m/z* 233.0784 [M + Na]^+^ (calcd for C_11_H_14_O_4_Na 233.0784). ^1^H NMR (CDCl_3_, 500 MHz) and ^13^C NMR (CDCl_3_, 125 MHz), see Table 1.

Xylariahgin E (**5)** yellowish oil. UV (MeOH) *λ*_max_ (log *ε*) 206 (3.94) nm, IR (KBr) *v*_max_ 2956, 2928, 2856, 1773, 1745, 1582, 1384, 1275, 1174, 924, 765 cm^−1^. ESIMS *m/z* 253 [M−H]^−^, HRESIMS *m/z* 253.1081 [M−H]^−^ (calcd for C_13_H_17_O_5_ 253.1087). ^1^H NMR (CDCl_3_, 500 MHz) and ^13^C NMR (CDCl_3_, 125 MHz), see Table 2.

Xylariahgin F (**6)** yellowish amorphous power. UV (MeOH) *λ*_max_ (log *ε*) 225 (4.15) nm, IR (KBr) *v*_max_ 2928, 2860, 1717, 1429, 1313, 1194, 1124, 980, 944, 765, 616 cm^−1^. ESIMS *m/z* 320 [M+Na]^+^, HRESIMS *m/z*320.1108 [M+Na]^+^ (calcd for C_14_H_19_NO_6_Na 320.1105). ^1^H NMR (CDCl_3_, 500 MHz) and ^13^C NMR (CDCl_3_, 125 MHz), see Table 2.

3-(2,3-Dihydroxypropyl)-6,8-dimethoxyisocoumarin (**7**): yellowish solid. [*α*]_D_^24^ − 6.7 (*c* 0.14, MeOH). UV (MeOH) *λ*_max_ (log *ε*) 323 (3.74), 287 (3.72), 278 (3.77), 242 (4.55) nm. IR (KBr) *v*_max_ 3416, 2928, 2854, 1711, 1602, 1570, 1457, 1429, 1382, 1246, 1217, 1205, 1165, 1061 cm^−1^. ESIMS *m/z* 303 [M+Na]^+^, HRESIMS *m/z* 303.0838 [M+Na]^+^ (calcd for C_14_H_16_O_6_Na 303.0839). ^1^H NMR (CD_3_OD, 600 MHz) and ^13^C NMR (CD_3_OD, 150 MHz), see Table 2.

Methyl 4-(hexanoylamino) benzoate (**8**): yellowish solid. UV (MeOH) *λ*_max_ (log *ε*) 272 (4.44) nm. IR (KBr) *v*_max_ 2955, 2930, 1720, 1664, 1601, 1534, 1407, 1281, 1175, 1111, 1019, 771 cm^−1^. ESIMS *m/z* 250 [M+H]^+^, HRESIMS *m/z* 250.1439 [M+H]^+^ (calcd for C_14_H_20_NO_3_ 250.1438). ^1^H NMR (Acetone-*d*_6_, 600 MHz) and ^13^C NMR (Acetone-*d*_6_, 150 MHz), see Table 2.

## Electronic supplementary material

Below is the link to the electronic supplementary material.

Supplementary material 1 (PDF 11123 kb). Supplementary data associated with this article including 1D and 2D NMR, HRESIMS, UV, IR of **1**–**8** are available
