# Supplementary material for: Secondary Metabolites from the Endophytic Fungus Xylaria sp. hg1009
Source: Nat Prod Bioprospect. 2018 Mar 20;8(2):121–9. doi: 10.1007/s13659-018-0158-x (PMC5913051; doi:10.1007/s13659-018-0158-x)

Supplementary data for

Secondary metabolites from the endophytic fungus *Xylaria* sp. hg1009

Rong Chen[a,b], Jian-Wei Tang[b,c], Xing-Ren Li[b], Miao Liu[b], Wen-Ping Ding[a,b], Yuan-Fei Zhou[b,c], Wei-Guang Wang[b], Xue Du[b], Han-Dong Sun[b] and Pema-Tenzin Puno*[b]

[a] *School of Chemical Science and Technology, Yunnan University, Kunming 650091, People´s Republic of China*

[b] *State Key Laboratory of Phytochemistry and Plant Resources in West China*, *Kunming Institute of Botany*, C*hinese Academy of Sciences*, *Kunming 650201*, *People´s Republic of China*

[c] *University of Chinese Academy of Sciences*, *Beijing 100049*, *People´s Republic of China*

content

Fig. S1 HRESIMS spectrum of xylariahgin A (1) 4
Fig. S2 ^{1}H NMR spectrum (Acetone-d$_6$, 500 MHz) of xylariahgin A (1) 5
Fig. S3 ^{13}C NMR spectrum (Acetone-d_6, 500 MHz) of xylariahgin A (1) 6
Fig. S4 HSQC spectrum (Acetone-d$_6$, 500 MHz) of xylariahgin A (1) 7
Fig. S5 ^{1}H-^{1}H COSY spectrum (Acetone-d$_6$, 500 MHz) of xylariahgin A (1) 8
Fig. S6 HMBC spectrum (Acetone-d_6, 500 MHz) of xylariahgin A (1) 9
Fig. S7 ROESY spectrum (Acetone-d_6, 500 MHz) of xylariahgin A (1) 10
Fig. S8 UV spectrum of xylariahgin A (1) 11
Fig. S9 [α] spectrum of xylariahgin A (1) 12
Fig. S10 IR spectrum of xylariahgin A (1) 13
Fig. S11 HRESIMS spectrum of xylariahgin B (2) 14
Fig. S12 ^{1}H NMR spectrum ($CDCl_3$, 500 MHz) of xylariahgin B (2) 15
Fig. S13 ^{13}C NMR spectrum ($CDCl_3$, 500 MHz) of xylariahgin B (2) 16
Fig. S14 HSQC spectrum ($CDCl_3$, 500 MHz) of xylariahgin B (2) 17
Fig. S15 ^{1}H-^{1}HCOSY spectrum ($CDCl_3$, 500 MHz) of xylariahgin B (2) 18
Fig. S16 HMBC spectrum ($CDCl_3$, 500 MHz) of xylariahgin B (2) 19
Fig. S17 ROESY spectrum ($CDCl_3$, 500 MHz) of xylariahgin B (2) 20
Fig. S18 UV spectrum of xylariahgin B (2) 21
Fig. S19 [α] spectrum of xylariahgin B (2) 22
Fig. S20 IR spectrum of xylariahgin B (2) 23
Fig. S21 HRESIMS spectrum of xylariahgin C (3) 24
Fig. S22 ^{1}H NMR spectrum (Acetone-d_6, 600 MHz) of xylariahgin C (3) 25
Fig. S23 ^{13}C NMR spectrum (Acetone-d_6, 600 MHz) of xylariahgin C (3) 26
Fig. S24 HSQC spectrum (Acetone-d_6, 600 MHz) of xylariahgin C (3) 27
Fig. S25 ^{1}H-^{1}H COSY spectrum (Acetone-d_6, 600 MHz) of xylariahgin C (3) 28
Fig. S26 HMBC spectrum (Acetone-d_6, 600 MHz) of xylariahgin C (3) 29
Fig. S27 ROESY spectrum (Acetone-d_6, 600 MHz) of xylariahgin C (3) 30
Fig. S28 UV spectrum of xylariahgin C (3) 31
Fig. S29 [α] spectrum of xylariahgin C (3) 32

Fig. S30 IR spectrum of xylariahgin C (3) 33
Fig. S31 HPLC profiles of xylariahgins A-C (1-3) 34
Fig. S32 HRESIMS spectrum of xylariahgin D (4) 35
Fig. S33 ^{1}H NMR spectrum ($CDCl_3$, 500 MHz) of xylariahgin D (4) 36
Fig. S34 ^{13}C NMR spectrum ($CDCl_3$, 500 MHz) of xylariahgin D (4) 37
Fig. S35 HSQC spectrum ($CDCl_3$, 500 MHz) of xylariahgin D (4) 38
Fig. S36 ^{1}H-^{1}H COSY spectrum ($CDCl_3$, 500 MHz) of xylariahgin D (4) 39
Fig. S37 HMBC spectrum ($CDCl_3$, 500 MHz) of xylariahgin D (4) 40
Fig. S38 ROESY spectrum ($CDCl_3$, 500 MHz) of xylariahgin D (4) 41
Fig. S39 UV spectrum of xylariahgin D (4) 42
Fig. S40 [α] spectrum of xylariahgin D (4) 43
Fig. S41 IR spectrum of xylariahgin D (4) 44
Fig. S42 HRESIMS spectrum of xylariahgin E (5) 45
Fig. S43 ^{1}H NMR spectrum ($CDCl_3$, 500 MHz) of xylariahgin E (5) 46
Fig. S44 ^{13}C NMR spectrum ($CDCl_3$, 500 MHz) of xylariahgin E (5) 47
Fig. S45 HSQC spectrum ($CDCl_3$, 500 MHz) of xylariahgin E (5) 48
Fig. S46 ^{1}H-^{1}H COSY spectrum ($CDCl_3$, 500 MHz) of xylariahgin E (5) 49
Fig. S47 HMBC spectrum ($CDCl_3$, 500 MHz) of xylariahgin E (5) 50
Fig. S48 ROESY spectrum ($CDCl_3$, 500 MHz) of xylariahgin E (5) 51
Fig. S49 UV spectrum of xylariahgin E (5) 52
Fig. S50 IR spectrum of xylariahgin E (5) 533
Fig. S51 HRESIMS spectrum of xylariahgin F (6) 544
Fig. S52 ^{1}H NMR spectrum ($CDCl_3$, 500 MHz) of xylariahgin F (6) 555
Fig. S53 ^{13}C NMR spectrum ($CDCl_3$, 500 MHz) of xylariahgin F (6) 566
Fig. S54 HSQC spectrum ($CDCl_3$, 500 MHz) of xylariahgin F (6) 577
Fig. S55 ^{1}H-^{1}H COSY spectrum ($CDCl_3$, 500 MHz) of xylariahgin F (6) 588
Fig. S56 HMBC spectrum ($CDCl_3$, 500 MHz) of xylariahgin F (6) 59
Fig. S57 ROESY spectrum ($CDCl_3$, 500 MHz) of xylariahgin F (6) 60
Fig. S58 UV spectrum of xylariahgin F (6) 611
Fig. S59 IR spectrum of xylariahgin F (6) 622
Fig. S60 EI^{+} spectrum of xylariahgin F (6) 633
Fig. S61 Analysis of EI^{+} spectrum of xylariahgin F (6) 644
Fig. S62 HRESIMS spectrum of 7 655
Fig. S63 ^{1}H NMR spectrum (CD_3OD, 600 MHz) of 7 666
Fig. S64 ^{13}C NMR spectrum (CD_3OD, 600 MHz) of 7 677
Fig. S65 HSQC spectrum (CD_3OD, 600 MHz) of 7 68
Fig. S66 ^{1}H-^{1}H COSY spectrum (CD_3OD, 600 MHz) of 7 69
Fig. S67 HMBC spectrum (CD_3OD, 600 MHz) of 7 70
Fig. S68 ROESY spectrum (CD_3OD, 600 MHz) of 7 711
Fig. S69 UV spectrum of 7 722
Fig. S70 [α] spectrum of 7 733
Fig. S71 IR spectrum of 7 744
Fig. S72 HRESIMS spectrum of 8 755
Fig. S73 ^{1}H NMR spectrum (Acetone-d_6, 600 MHz) of 8 766

Fig. S74 ^{13}C NMR spectrum (Acetone-d_6, 600 MHz) of 8 777
Fig. S75 HSQC spectrum (Acetone-d_6, 600 MHz) of 8 778
Fig. S76 ^1H-^1H COSY spectrum (Acetone-d_6, 600 MHz) of 8 79
Fig. S77 HMBC spectrum (Acetone-d_6, 600 MHz) of 8 800
Fig. S78 UV spectrum of 8 811
Fig. S79 IR spectrum of 8 822

Fig. S1 HRESIMS spectrum of xylariahgin A (**1**)

Formula Predictor Report - scr60.lcd

Page 1 of 1

Data File: E:\DATA\2017\0822\scr60.lcd

Elmt	Val.	Min	Max	Elmt	Val.	Min	Max	Elmt	Val.	Min	Max	Elmt	Val.	Min	Max	Use Adduct
H	1	0	150	O	2	0	50	P	3	0	0	Br	1	0	0	Na
B	3	0	0	F	1	0	0	S	2	0	0	I	3	0	5	
C	4	0	100	Na	1	0	0	Cl	1	0	0	Pt	2	0	5	
N	3	0	20	Mg	2	0	0	Fe	2	0	0					

Error Margin (ppm): 10
HC Ratio: unlimited
Max Isotopes: all
MSn Iso RI (%): 75.00

DBE Range: -2.0 - 100.0
Apply N Rule: yes
Isotope RI (%): 1.00
MSn Logic Mode: AND

Electron Ions: both
Use MSn Info: yes
Isotope Res: 10000
Max Results: 10

Event#: 1 MS(E+) Ret. Time : 0.450 Scan# : 91

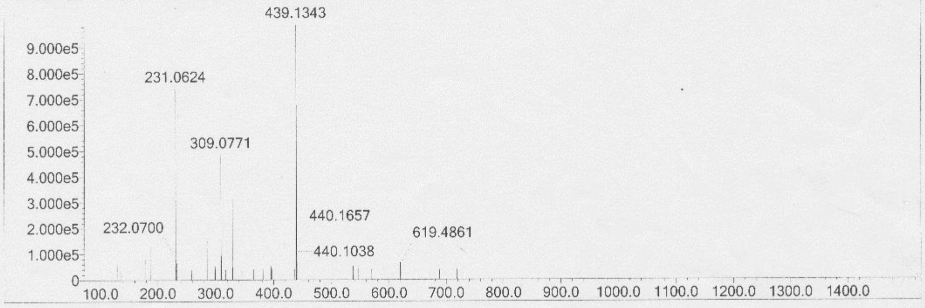

Measured region for 231.0624 m/z

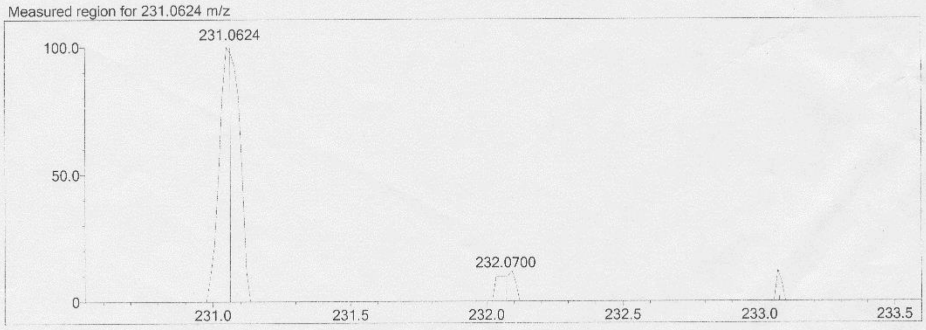

C11 H12 O4 [M+Na]+ : Predicted region for 231.0628 m/z

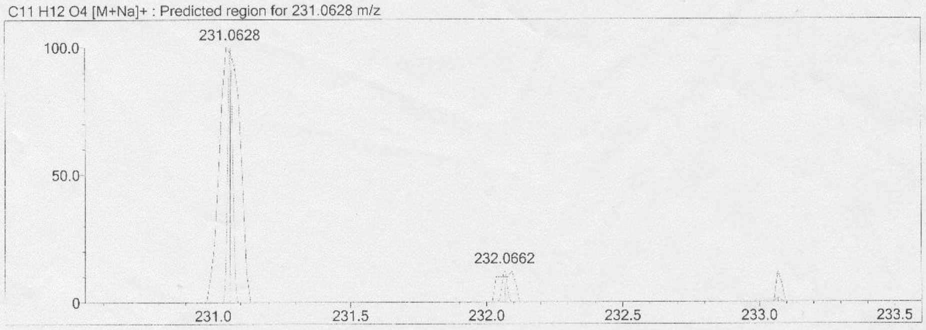

Formula (M)	Ion	Meas. m/z	Pred. m/z	Df. (mDa)	Df. (ppm)	DBE
C11 H12 O4	[M+Na]+	231.0624	231.0628	-0.4	-1.73	6.0

Fig. S2 ^{1}H NMR spectrum (Acetone-d_6, 500 MHz) of xylariahgin A (**1**)

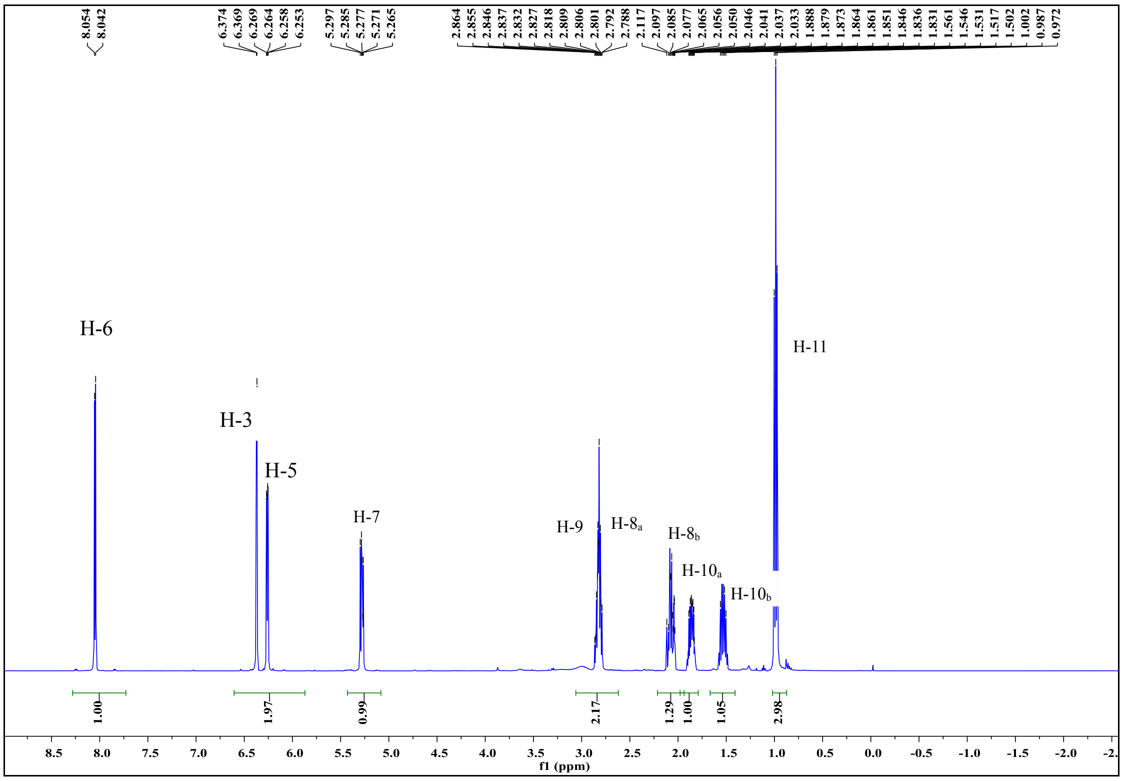

Fig. S3 ^{13}C NMR spectrum (Acetone-d_6, 500 MHz) of xylariahgin A (**1**)

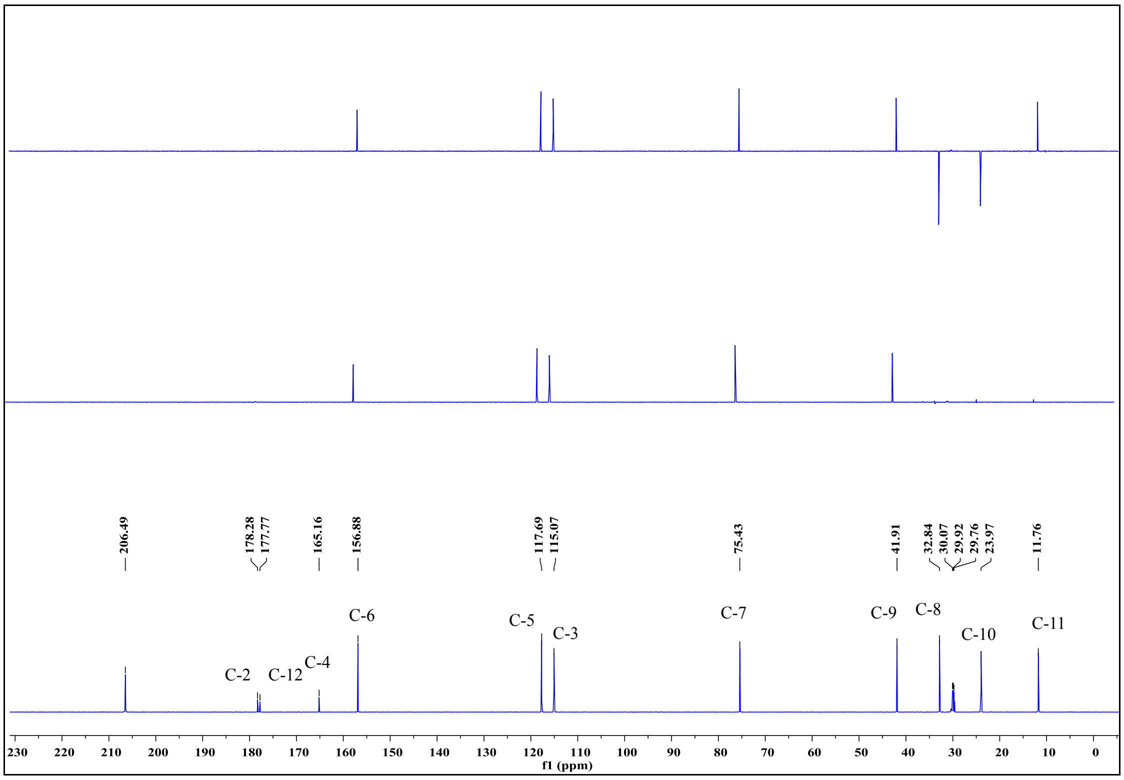

Fig. S4 HSQC spectrum (Acetone-d_6, 500 MHz) of xylariahgin A (**1**)

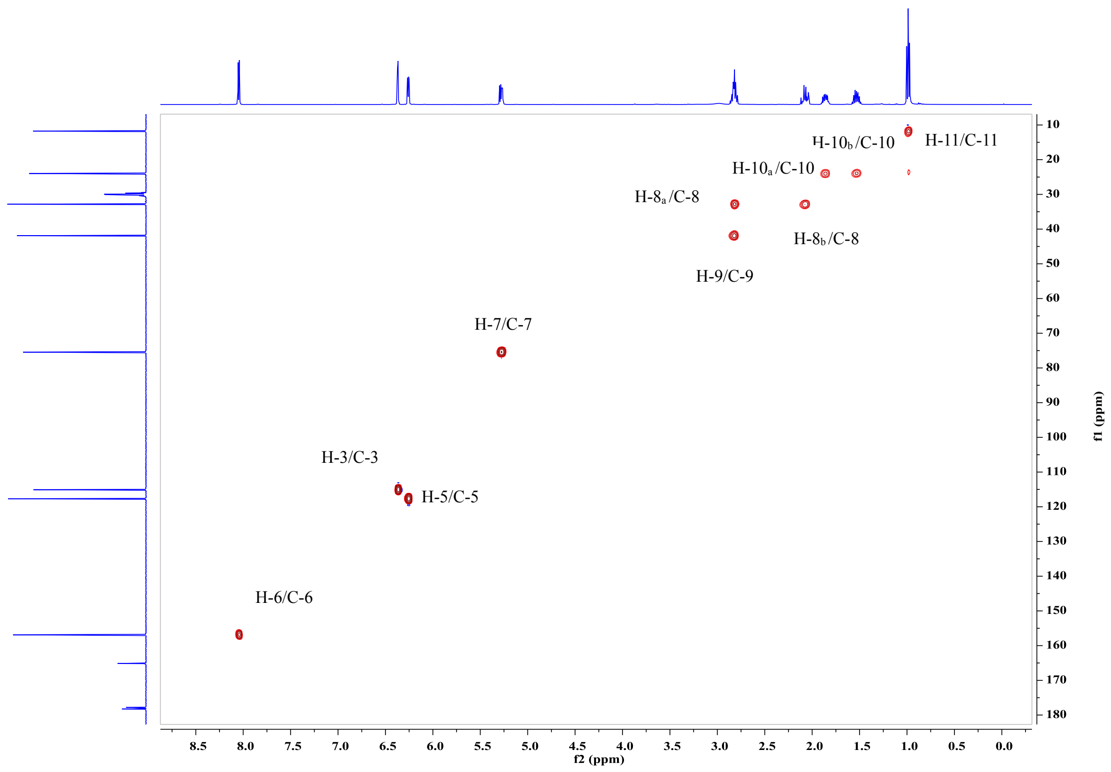

Fig. S5 ^1H-^1H COSY spectrum (Acetone-d_6, 500 MHz) of xylariahgin A (**1**)

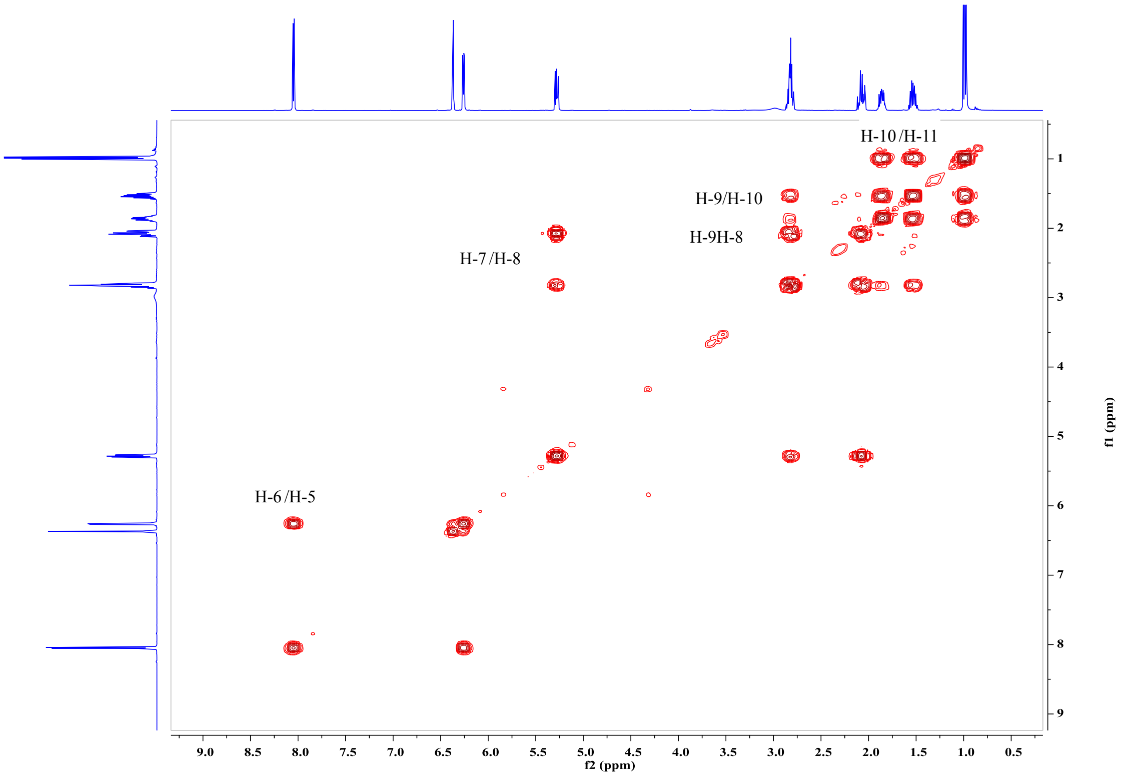

Fig. S6 HMBC spectrum (Acetone-d_6, 500 MHz) of xylariahgin A (**1**)

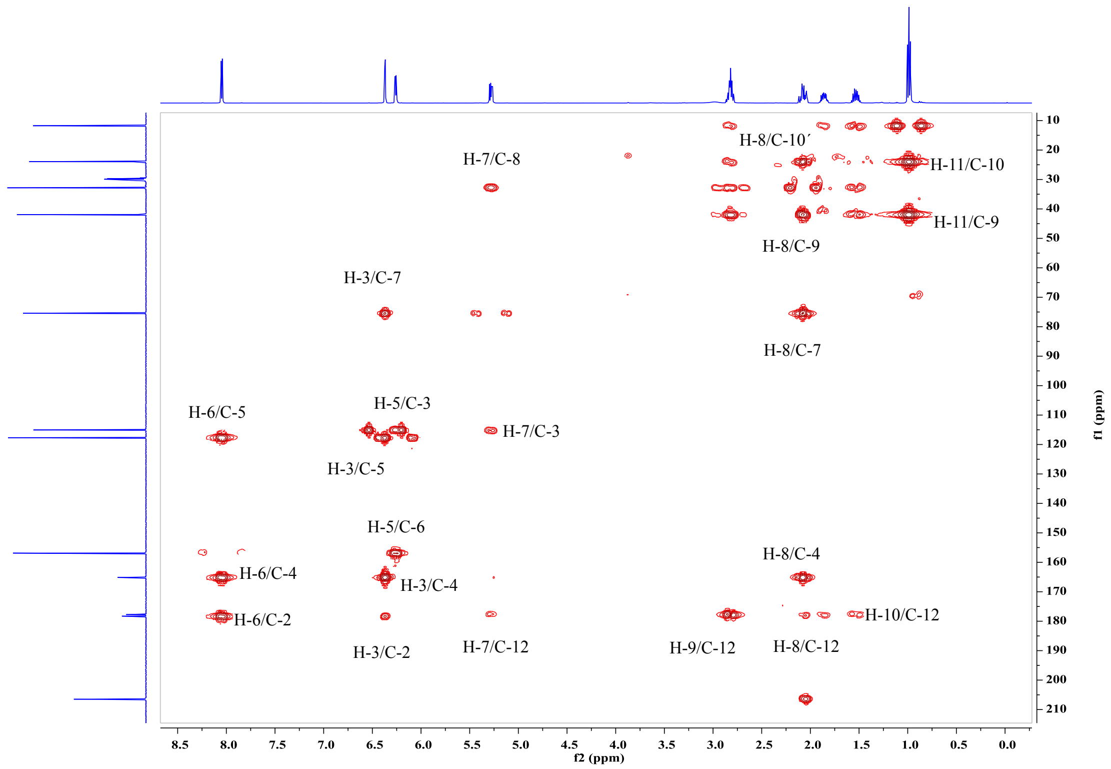

Fig. S7 ROESY spectrum (Acetone-d_6, 500 MHz) of xylariahgin A (**1**)

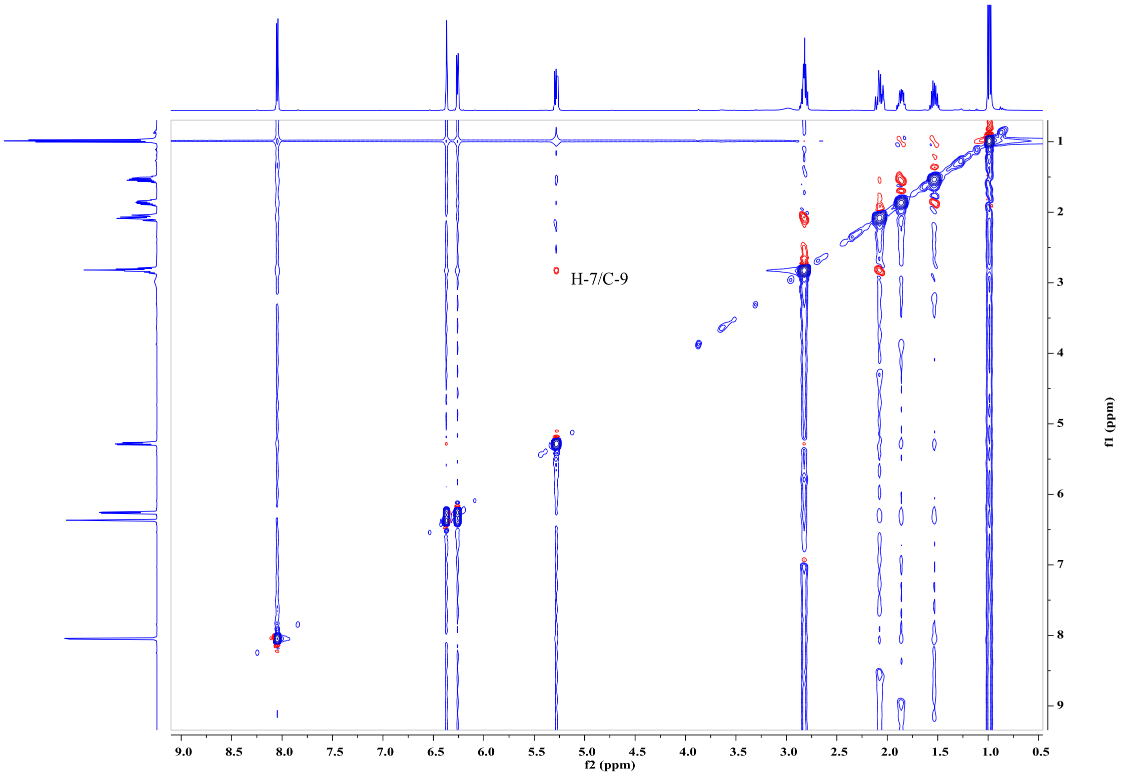

Fig. S8 UV spectrum of xylariahgin A (**1**)

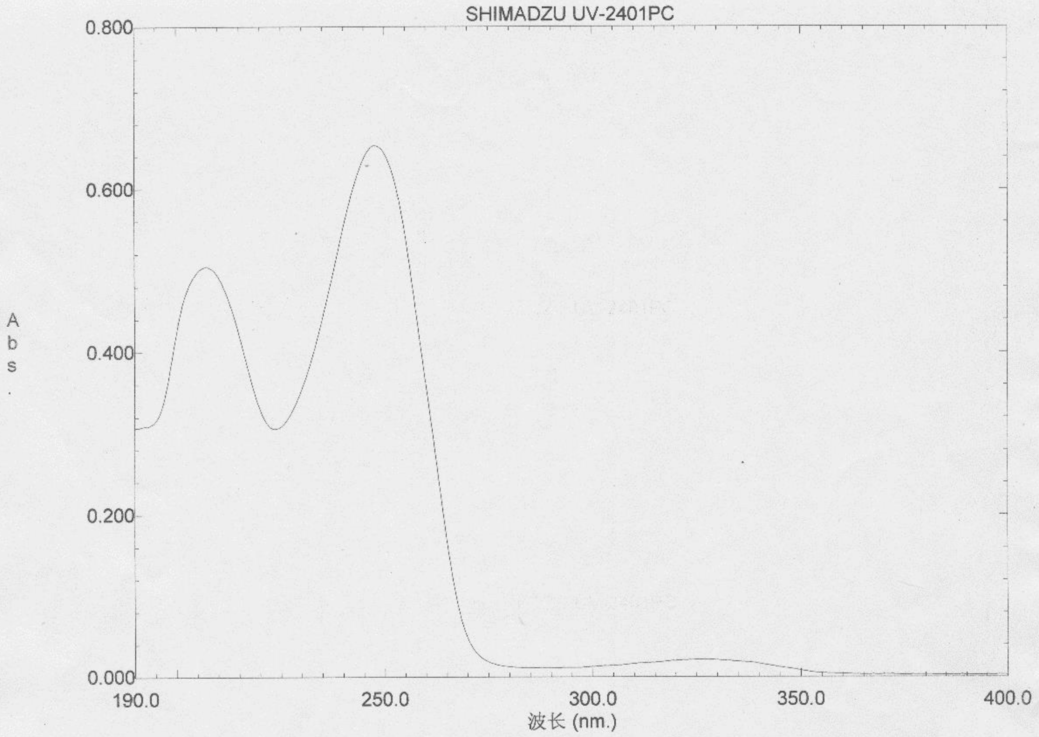

文件名: SCR60

SCR60 ———

样品浓度：0.0146毫克/毫升
溶剂：甲醇

创建于: 17:20 17-08-28
数据: 原始

测量模式: Abs.
扫描速度: 中速
狭缝: 5.0
采样间隔: 0.5

否.	波长 (nm.)	Abs.
1	414.00	0.0035
2	327.00	0.0214
3	248.00	0.6533
4	207.00	0.5043

Fig. S9 [α] spectrum of xylariahgin A (**1**)

Optical rotation measurement

Model : P-1020 (A060460638)

No.	Sample	Mode	Data	Monitor<br>Blank	Temp.<br>Cell<br>Temp Point	Date<br>Comment<br>Sample Name	Light<br>Filter<br>Operator	Cycle Time<br>Integ Time
No.1	7 (1/3)	Sp.Rot	-57.2070	-0.0512<br>0.0000	23.8<br>50.00<br>Cell	Mon Jul 24 11:41:05 2017<br>0.00179g/mL MeOH<br>SCR60	Na<br>589nm	2 sec<br>2 sec
No.2	7 (2/3)	Sp.Rot	-56.7600	-0.0508<br>0.0000	23.8<br>50.00<br>Cell	Mon Jul 24 11:41:10 2017<br>0.00179g/mL MeOH<br>SCR60	Na<br>589nm	2 sec<br>2 sec
No.3	7 (3/3)	Sp.Rot	-56.7600	-0.0508<br>0.0000	23.9<br>50.00<br>Cell	Mon Jul 24 11:41:15 2017<br>0.00179g/mL MeOH<br>SCR60	Na<br>589nm	2 sec<br>2 sec

-56.9088°

Fig. S10 IR spectrum of xylariahgin A (**1**)

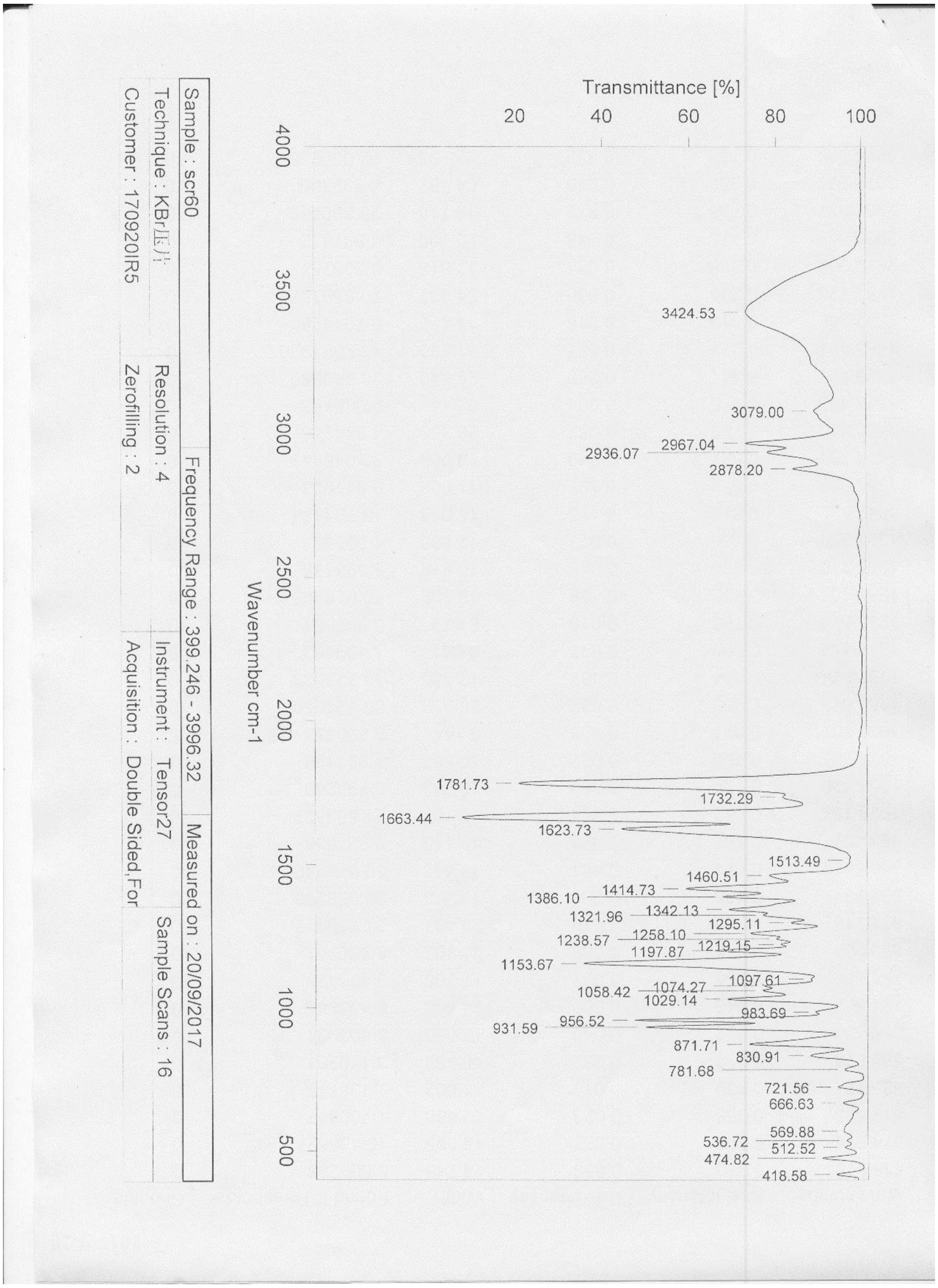

Fig. S11 HRESIMS spectrum of xylariahgin B (**2**)

Formula Predictor Report - scr62.lcd

Page 1 of 1

Data File: E:\DATA\2017\0904\scr62.lcd

Elmt	Val.	Min	Max	Elmt	Val.	Min	Max	Elmt	Val.	Min	Max	Elmt	Val.	Min	Max	Use Adduct
H	1	0	150	O	2	0	50	P	3	0	0	Br	1	0	0	Na
B	3	0	0	F	1	0	0	S	2	0	0	I	3	0	0	
C	4	0	50	Na	1	0	0	Cl	1	0	0	Pt	2	0	0	
N	3	0	0	Mg	2	0	0	Fe	2	0	0					

Error Margin (ppm): 5
HC Ratio: unlimited
Max Isotopes: all
MSn Iso RI (%): 75.00

DBE Range: -2.0 - 100.0
Apply N Rule: yes
Isotope RI (%): 1.00
MSn Logic Mode: AND

Electron Ions: both
Use MSn Info: yes
Isotope Res: 10000
Max Results: 10

Event#: 1 MS(E+) Ret. Time : 0.320 -> 0.350 Scan# : 65 -> 71

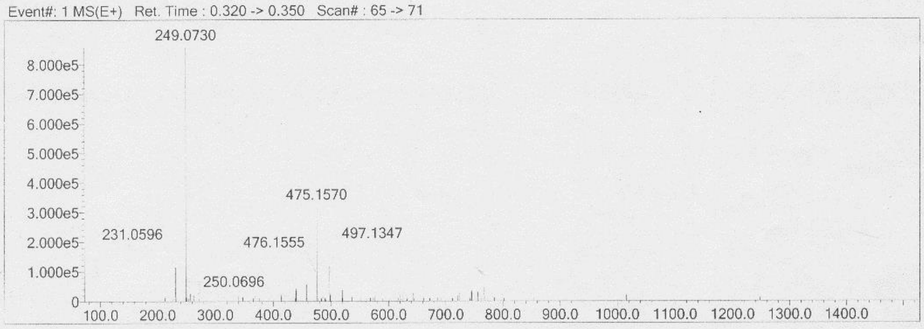

Measured region for 249.0730 m/z

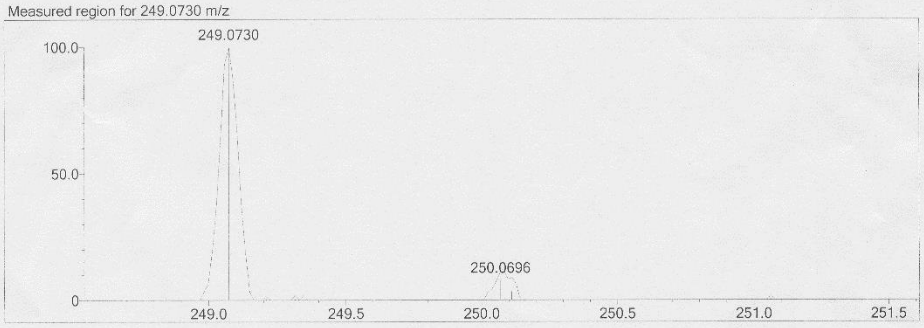

C11 H14 O5 [M+Na]+ : Predicted region for 249.0733 m/z

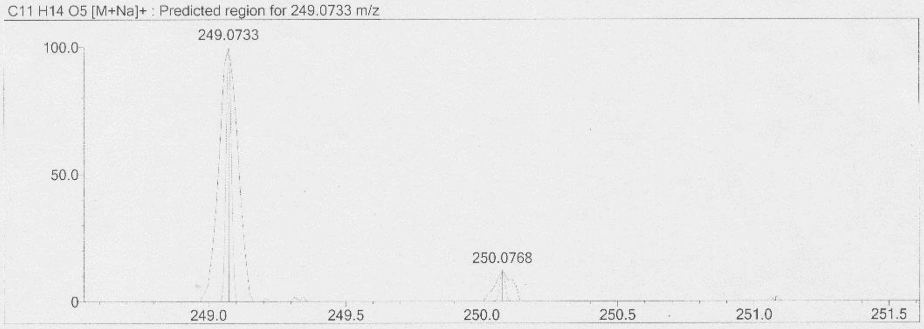

Formula (M)	Ion	Meas. m/z	Pred. m/z	Df. (mDa)	Df. (ppm)	DBE
C11 H14 O5	[M+Na]+	249.0730	249.0733	-0.3	-1.20	5.0

Fig. S12 ^{1}H NMR spectrum ($CDCl_3$, 500 MHz) of xylariahgin B (**2**)

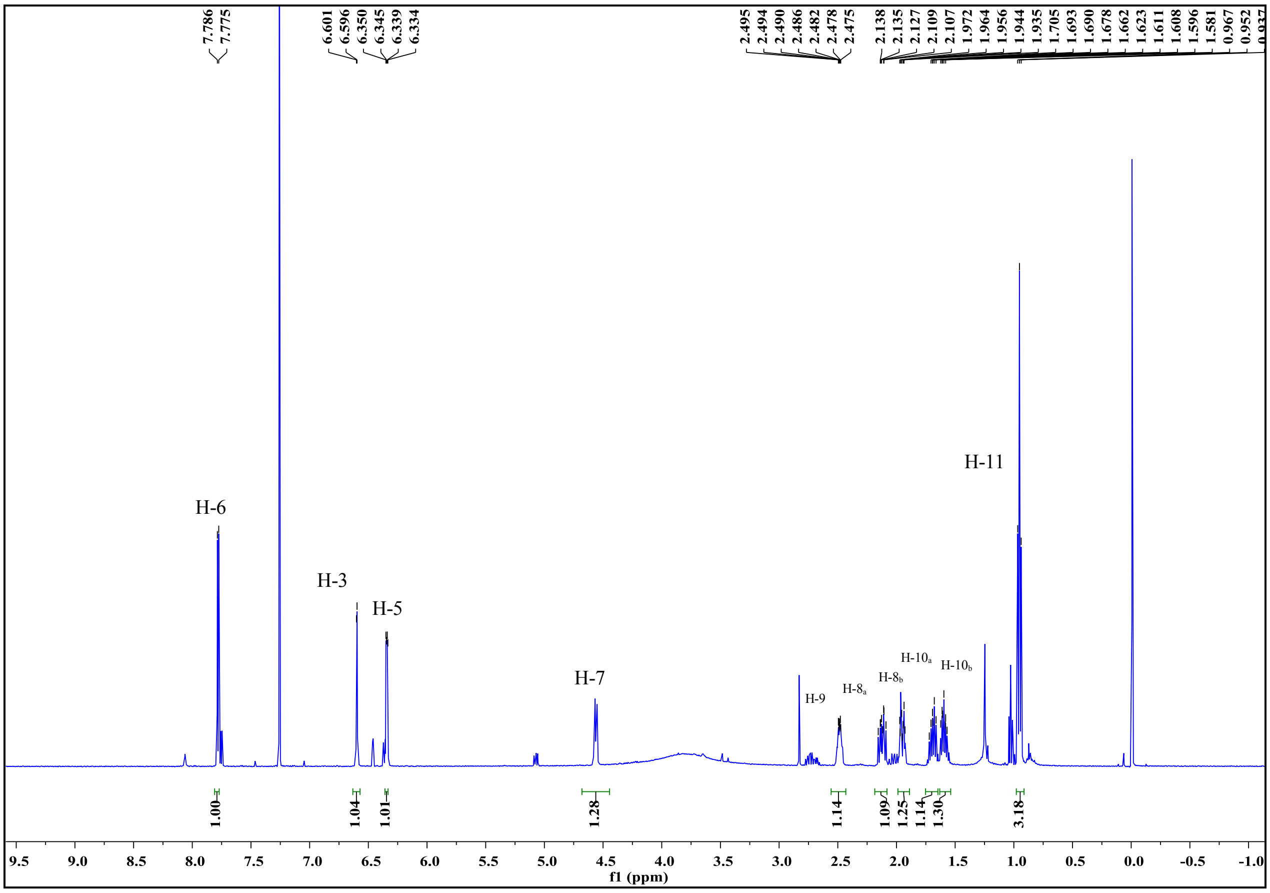

Fig. S13 ^{13}C NMR spectrum ($CDCl_3$, 500 MHz) of xylariahgin B (**2**)

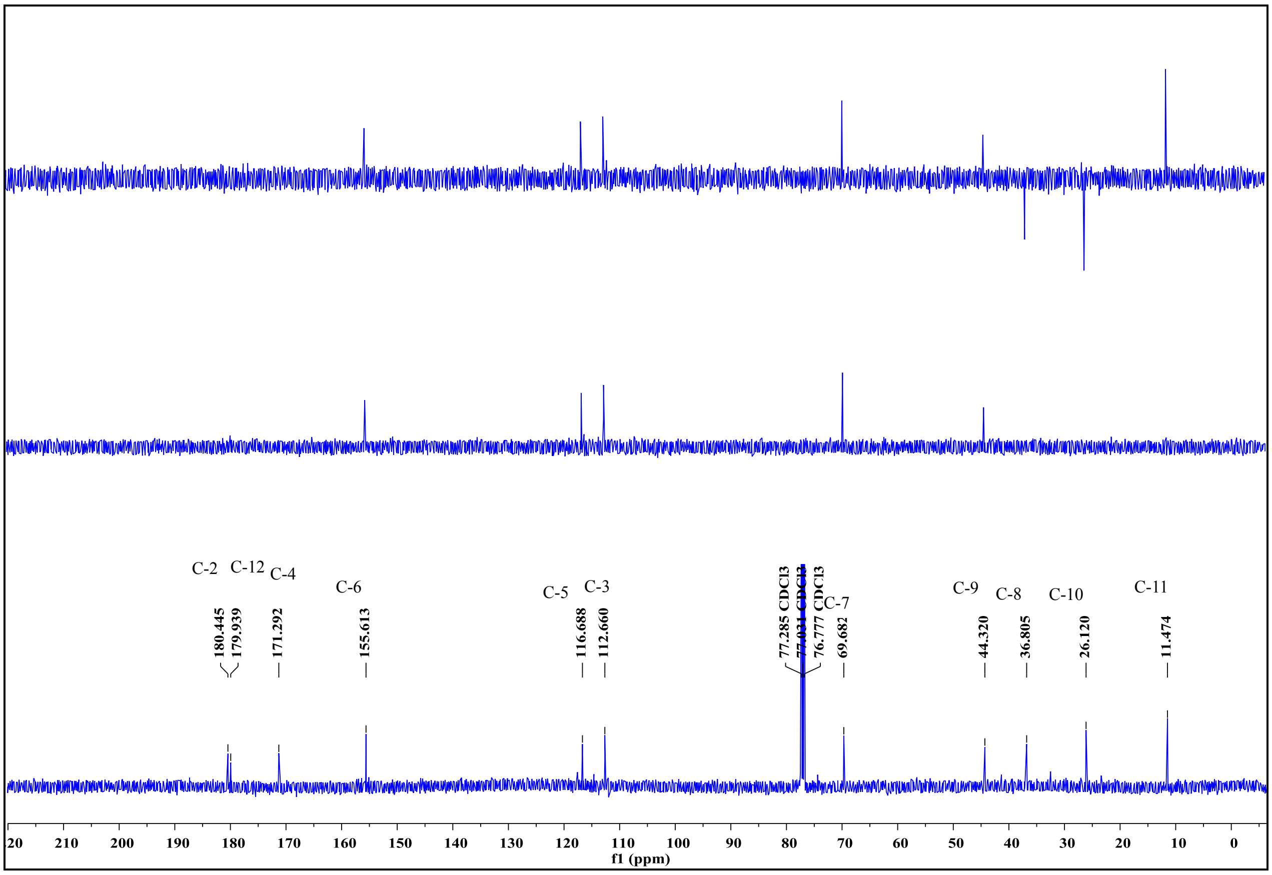

Fig. S14 HSQC spectrum ($CDCl_3$, 500 MHz) of xylariahgin B (**2**)

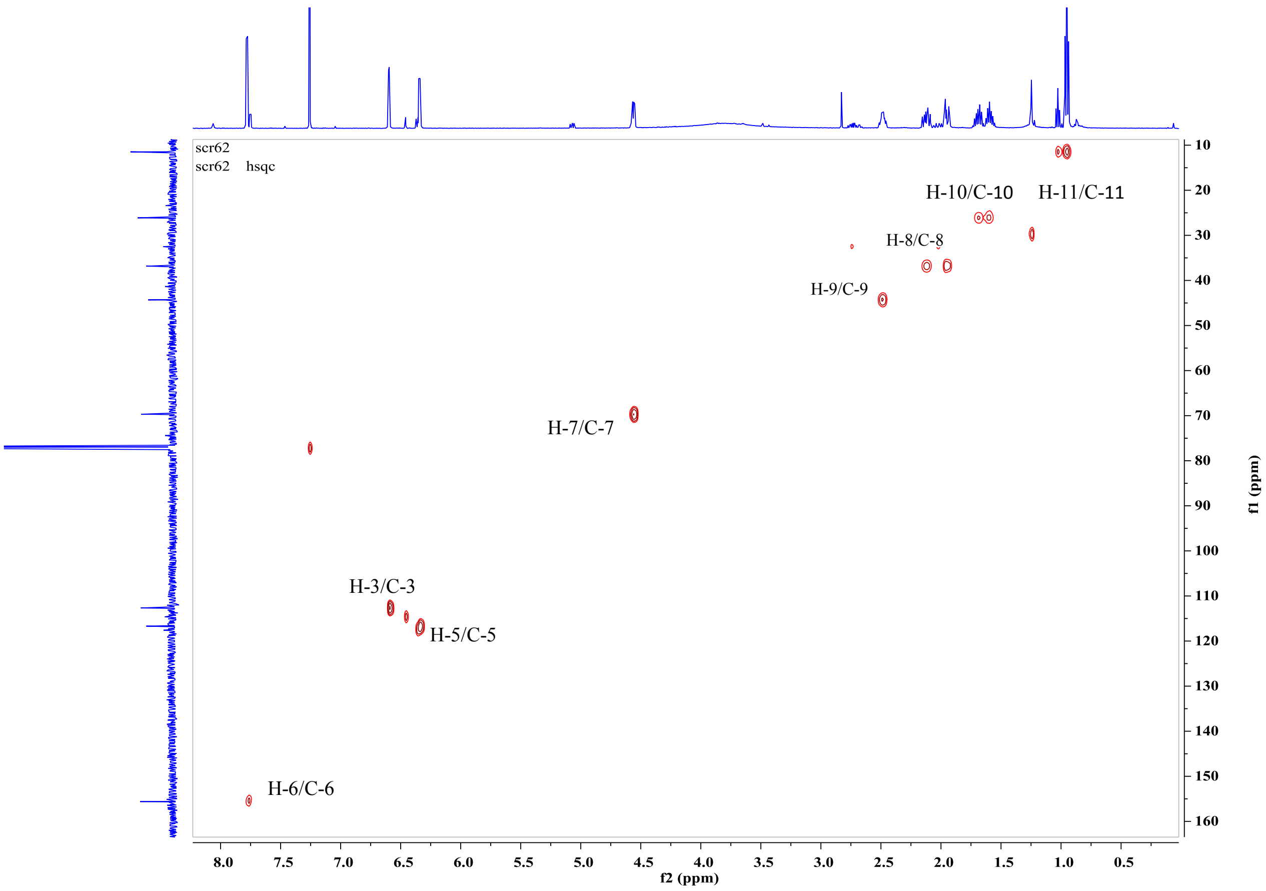

Fig. S15 ^{1}H-^{1}HCOSY spectrum ($CDCl_3$, 500 MHz) of xylariahgin B (**2**)

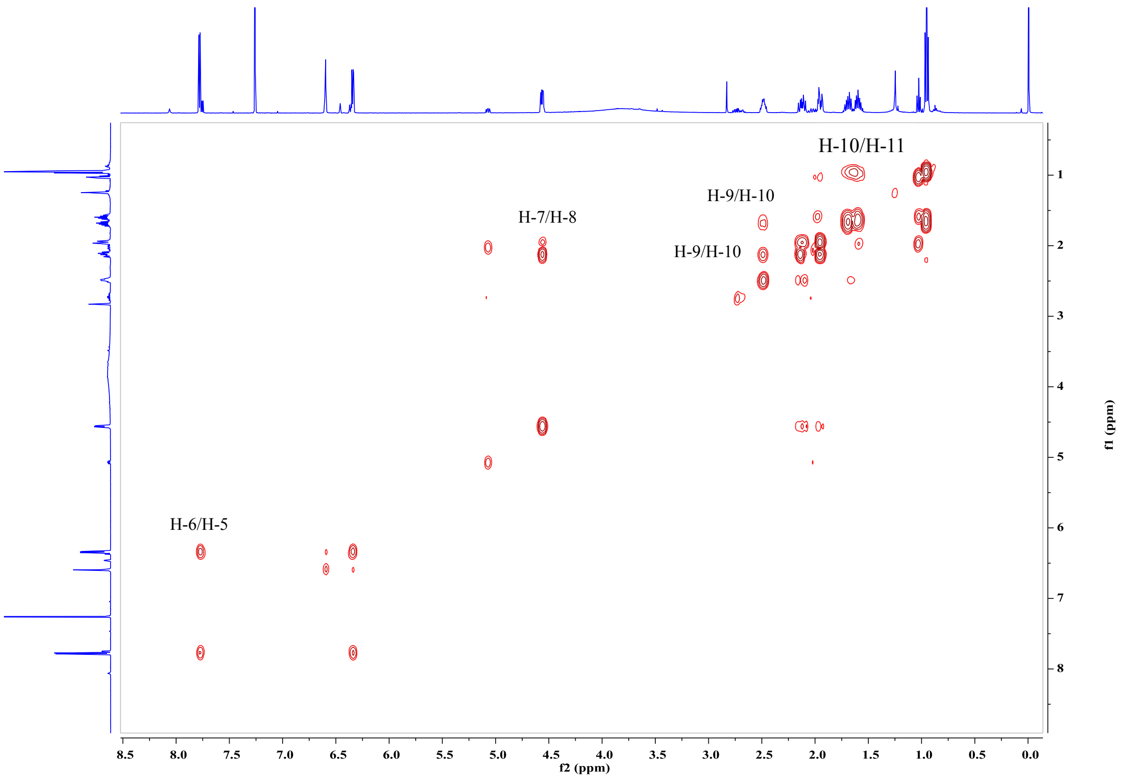

Fig. S16 HMBC spectrum ($CDCl_3$, 500MHz) of xylariahgin B (**2**)

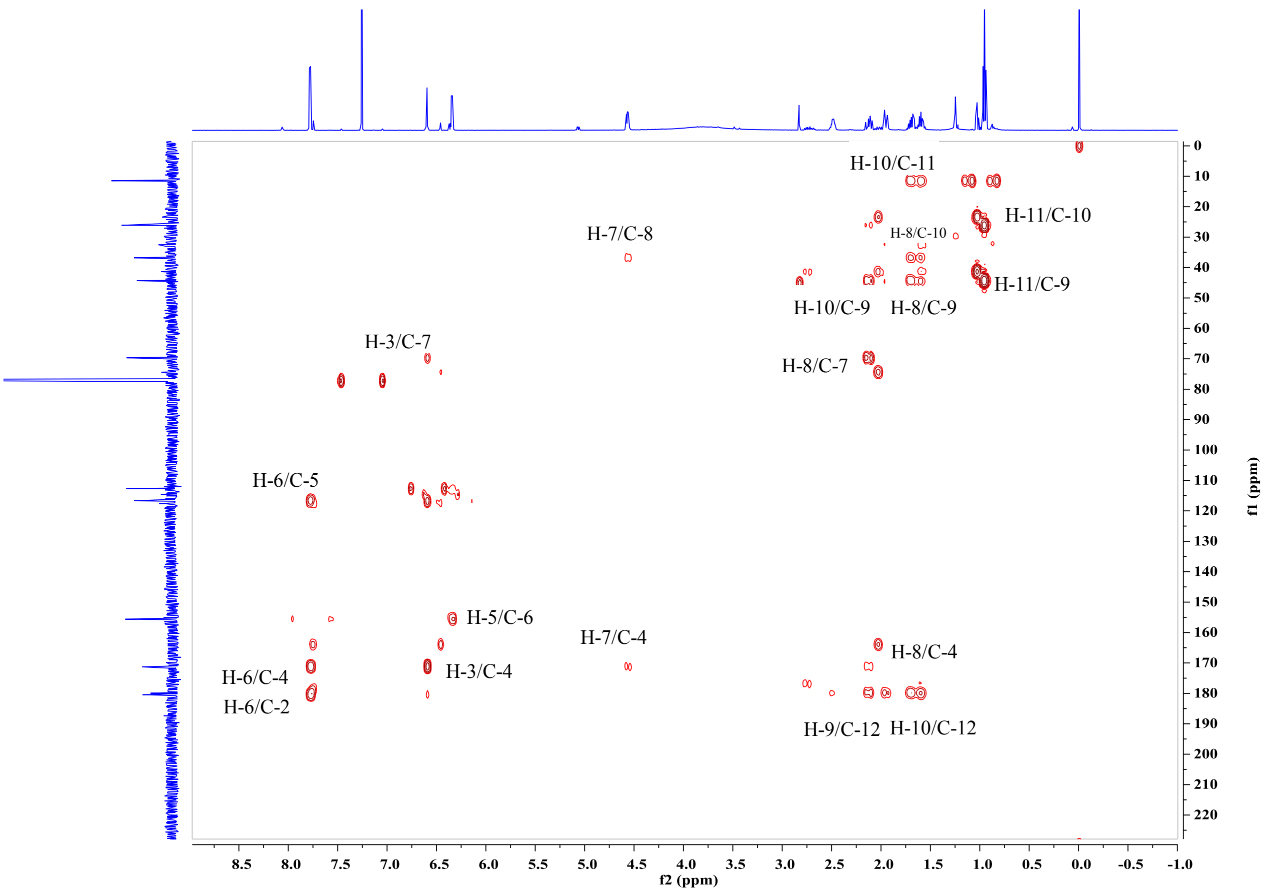

Fig. S17 ROESY spectrum ($CDCl_3$, 500 MHz) of xylariahgin B (**2**)

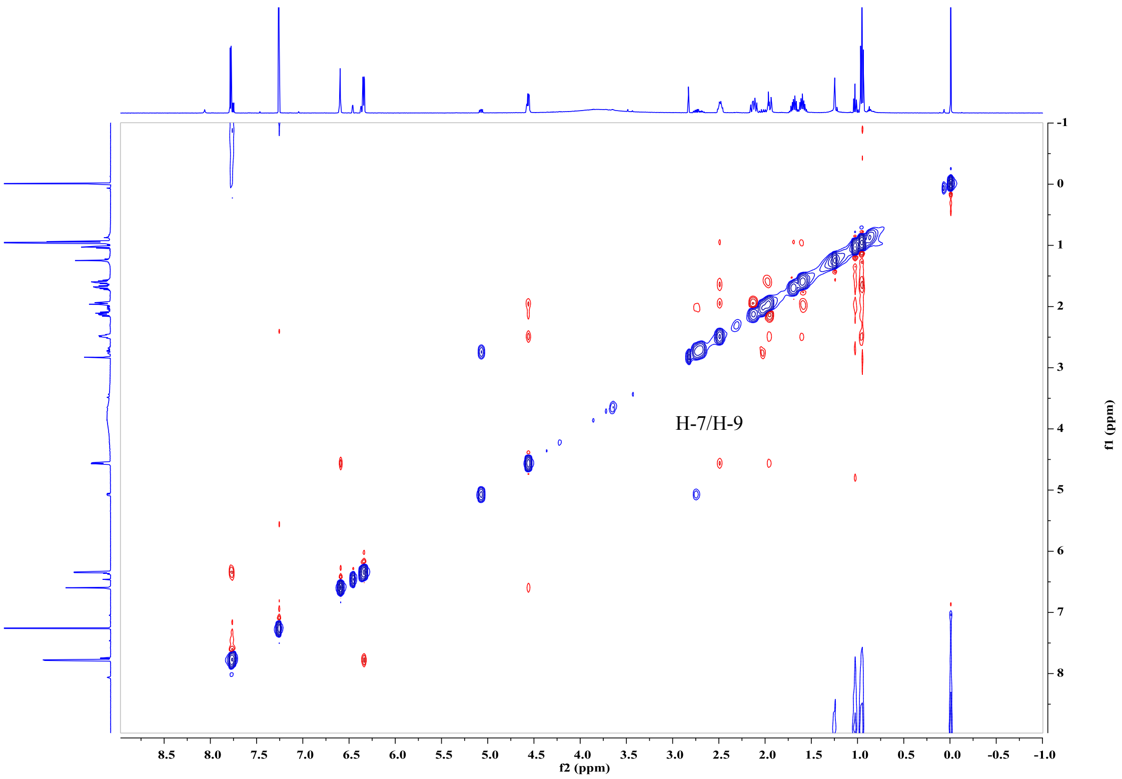

Fig. S18 UV spectrum of xylariahgin B (**2**)

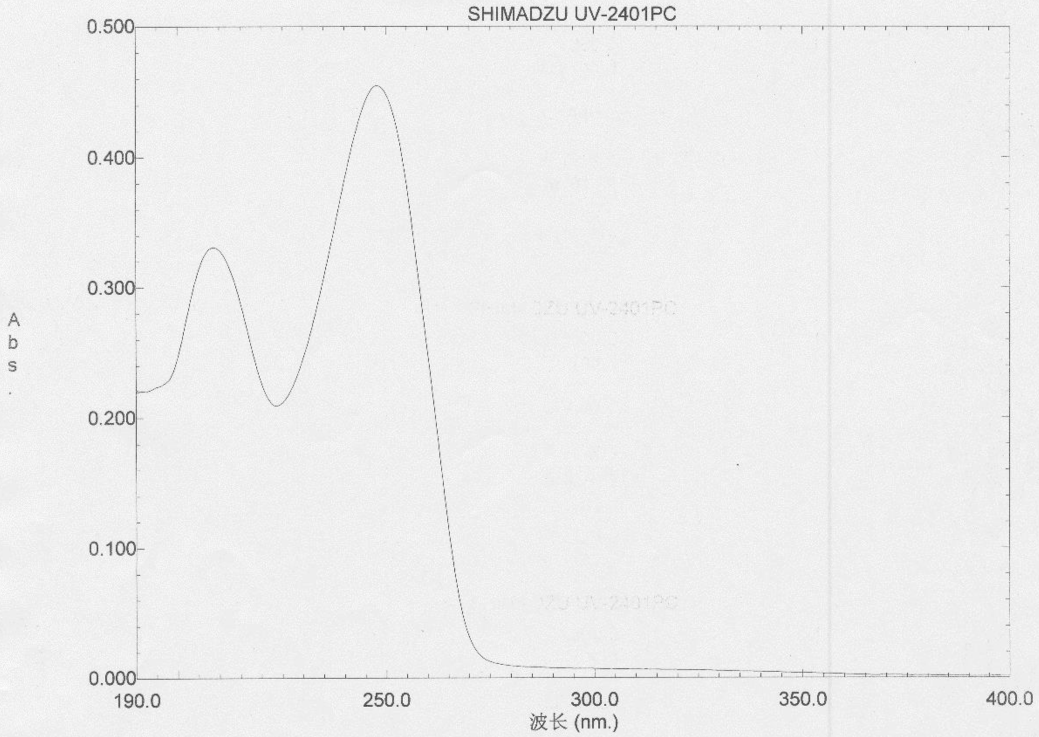

文件名: SCR62

SCR62 ———

样品浓度：0.0106毫克/毫升
溶剂：甲醇

创建于: 18:13 17-09-15
数据: 原始

测量模式: Abs.
扫描速度: 中速
狭缝: 5.0
采样间隔: 0.5

否.	波长 (nm.)	Abs.
1	248.00	0.4546
2	208.50	0.3307

Fig. S19 [α] spectrum of xylariahgin B (**2**)

Optical rotation measurement

Model : P-1020 (A060460638)

No.	Sample	Mode	Data	Monitor<br>Blank	Temp.<br>Cell<br>Temp Point	Date<br>Comment<br>Sample Name	Light<br>Filter<br>Operator	Cycle Time<br>Integ Time	
No.1	12 (1/3)	Sp.Rot	-18.2760	-0.0106<br>0.0000	23.5<br>50.00<br>Cell	Thu Sep 07 12:09:22 2017<br>0.00116g/mL MeOH<br>SCR62	Na<br>589nm	2 sec<br>2 sec	
No.2	12 (2/3)	Sp.Rot	-20.3450	-0.0118<br>0.0000	23.5<br>50.00<br>Cell	Thu Sep 07 12:09:27 2017<br>0.00116g/mL MeOH<br>SCR62	Na<br>589nm	2 sec<br>2 sec	-20.0000°
No.3	12 (3/3)	Sp.Rot	-21.3790	-0.0124<br>0.0000	23.5<br>50.00<br>Cell	Thu Sep 07 12:09:33 2017<br>0.00116g/mL MeOH<br>SCR62	Na<br>589nm	2 sec<br>2 sec	

Fig. S20 IR spectrum of xylariahgin B (**2**)

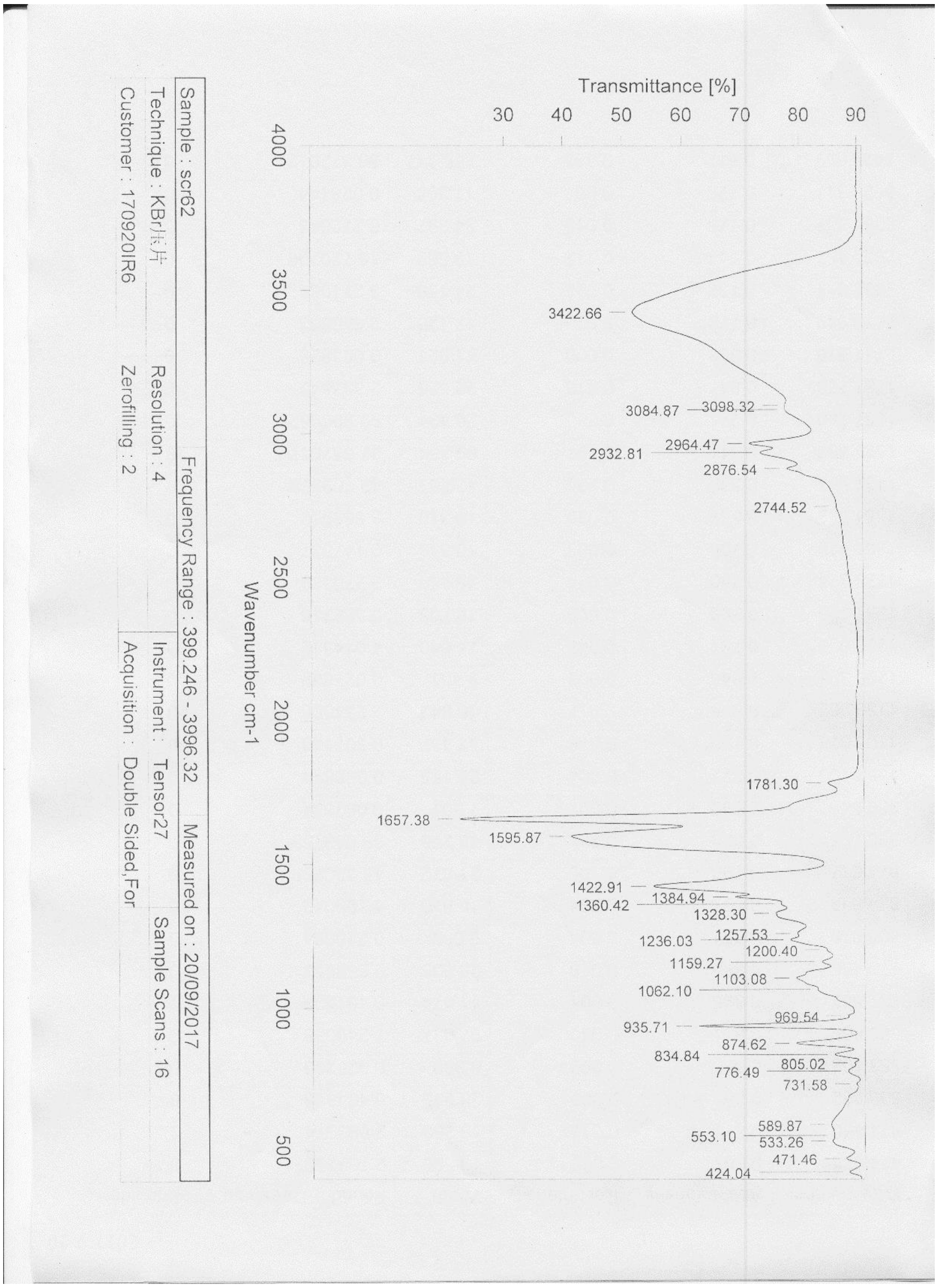

Fig. S21 HRESIMS spectrum of xylariahgin C (**3**)

Formula Predictor Report - scr61a.lcd

Page 1 of 1

Data File: E:\DATA\2017\0904\scr61a.lcd

Elmt	Val.	Min	Max	Elmt	Val.	Min	Max	Elmt	Val.	Min	Max	Elmt	Val.	Min	Max	Use Adduct
H	1	0	150	O	2	0	50	P	3	0	0	Br	1	0	0	Na
B	3	0	0	F	1	0	0	S	2	0	0	I	3	0	0	
C	4	0	50	Na	1	0	0	Cl	1	0	0	Pt	2	0	0	
N	3	0	0	Mg	2	0	0	Fe	2	0	0					

Error Margin (ppm): 5
HC Ratio: unlimited
Max Isotopes: all
MSn Iso RI (%): 75.00

DBE Range: -2.0 - 100.0
Apply N Rule: yes
Isotope RI (%): 1.00
MSn Logic Mode: AND

Electron Ions: both
Use MSn Info: yes
Isotope Res: 10000
Max Results: 10

Event#: 1 MS(E+) Ret. Time : 0.470 -> 0.480 Scan# : 95 -> 97

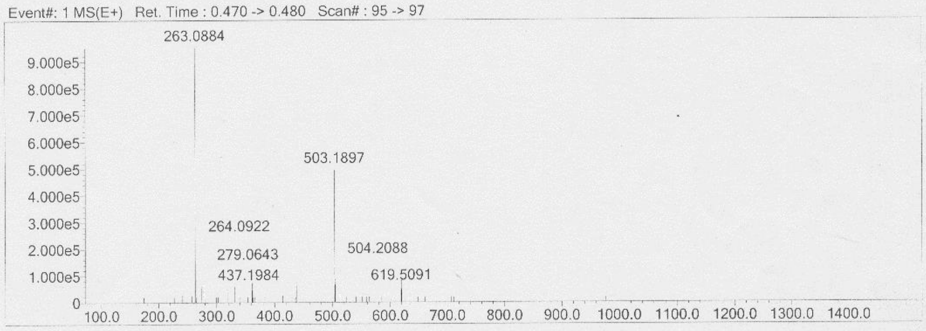

Measured region for 263.0884 m/z

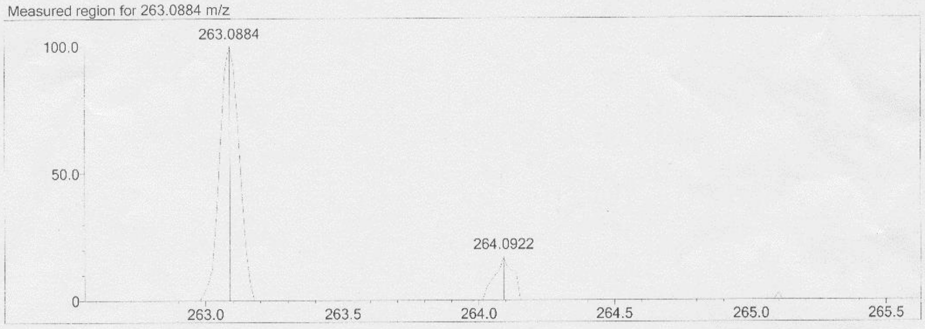

C12 H16 O5 [M+Na]+ : Predicted region for 263.0890 m/z

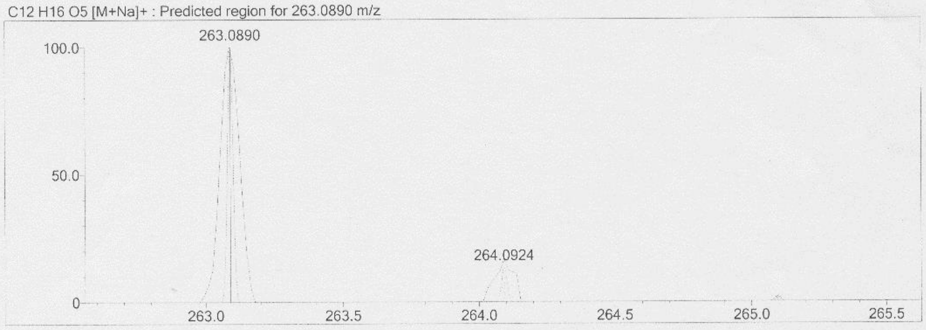

Formula (M)	Ion	Meas. m/z	Pred. m/z	Df. (mDa)	Df. (ppm)	DBE
C12 H16 O5	[M+Na]+	263.0884	263.0890	-0.6	-2.28	5.0

Fig. S22 ^{1}H NMR spectrum (Acetone-d_6, 600 MHz) of xylariahgin C (3)

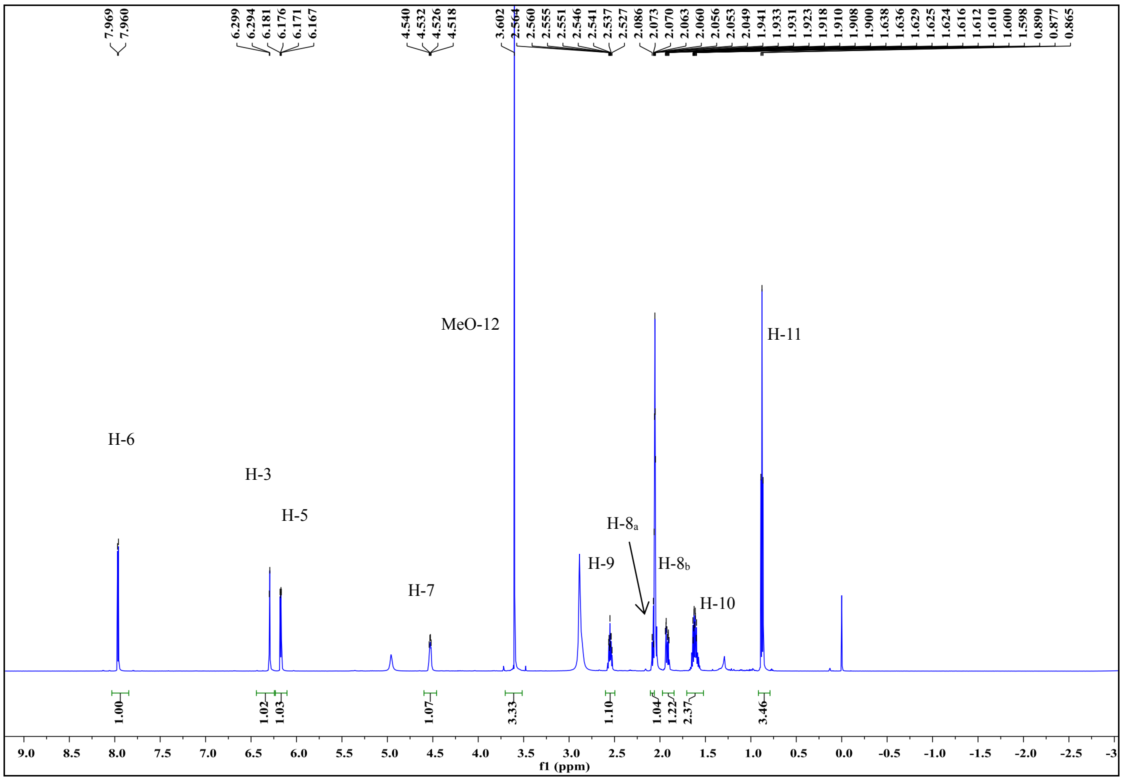

Fig. S23 ^{13}C NMR spectrum (Acetone-d_6, 600 MHz) of xylariahgin C (**3**)

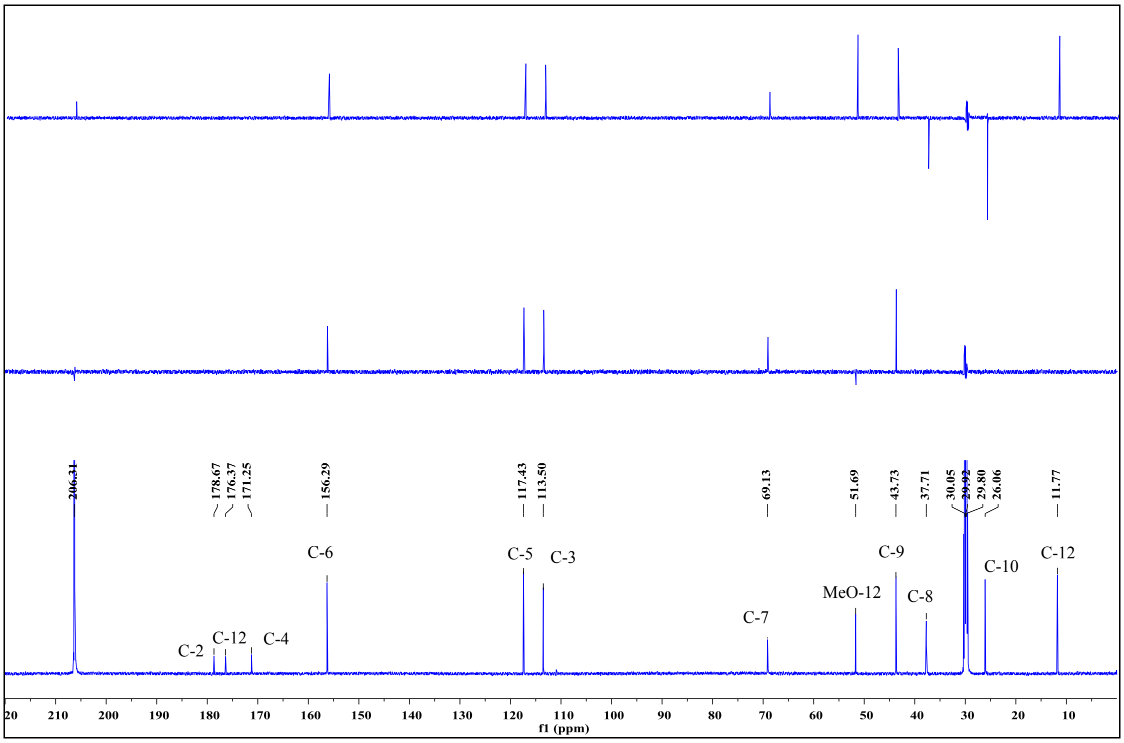

Fig. S24 HSQC spectrum (Acetone-d_6, 600 MHz) of xylariahgin C (**3**)

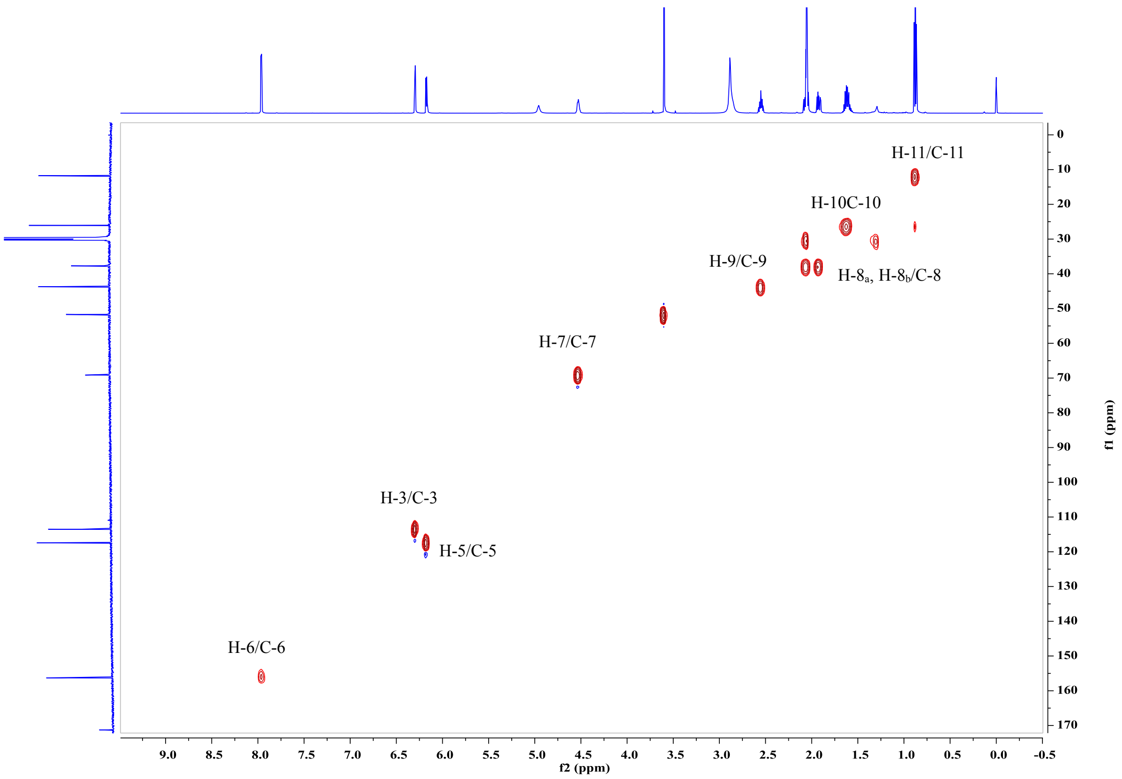

Fig. S25 ^{1}H-^{1}H COSY spectrum (Acetone-d_6, 600 MHz) of xylariahgin C (**3**)

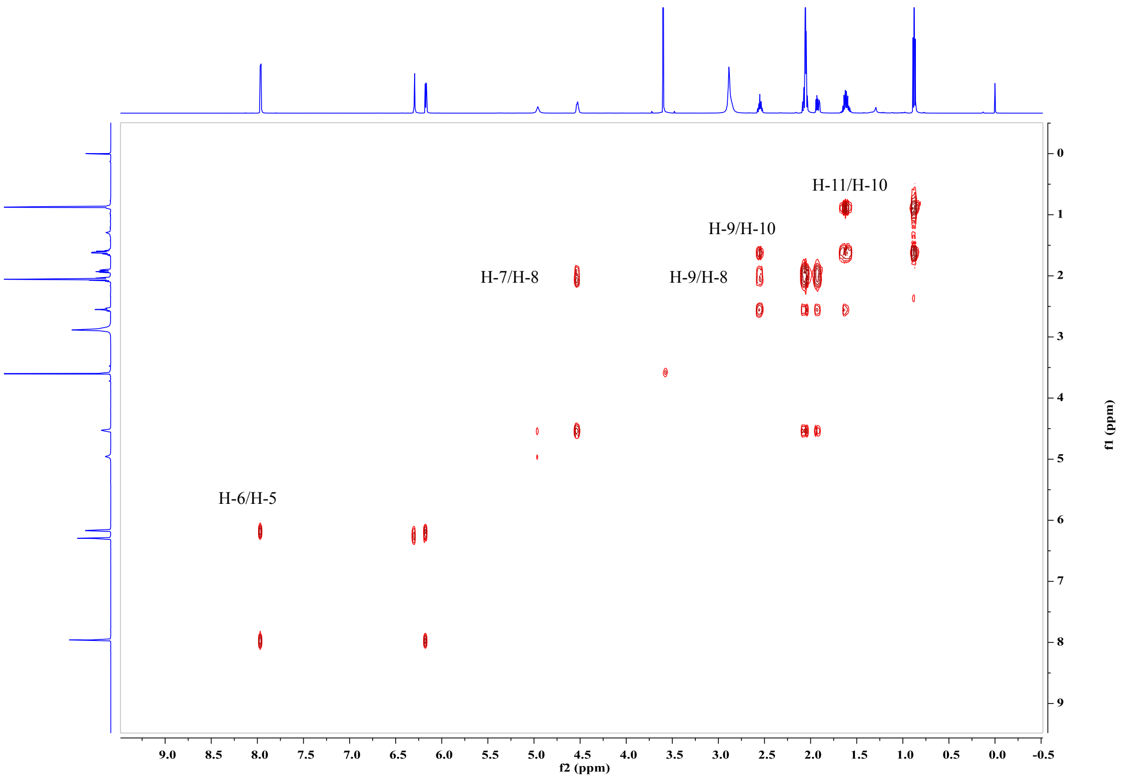

Fig. S26 HMBC spectrum (Acetone-d_6, 600 MHz) of xylariahgin C (**3**)

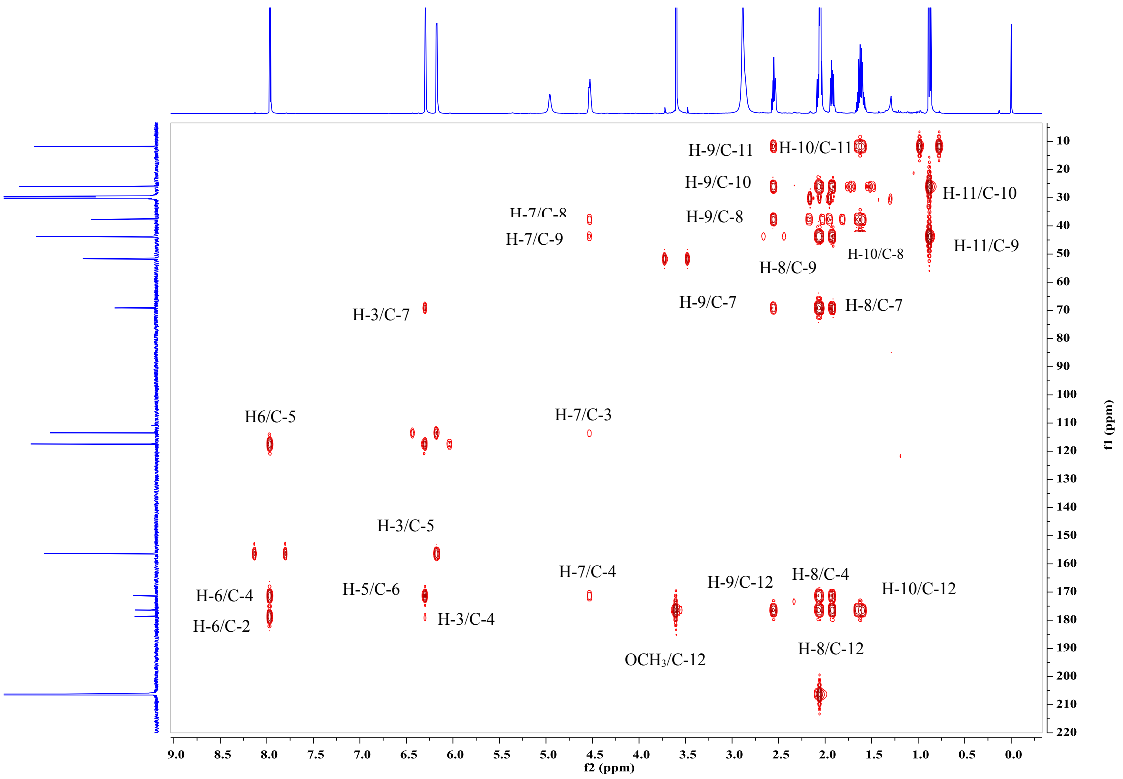

Fig. S27 ROESY spectrum (Acetone-d_6, 600 MHz) of xylariahgin C (**3**)

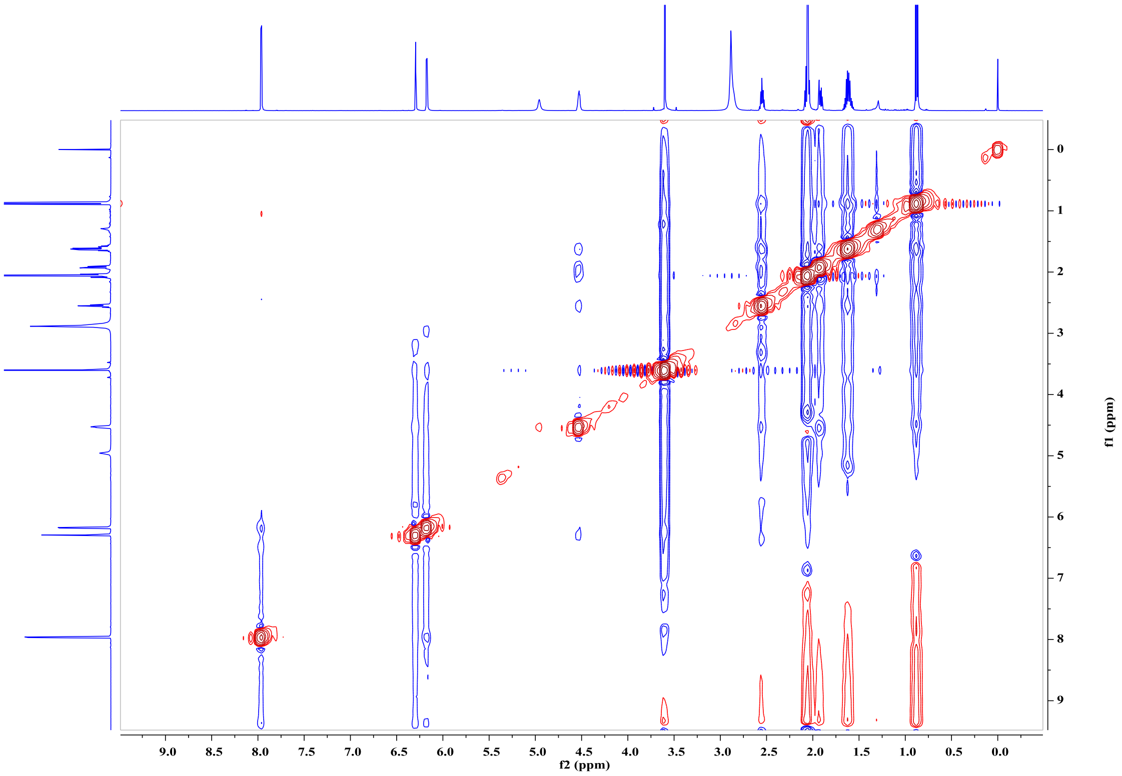

Fig. S28 UV spectrum of xylariahgin C (**3**)

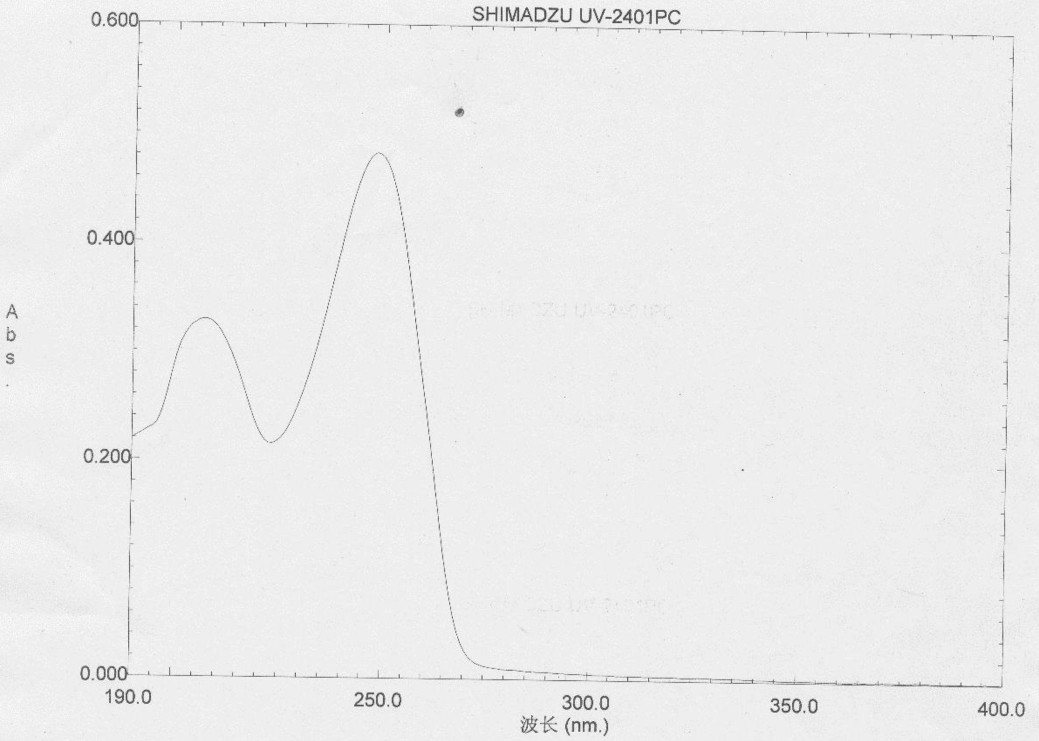

文件名: SCR61A

SCR61A

样品浓度：0.0081毫克/毫升
溶剂：甲醇

创建于: 14:32 17-09-05
数据: 原始

测量模式: Abs.
扫描速度: 中速
狭缝: 5.0
采样间隔: 0.5

否.	波长 (nm.)	Abs.
1	248.00	0.4821
2	207.00	0.3287

Fig. S29 [α] spectrum of xylariahgin C (**3**)

Optical rotation measurement

Model : P-1020 (A060460638)

No.	Sample	Mode	Data	Monitor<br>Blank	Temp.<br>Cell<br>Temp Point	Date<br>Comment<br>Sample Name	Light<br>Filter<br>Operator	Cycle Time<br>Integ Time
No.1	6 (1/3)	Sp.Rot	-23.0770	-0.0024<br>0.0000	23.7<br>10.00<br>Cell	Fri Sep 15 10:39:21 2017<br>0.00104g/mL MeOH<br>SCR61A	Na<br>589nm	2 sec<br>2 sec
No.2	6 (2/3)	Sp.Rot	-22.1150	-0.0023<br>0.0000	23.7<br>10.00<br>Cell	Fri Sep 15 10:39:27 2017<br>0.00104g/mL MeOH<br>SCR61A	Na<br>589nm	2 sec<br>2 sec
No.3	6 (3/3)	Sp.Rot	-24.0380	-0.0025<br>0.0000	23.7<br>10.00<br>Cell	Fri Sep 15 10:39:32 2017<br>0.00104g/mL MeOH<br>SCR61A	Na<br>589nm	2 sec<br>2 sec

-23.0769°

Fig. S30 IR spectrum of xylariahgin C (**3**)

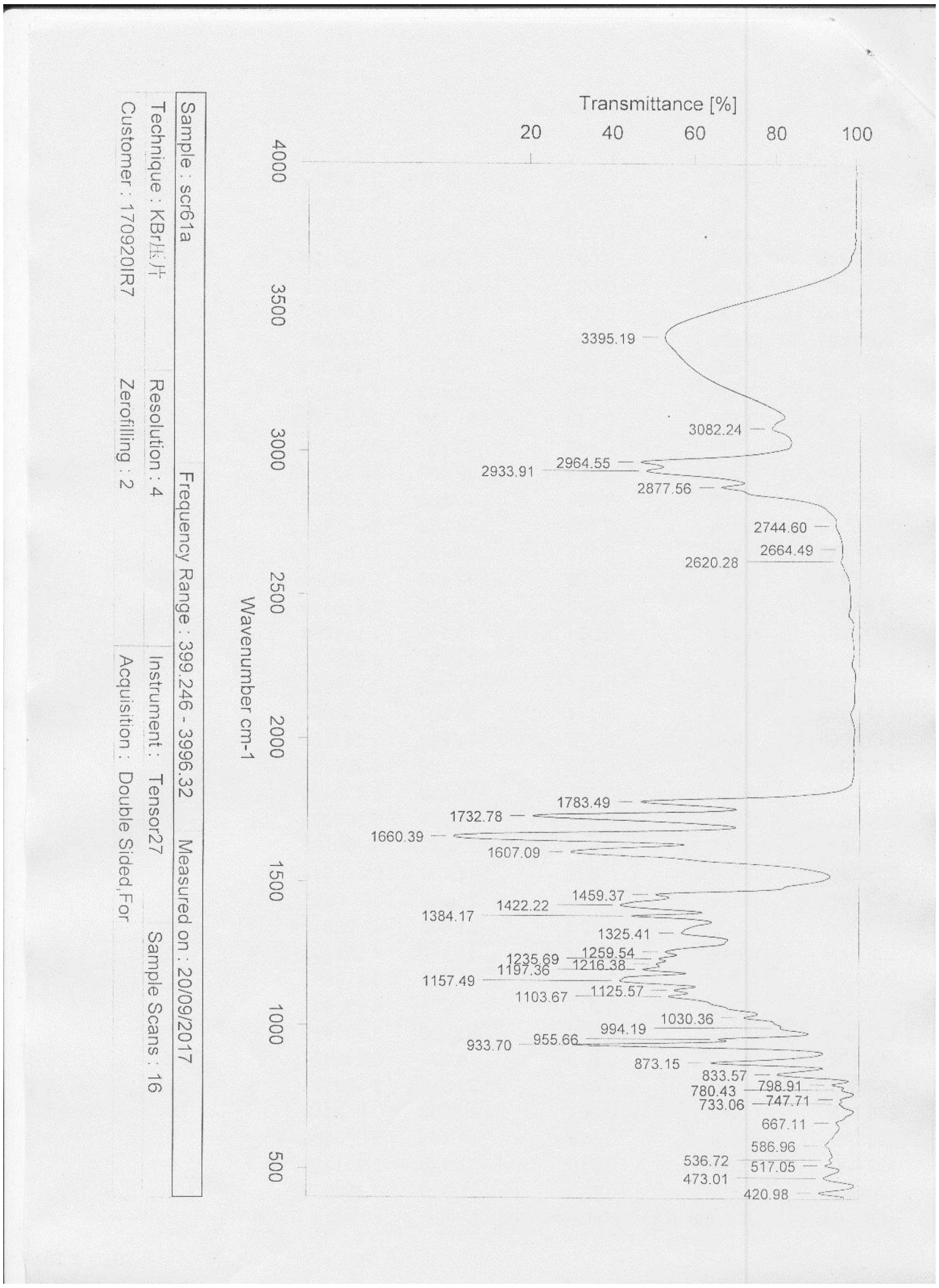

Fig. S31 HPLC profiles of xylariahgins A-C (**1**-**3**)

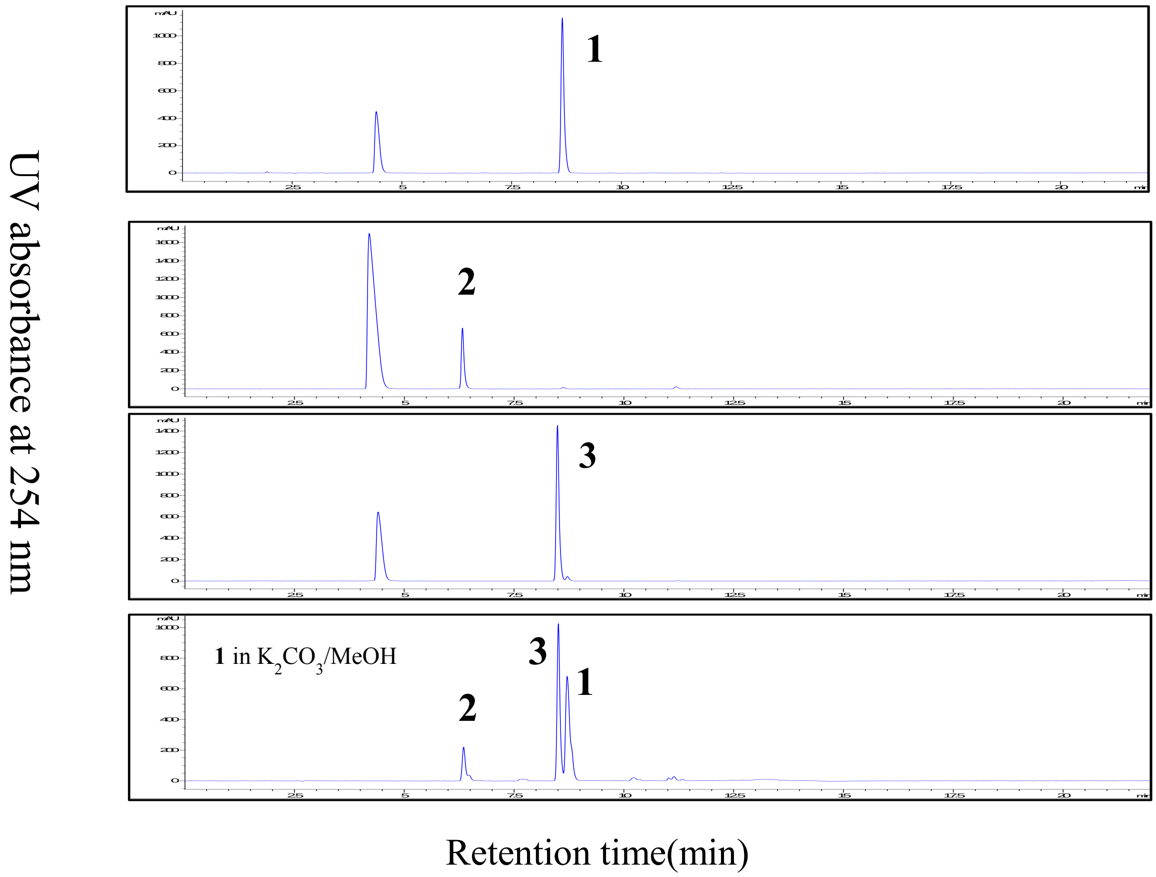

Fig. S32 HRESIMS spectrum of xylariahgin D (**4**)

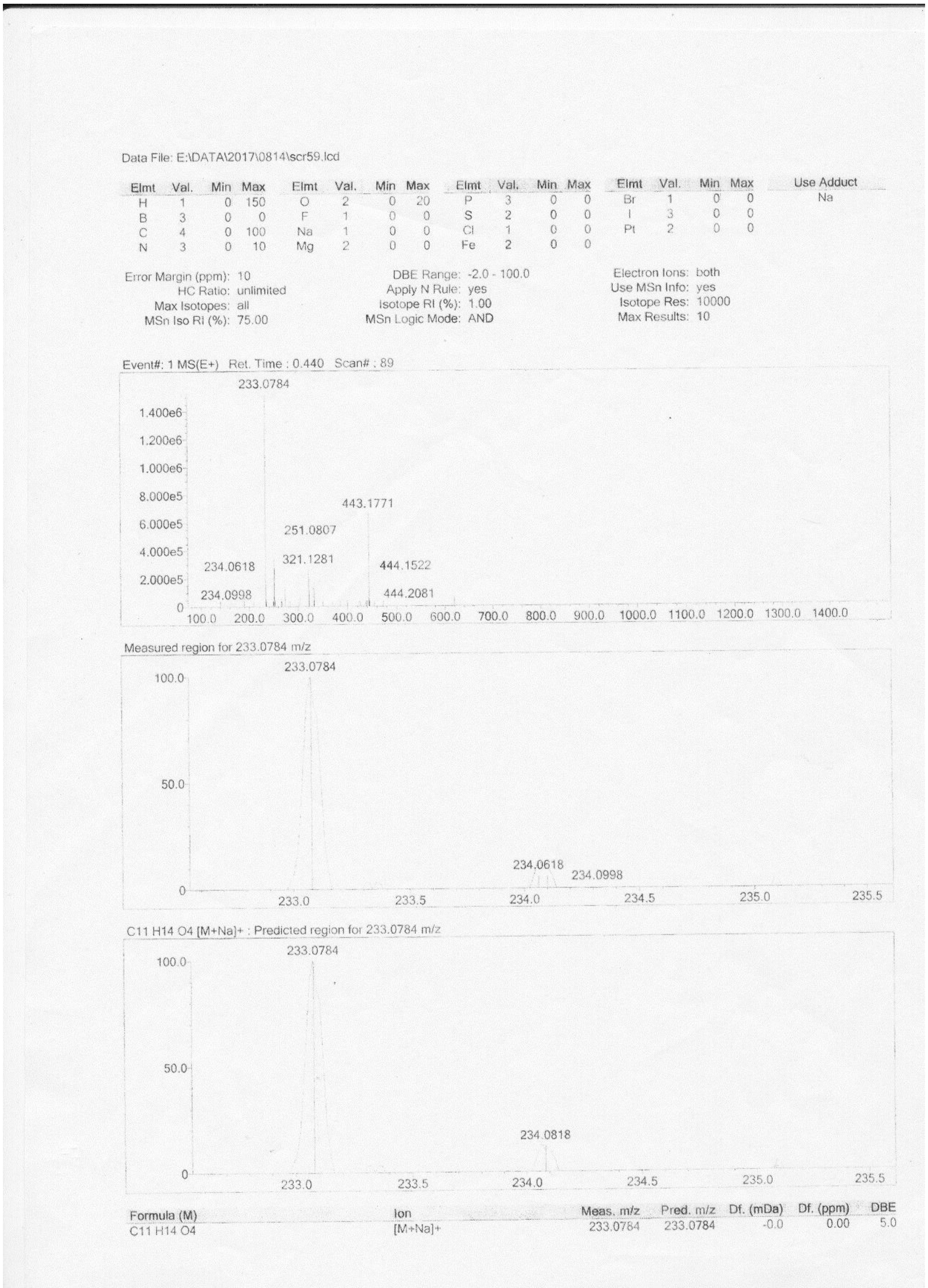

Data File: E:\DATA\2017\0814\scr59.lcd

Elmt	Val.	Min	Max	Elmt	Val.	Min	Max	Elmt	Val.	Min	Max	Elmt	Val.	Min	Max	Use Adduct
H	1	0	150	O	2	0	20	P	3	0	0	Br	1	0	0	Na
B	3	0	0	F	1	0	0	S	2	0	0	I	3	0	0	
C	4	0	100	Na	1	0	0	Cl	1	0	0	Pt	2	0	0	
N	3	0	10	Mg	2	0	0	Fe	2	0	0					

Error Margin (ppm): 10
HC Ratio: unlimited
Max Isotopes: all
MSn Iso RI (%): 75.00

DBE Range: -2.0 - 100.0
Apply N Rule: yes
Isotope RI (%): 1.00
MSn Logic Mode: AND

Electron Ions: both
Use MSn Info: yes
Isotope Res: 10000
Max Results: 10

Formula (M)	Ion	Meas. m/z	Pred. m/z	Df. (mDa)	Df. (ppm)	DBE
C11 H14 O4	[M+Na]+	233.0784	233.0784	-0.0	0.00	5.0

Fig. S33 ^{1}H NMR spectrum ($CDCl_3$, 500 MHz) of xylariahgin D (**4**)

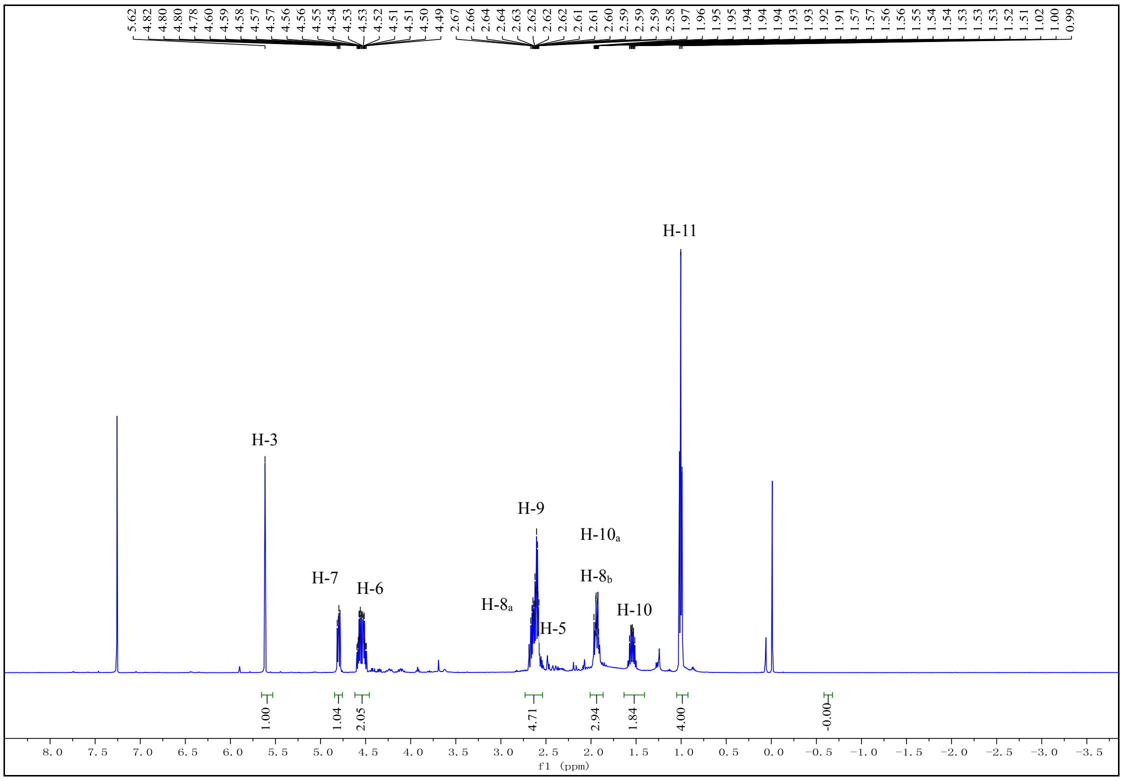

Fig. S34 ^{13}C NMR spectrum ($CDCl_3$, 500 MHz) of xylariahgin D (**4**)

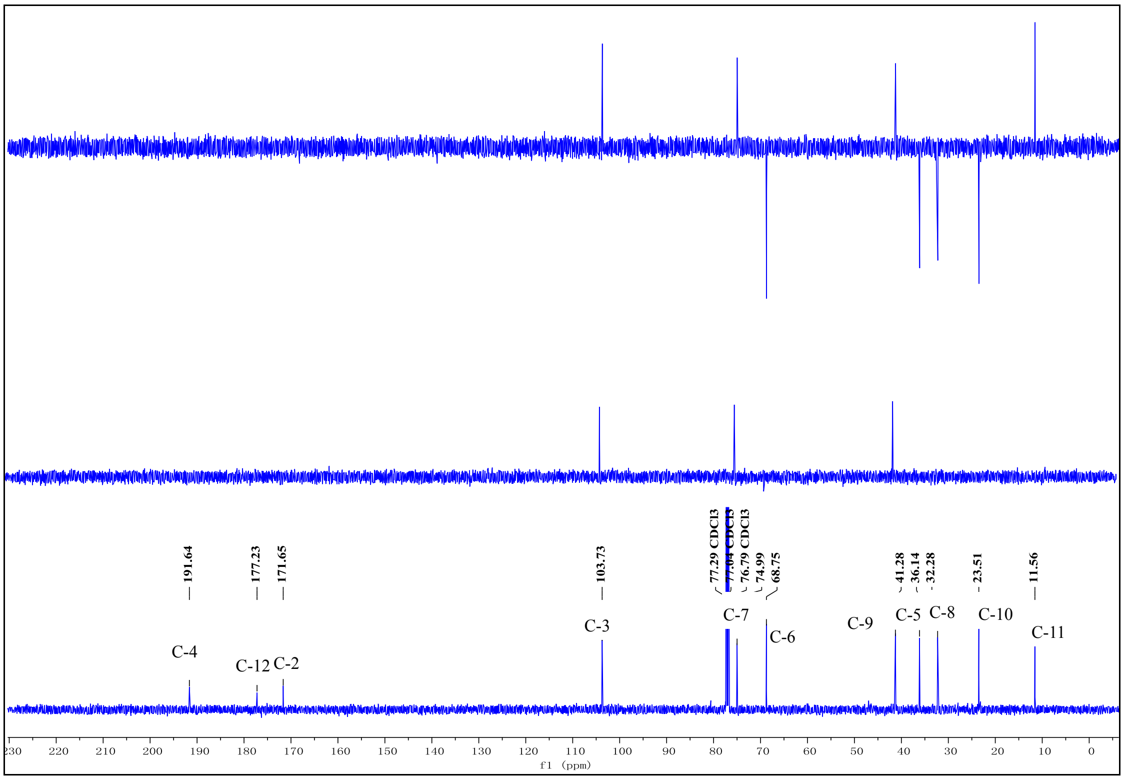

Fig. S35 HSQC spectrum ($CDCl_3$, 500 MHz) of xylariahgin D (**4**)

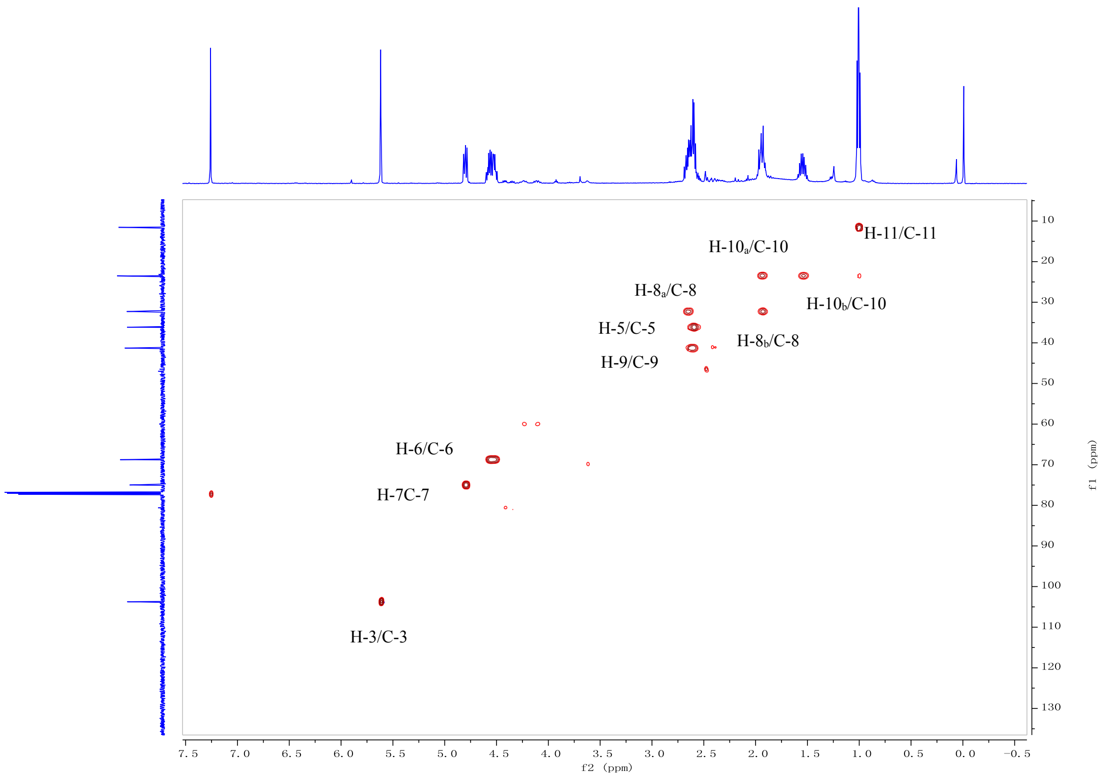

Fig. S36 ^{1}H-^{1}H COSY spectrum ($CDCl_3$, 500 MHz) of xylariahgin D (**4**)

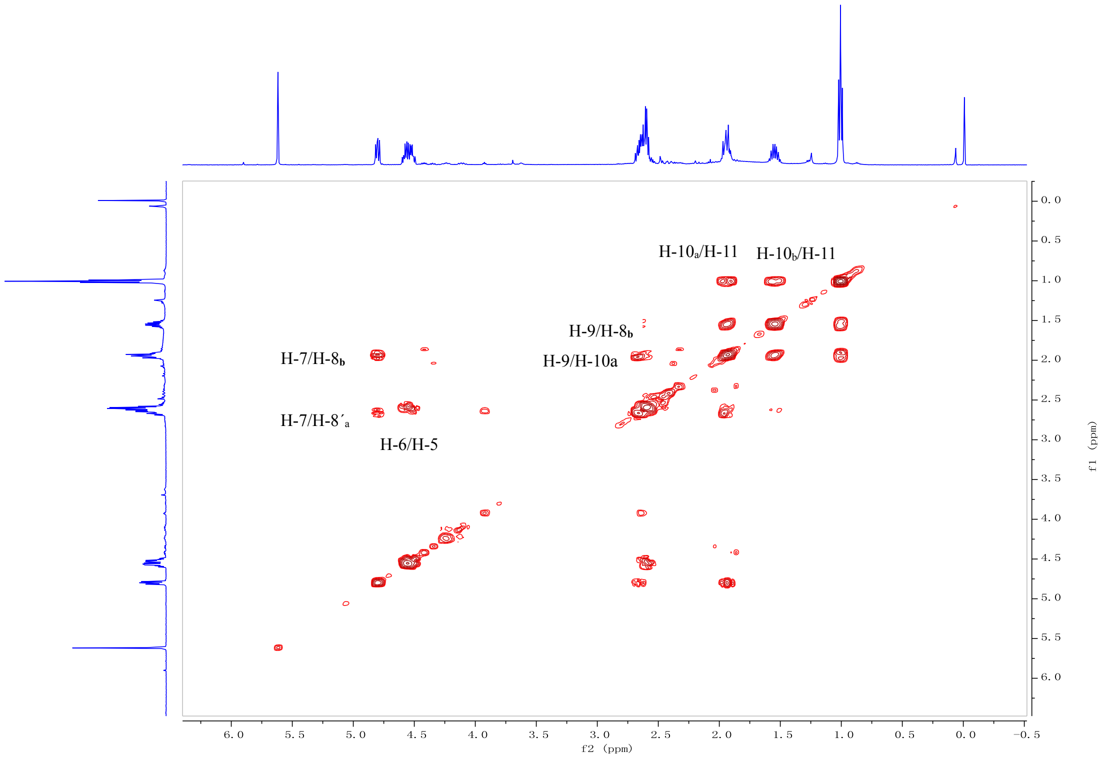

Fig. S37 HMBC spectrum ($CDCl_3$, 500 MHz) of xylariahgin D (**4**)

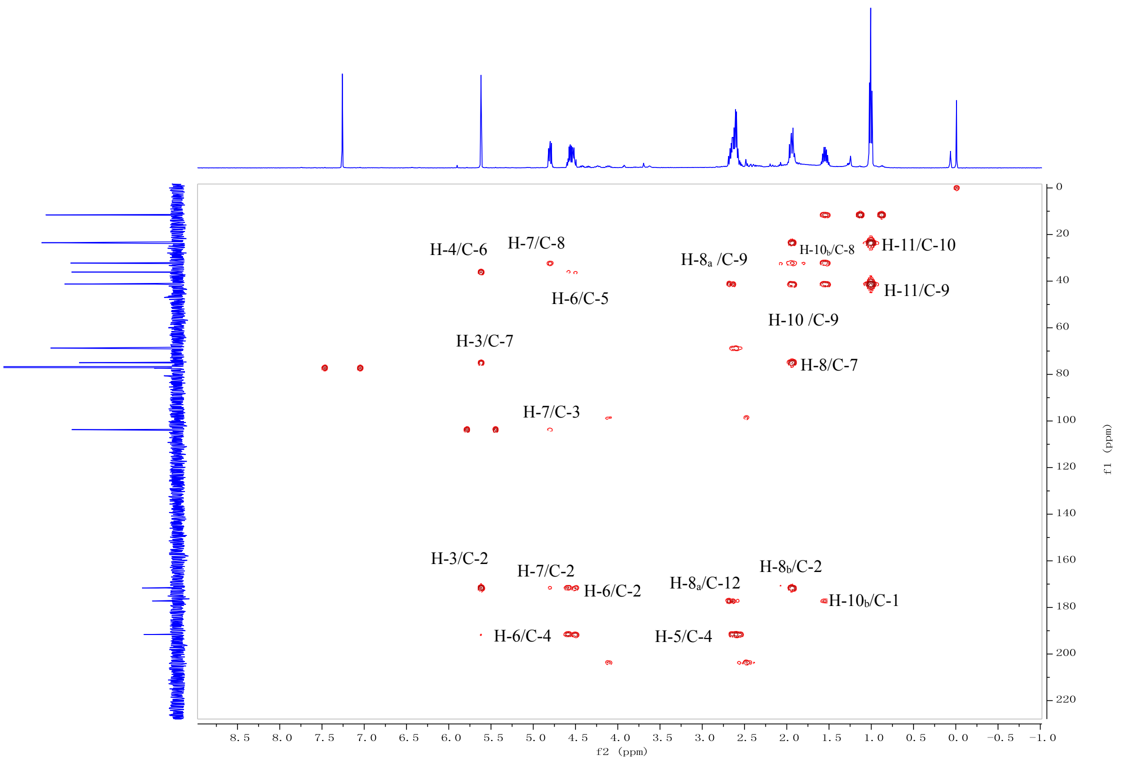

Fig. S38 ROESY spectrum ($CDCl_3$, 500 MHz) of xylariahgin D (**4**)

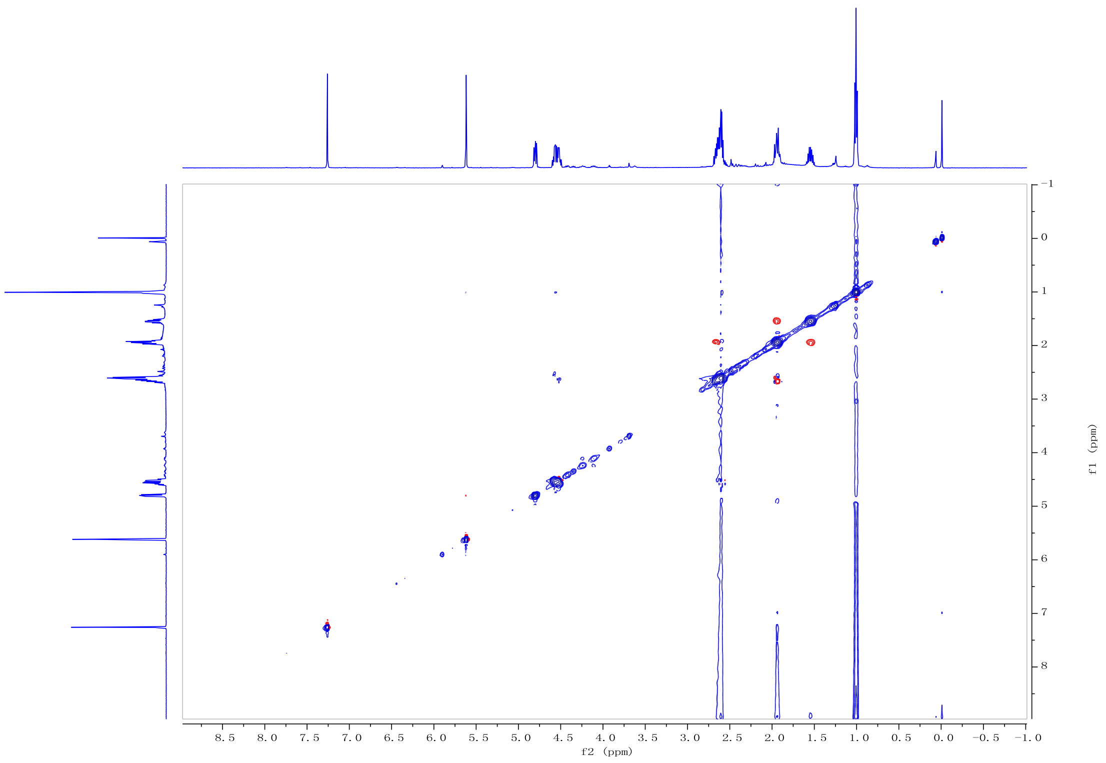

Fig. S39 UV spectrum of xylariahgin D (**4**)

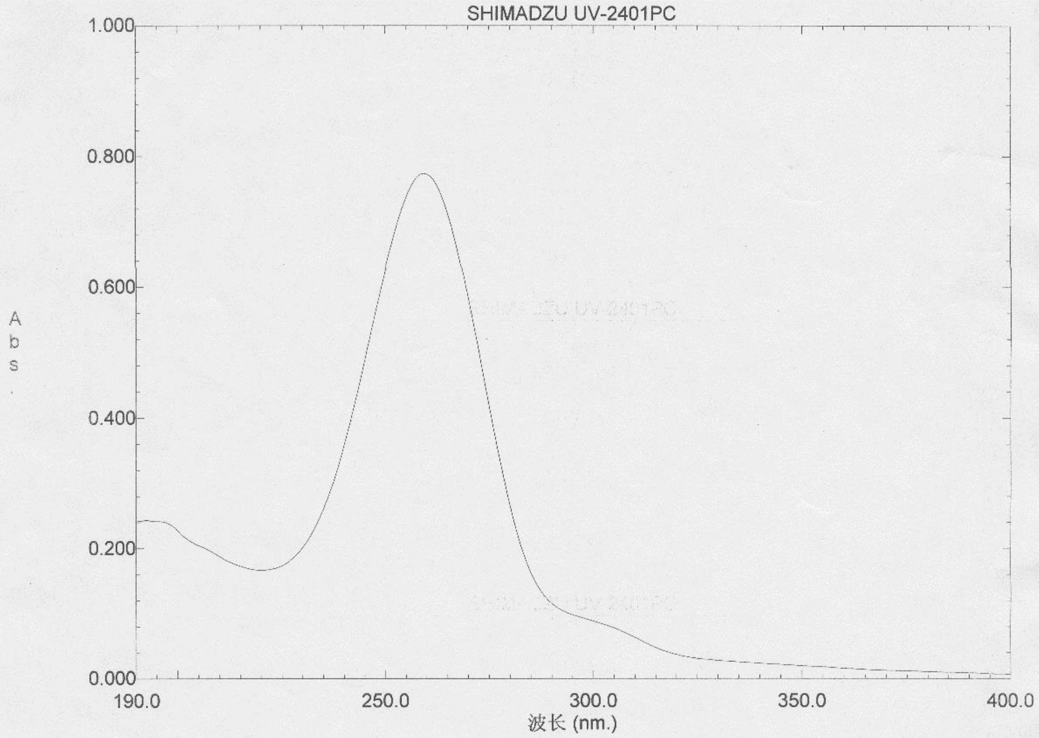

文件名: SCR52

SCR52 —— [illegible]

样品浓度：0.0171毫克/毫升
溶剂：甲醇

创建于: 17:13 17-08-17
数据: 原始

测量模式: Abs.
扫描速度: 中速
狭缝: 5.0
采样间隔: 0.5

否.	波长 (nm.)	Abs.
1	195.50	0.2416
2	260.00	0.7728
3	295.50	0.0967

Fig. S40 [α] spectrum of xylariahgin D (**4**)

Optical rotation measurement

Model : P-1020 (A060460638)

No.	Sample	Mode	Data	Monitor<br>Blank	Temp.<br>Cell<br>Temp Point	Date<br>Comment<br>Sample Name	Light<br>Filter<br>Operator	Cycle Time<br>Integ Time
No.1	32 (1/3)	Sp.Rot	-41.9610	-0.0214<br>0.0000	23.7<br>50.00<br>Cell	Sat Jul 22 04:58:12 2017<br>0.00102g/mL MeOH<br>SCR59	Na<br>589nm	2 sec<br>2 sec
No.2	32 (2/3)	Sp.Rot	-45.8820	-0.0234<br>0.0000	23.7<br>50.00<br>Cell	Sat Jul 22 04:58:17 2017<br>0.00102g/mL MeOH<br>SCR59	Na<br>589nm	2 sec<br>2 sec
No.3	32 (3/3)	Sp.Rot	-48.6270	-0.0248<br>0.0000	23.7<br>50.00<br>Cell	Sat Jul 22 04:58:23 2017<br>0.00102g/mL MeOH<br>SCR59	Na<br>589nm	2 sec<br>2 sec
No.4	33 (1/3)	Sp.Rot	-42.7450	-0.0218<br>0.0000	23.8<br>50.00<br>Cell	Sat Jul 22 04:59:09 2017<br>0.00102g/mL MeOH<br>SCR59	Na<br>589nm	2 sec<br>2 sec
No.5	33 (2/3)	Sp.Rot	-49.8040	-0.0254<br>0.0000	23.8<br>50.00<br>Cell	Sat Jul 22 04:59:15 2017<br>0.00102g/mL MeOH<br>SCR59	Na<br>589nm	2 sec<br>2 sec
No.6	33 (3/3)	Sp.Rot	-40.1960	-0.0205<br>0.0000	23.8<br>50.00<br>Cell	Sat Jul 22 04:59:20 2017<br>0.00102g/mL MeOH<br>SCR59	Na<br>589nm	2 sec<br>2 sec

-44.8693°

Fig. S41 IR spectrum of xylariahgin D (**4**)

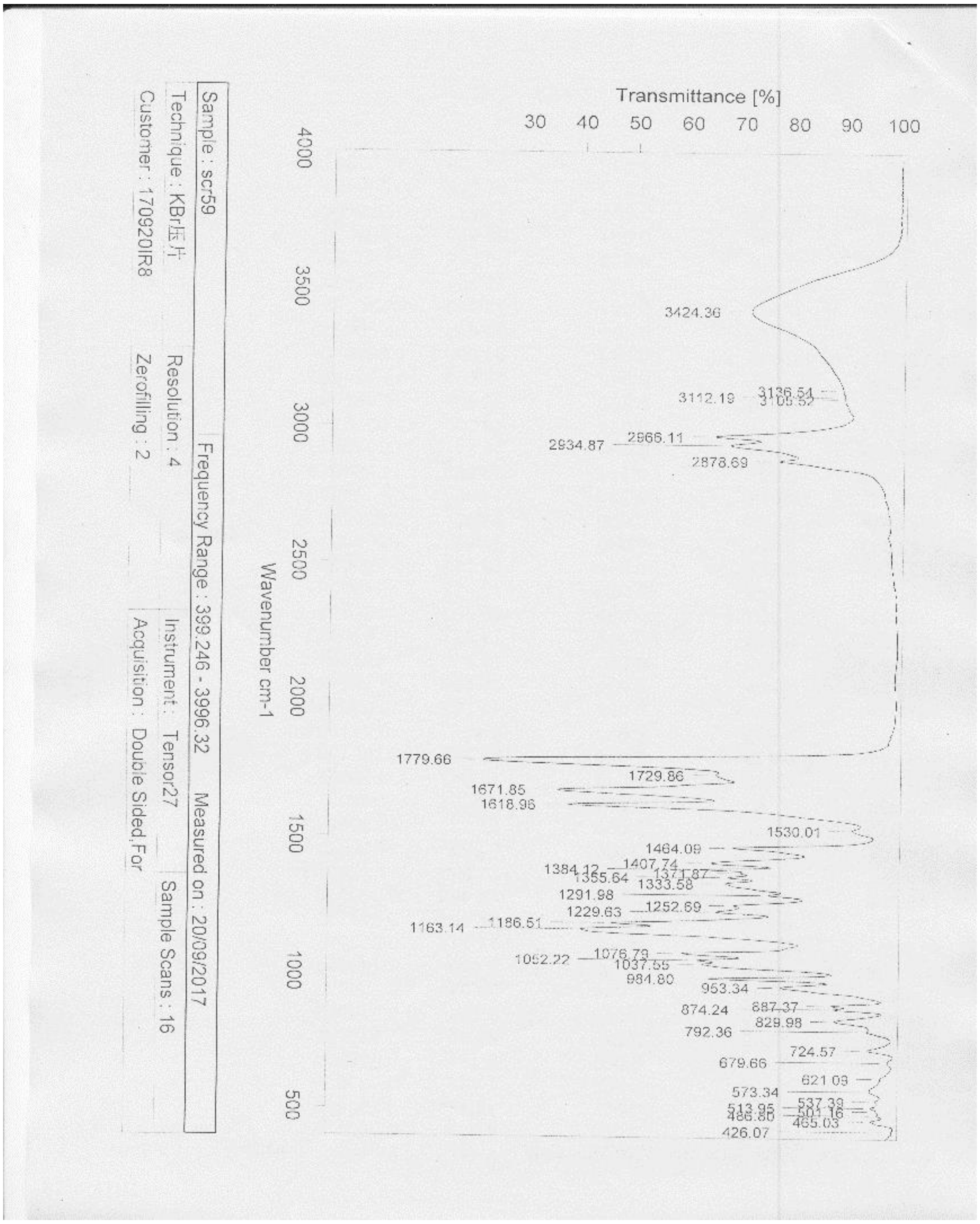

Fig. S42 HRESIMS spectrum of xylariahgin E (**5**)

Qualitative Analysis Report

**Data Filename**	scr56b.d	**Sample Name**	scr56b
**Sample Type**	Sample	**Position**	P1-B1
**Instrument Name**	Instrument 1	**User Name**	
**Acq Method**	SIBU-ESI-i.m	**Acquired Time**	3/1/2018 10:00:32 AM
**IRM Calibration Status**	Success	**DA Method**	Default.m
**Comment**			

Sample Group **Info.**
Acquisition SW 6200 series TOF/6500 series
Version Q-TOF B.05.01 (B5125.2)

User Spectra

Fragmentor Voltage	Collision Energy	Ionization Mode
175	0	ESI

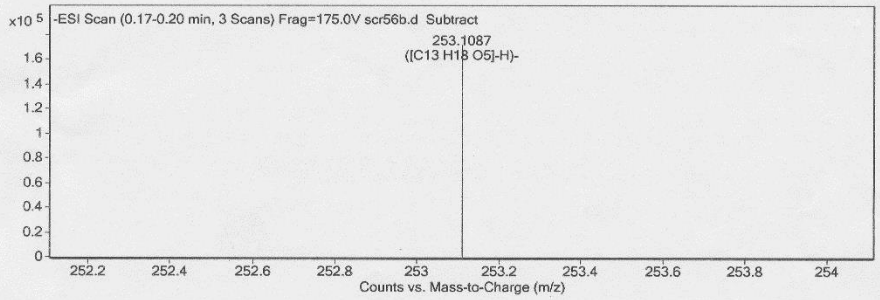

Peak List

m/z	z	Abund	Formula	Ion
149.0971	1	28079.56		
181.1235	1	36699.37		
209.1184	1	16979.71		
241.1449	1	16158.39		
253.1087	1	172081.5	C13 H18 O5	(M-H)-
254.1123	1	21120	C13 H18 O5	(M-H)-
325.1849	1	27620.53		
339.2007	1	30619.35		

Formula Calculator Element Limits

Element	Min	Max
C	3	60
H	0	120
O	0	30

Formula Calculator Results

Formula	CalculatedMass	CalculatedMz	Mz	Diff. (mDa)	Diff. (ppm)	DBE
C13 H18 O5	254.1154	253.1081	253.1087	-0.60	-2.37	5.0000

--- End Of Report ---

Fig. S43 ^1H NMR spectrum ($CDCl_3$, 500 MHz) of xylariahgin E (5)

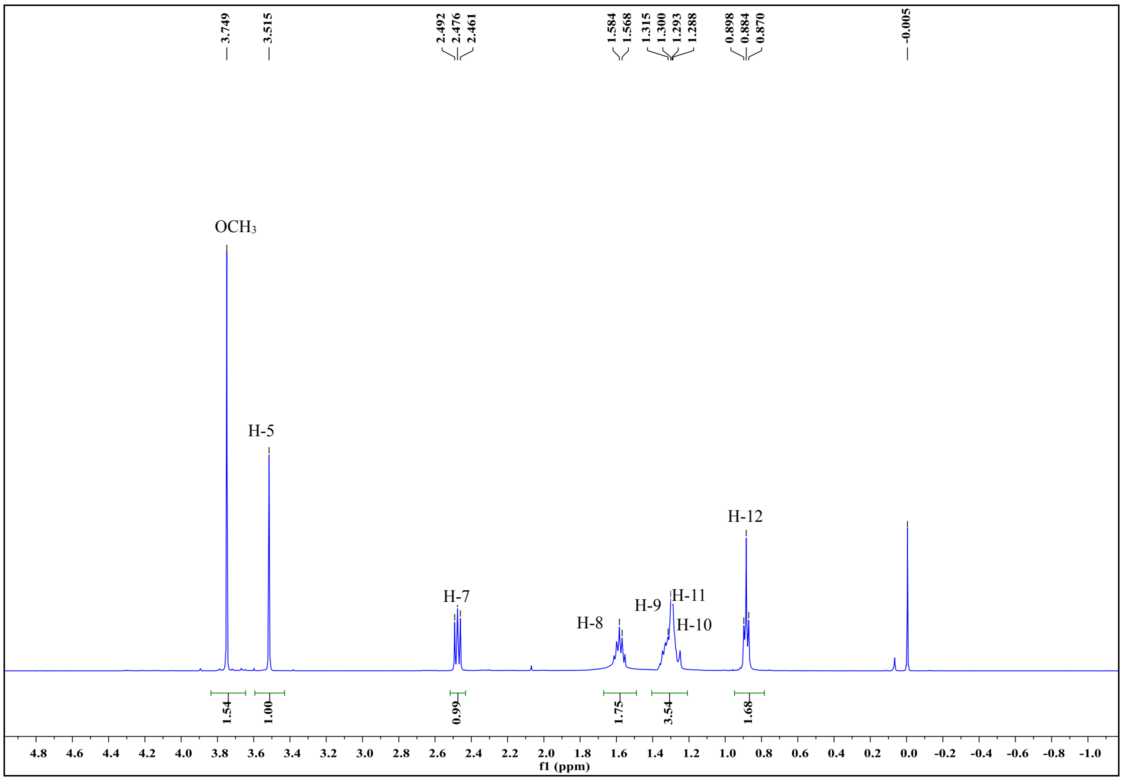

Fig. S44 ^{13}C NMR spectrum ($CDCl_3$, 500 MHz) of xylariahgin E (**5**)

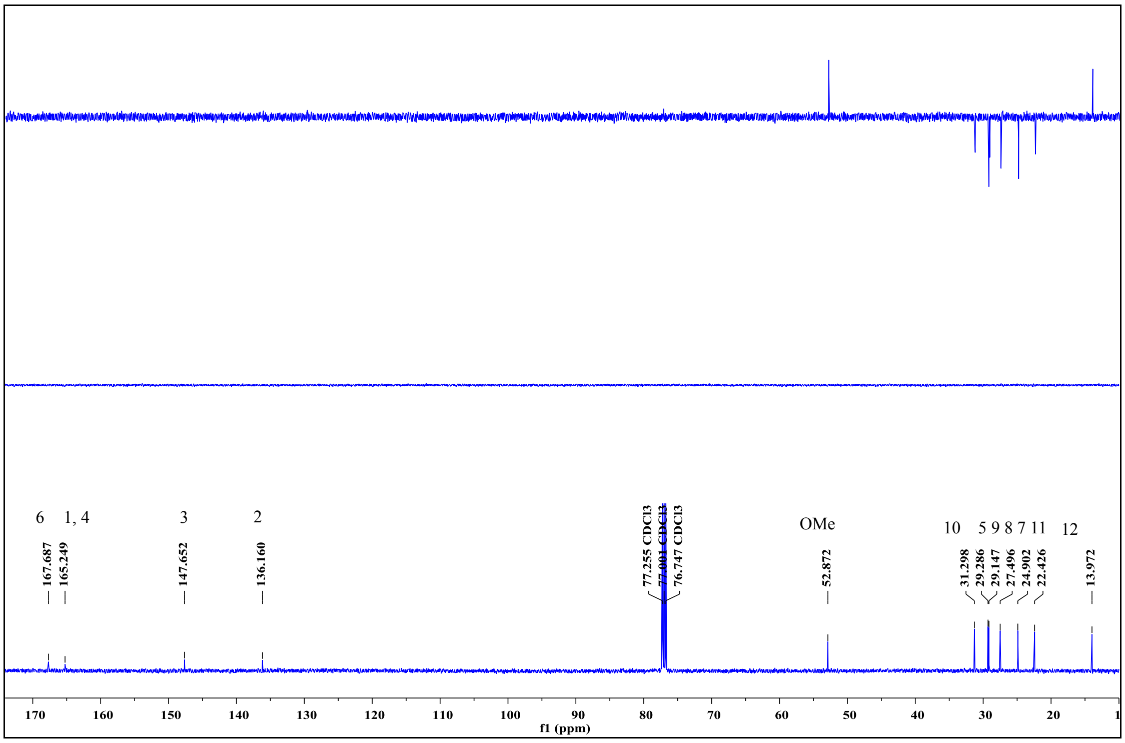

Fig. S45 HSQC spectrum ($CDCl_3$, 500 MHz) of xylariahgin E (**5**)

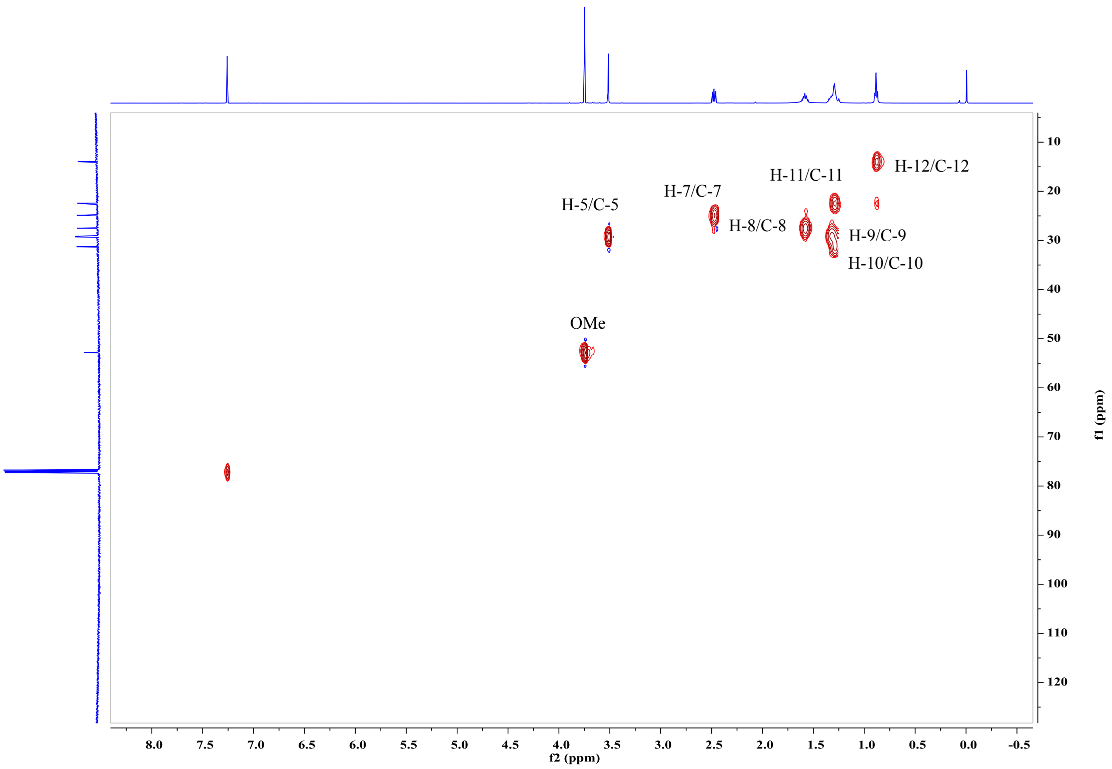

Fig. S46 ^1H-^1H COSY spectrum ($CDCl_3$, 500 MHz) of xylariahgin E (**5**)

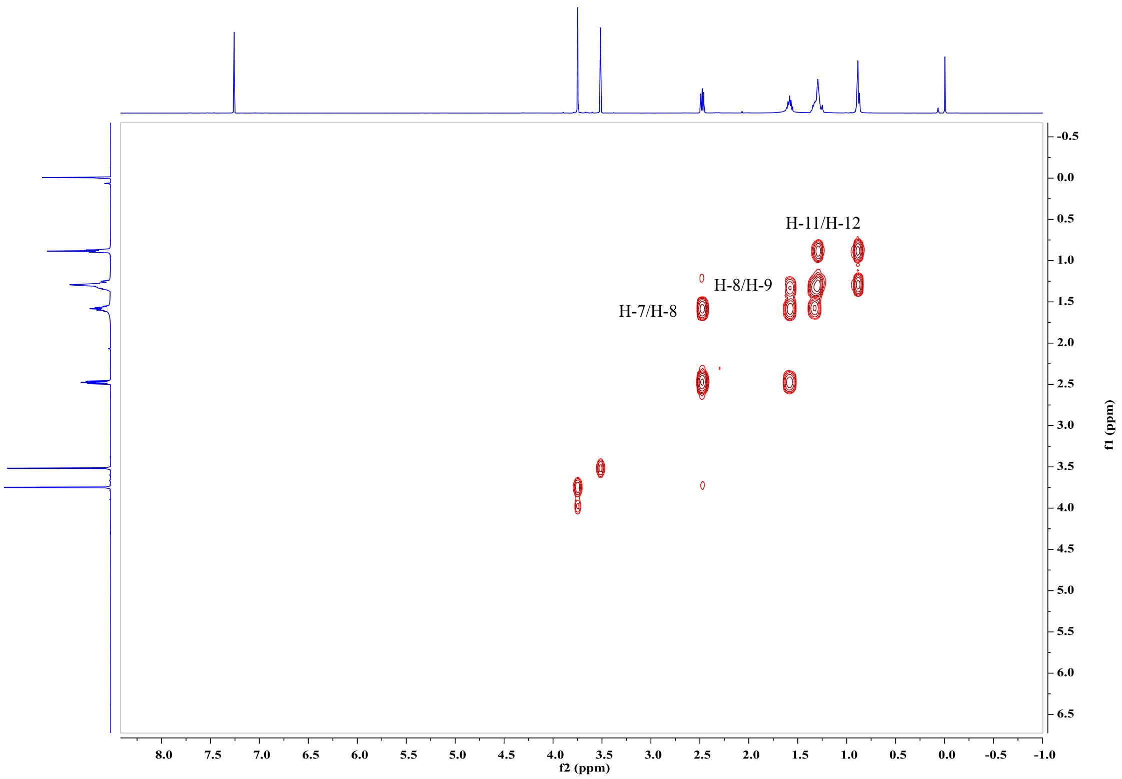

Fig. S47 HMBC spectrum ($CDCl_3$, 500 MHz) of xylariahgin E (**5**)

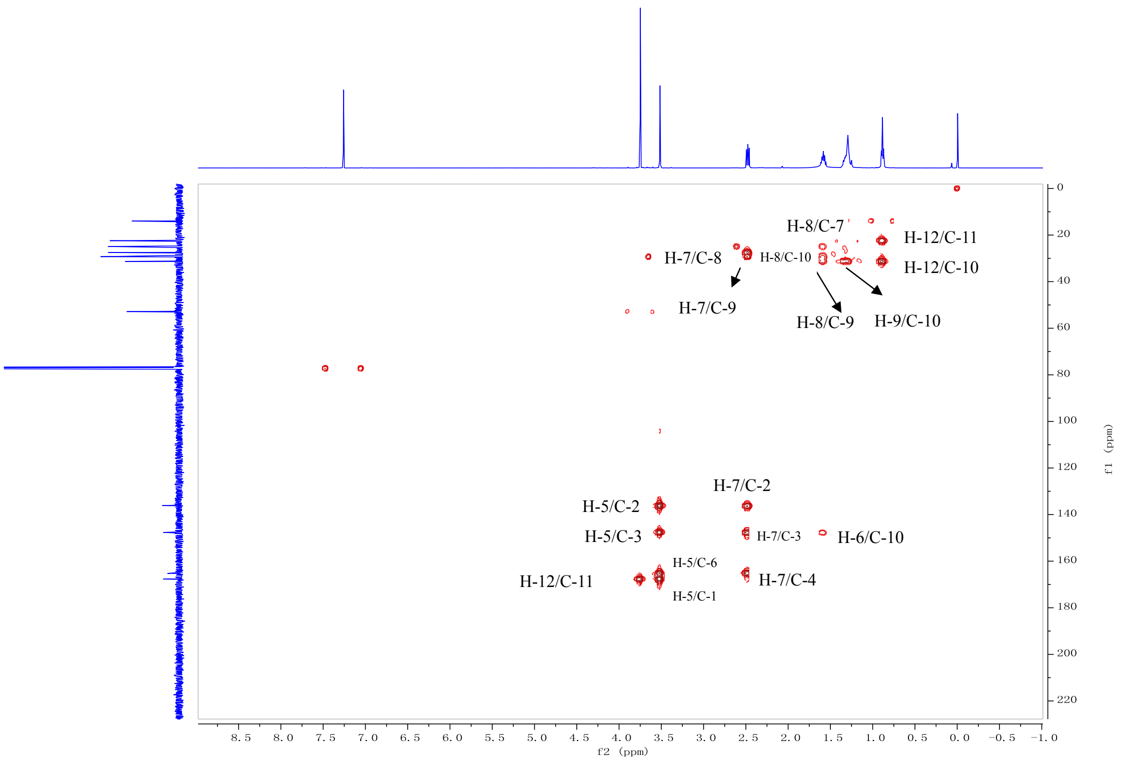

Fig. S48 ROESY spectrum ($CDCl_3$, 500 MHz) of xylariahgin E (**5**)

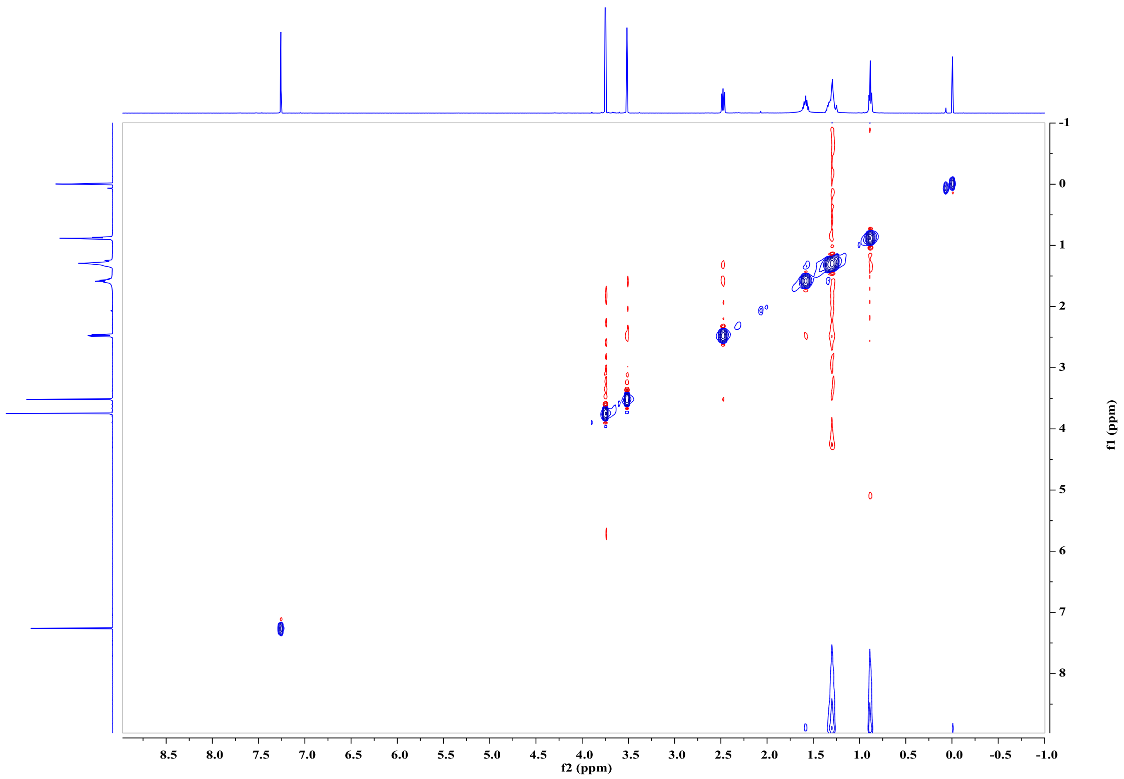

Fig. S49 UV spectrum of xylariahgin E (**5**)

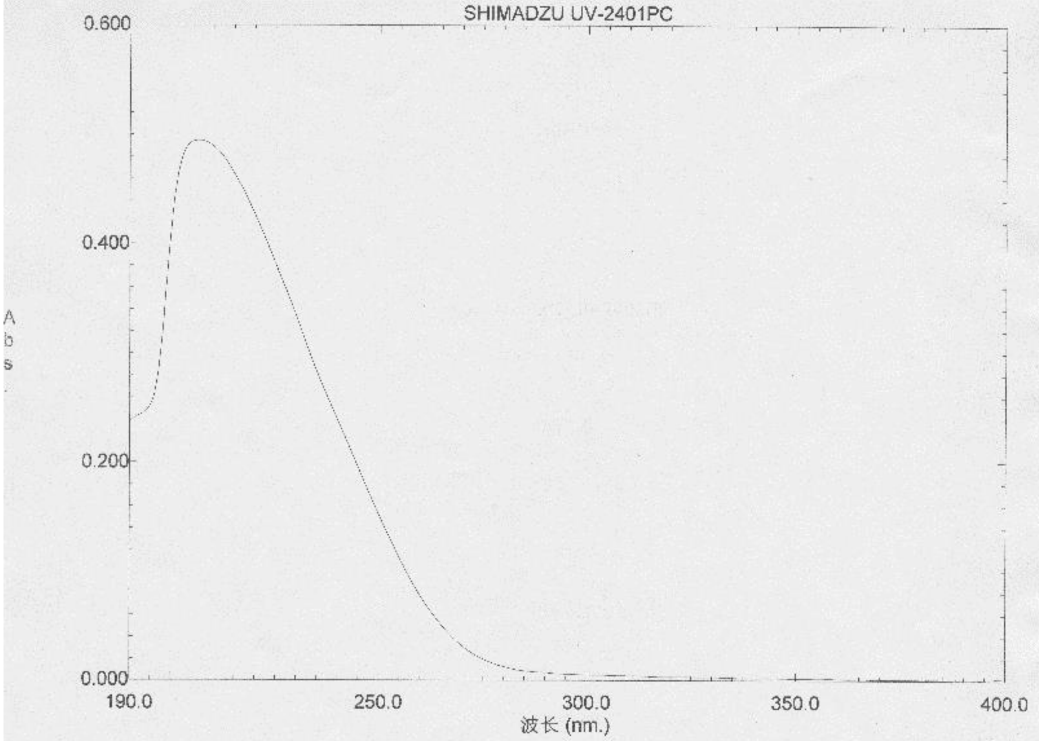

文件名: SCR56

SCR56 ———

样品浓度：0.0154毫克/毫升
溶剂：甲醇

创建于: 10:20 17-08-22
数据: 原始

测量模式: Abs.
扫描速度: 中速
狭缝: 5.0
采样间隔: 0.2

否.	波长 (nm.)	Abs.
1	206.60	0.4945

Fig. S50 IR spectrum of xylariahgin E (**5**)

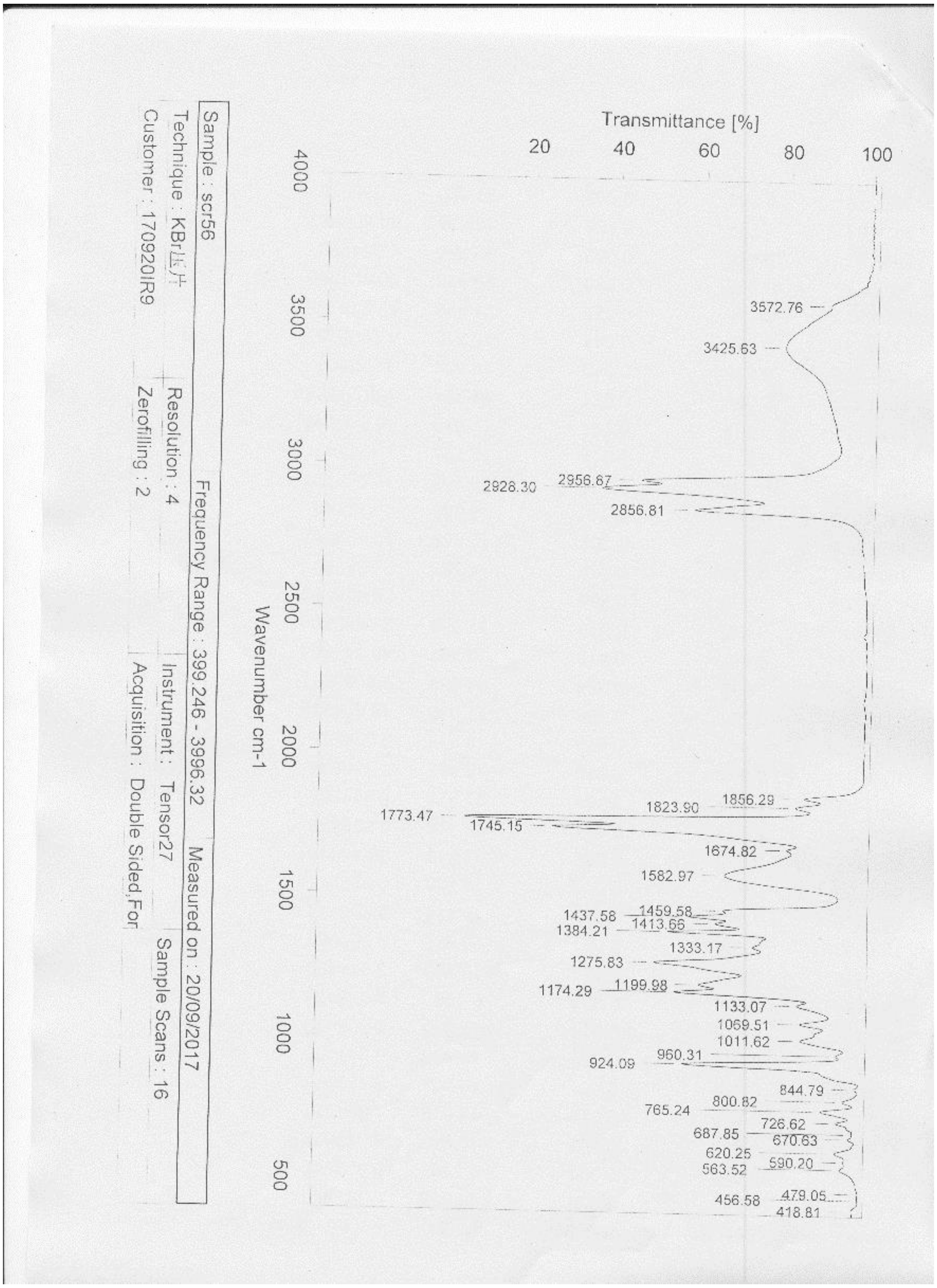

Fig. S51 HRESIMS spectrum of xylariahgin F (**6**)

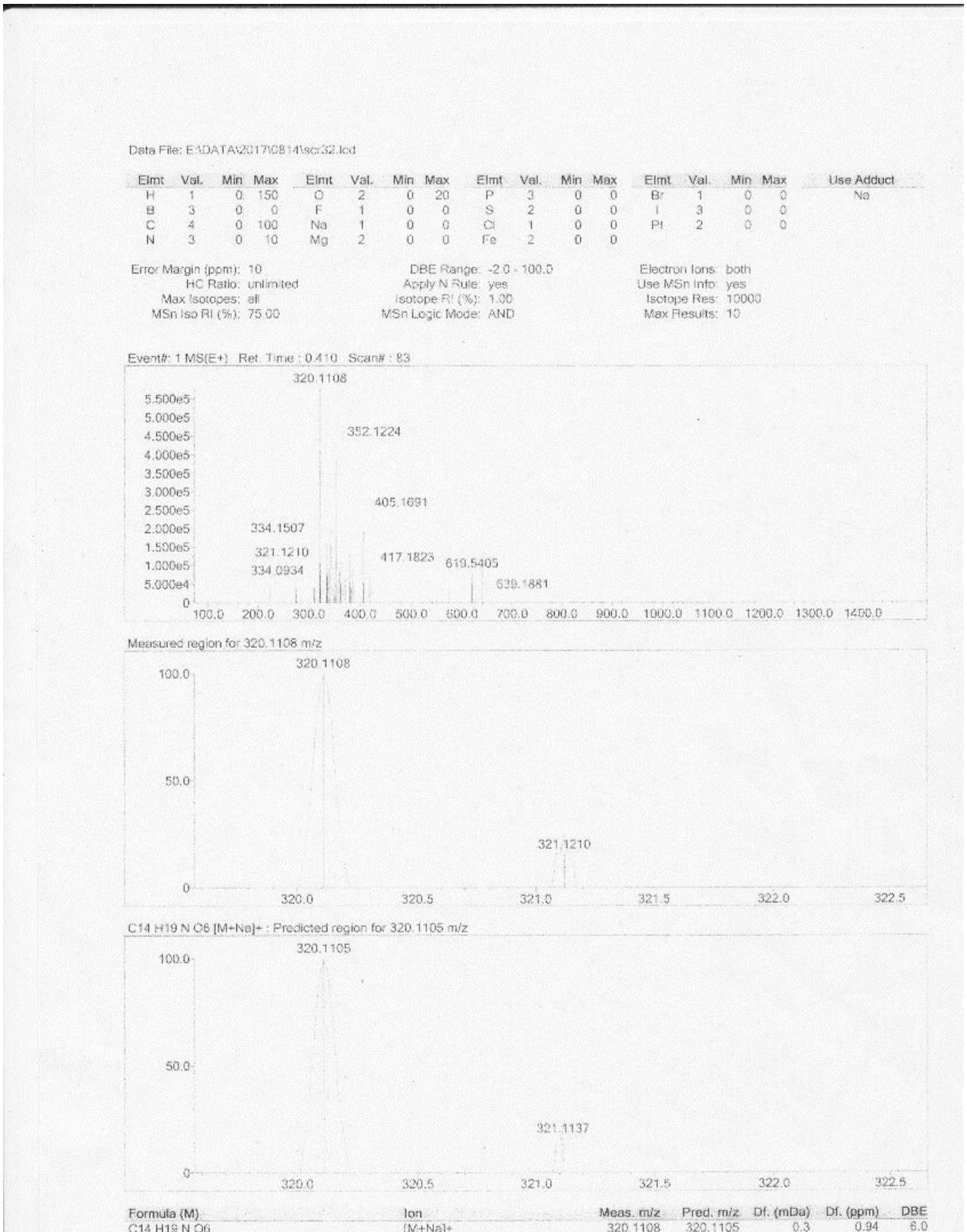

Formula (M)	Ion	Meas. m/z	Pred. m/z	Df. (mDa)	Df. (ppm)	DBE
C14 H19 N O6	[M+Na]+	320.1108	320.1105	0.3	0.94	6.0

Fig. S52 ^{1}H NMR spectrum ($CDCl_3$, 500 MHz) of xylariahgin F (**6**)

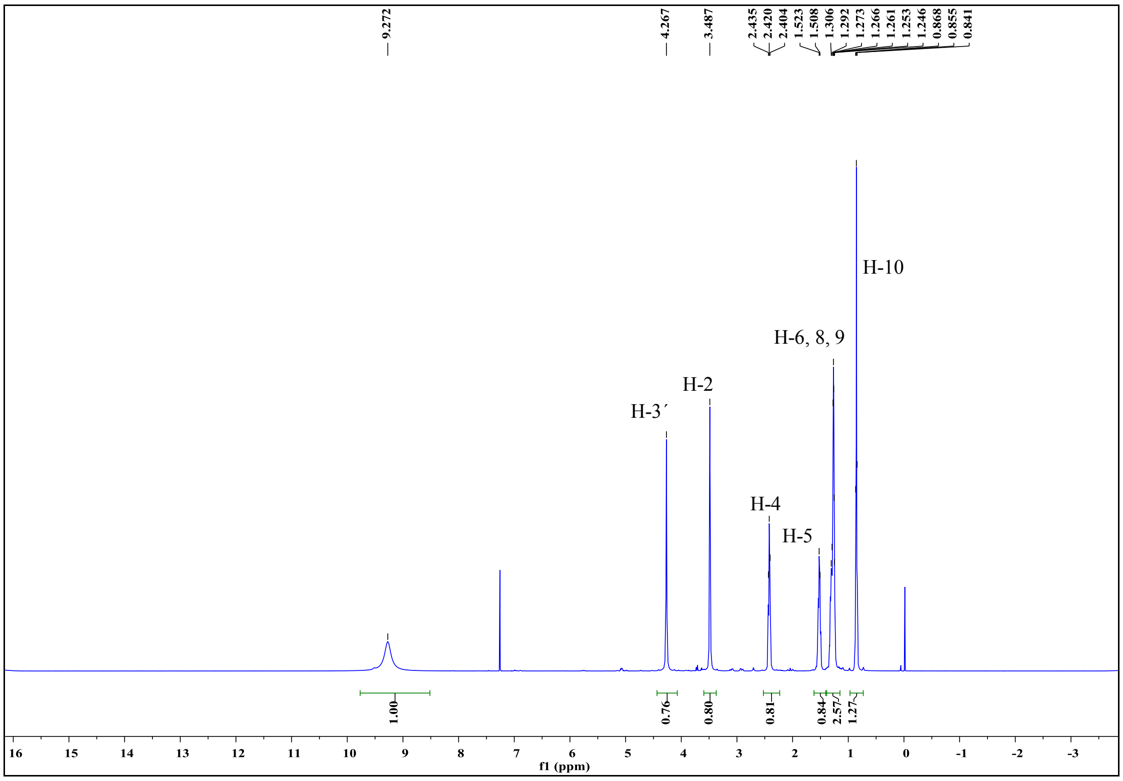

Fig. S53 ^{13}C NMR spectrum ($CDCl_3$, 500 MHz) of xylariahgin F (**6**)

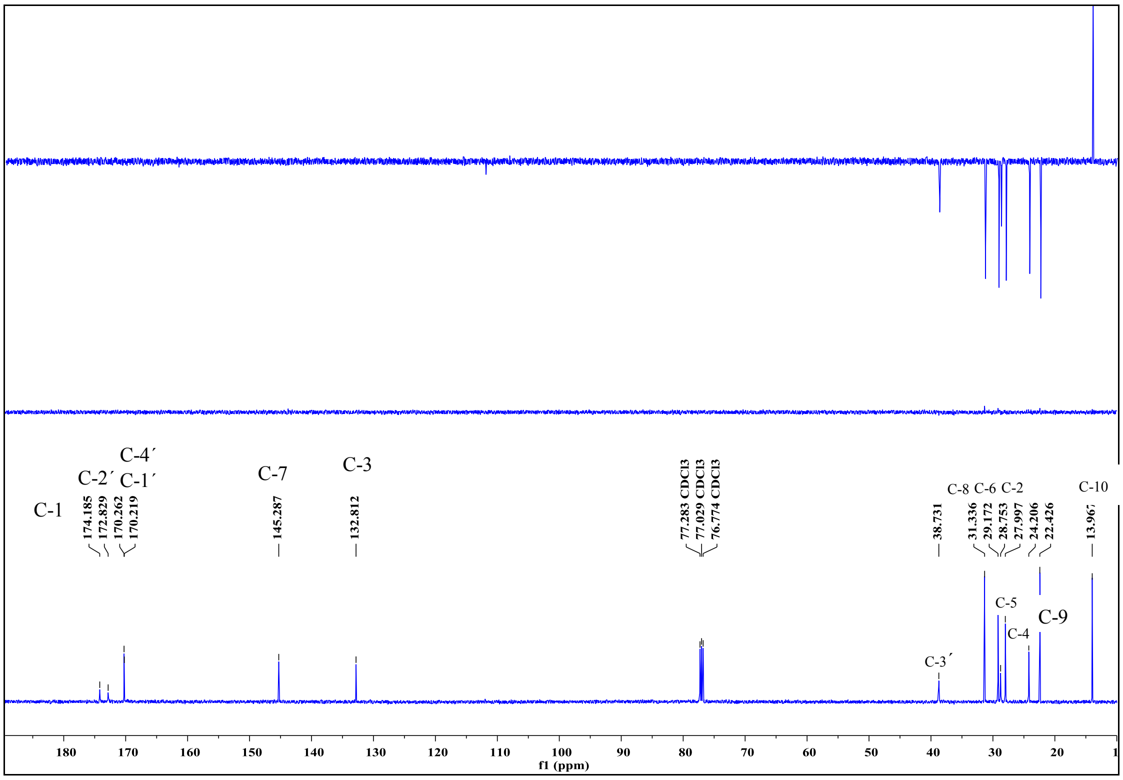

Fig. S54 HSQC spectrum ($CDCl_3$, 500 MHz) of xylariahgin F (**6**)

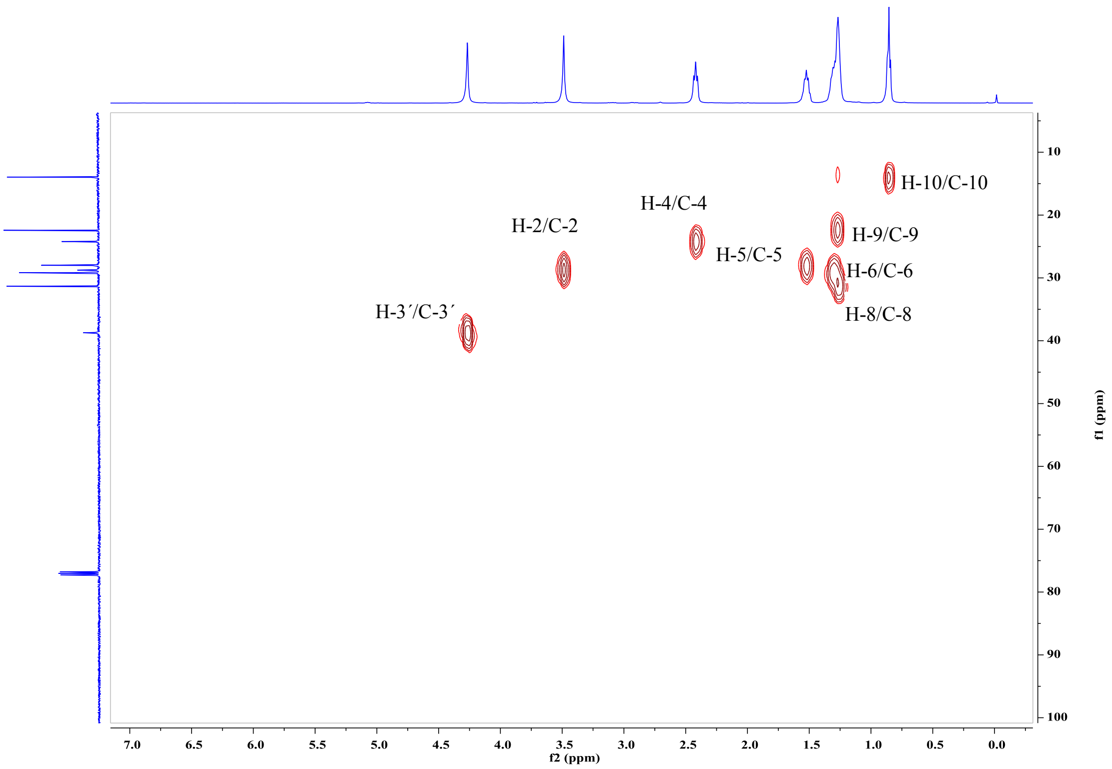

Fig. S55 ^{1}H-^{1}H COSY spectrum ($CDCl_3$, 500 MHz) of xylariahgin F (**6**)

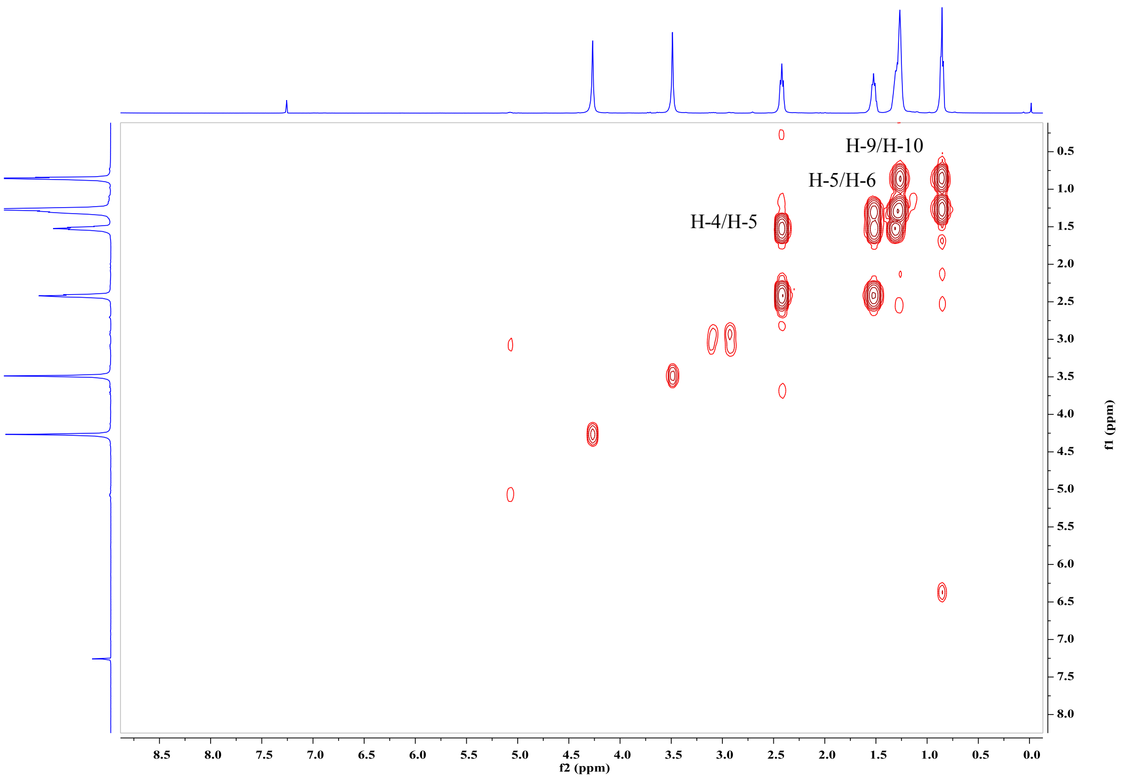

Fig. S56 HMBC spectrum ($CDCl_3$, 500 MHz) of xylariahgin F (**6**)

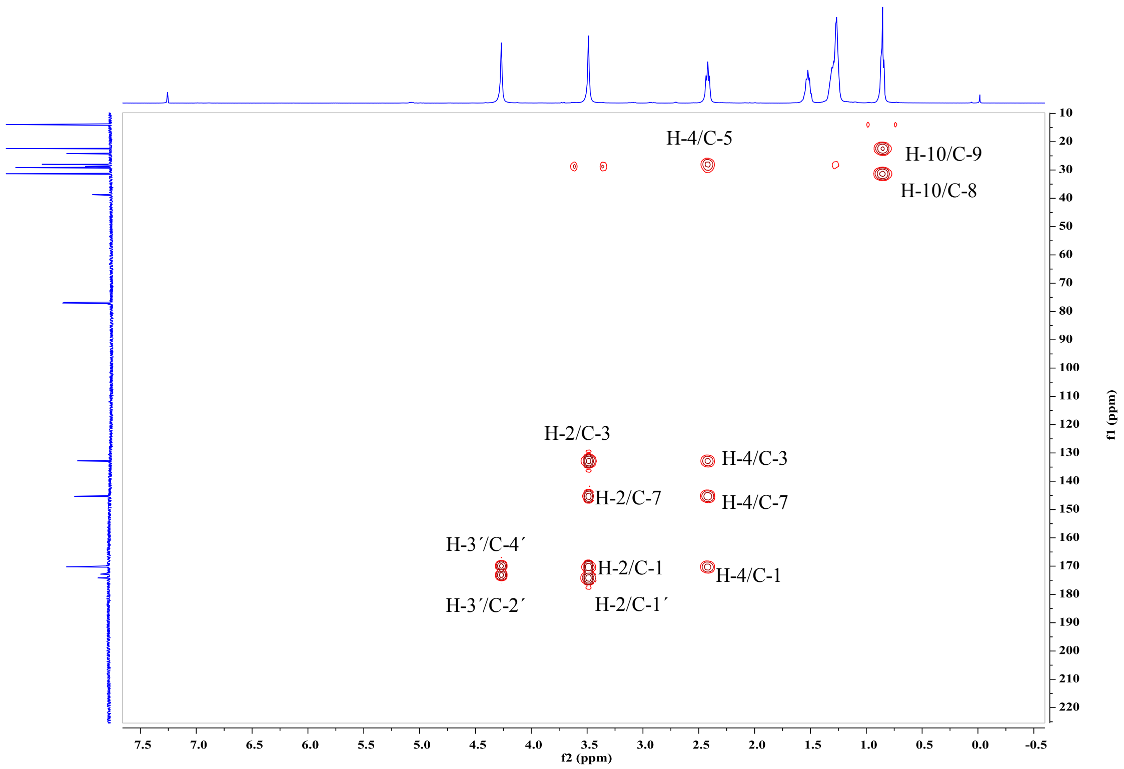

Fig. S57 ROESY spectrum ($CDCl_3$, 500 MHz) of xylariahgin F (**6**)

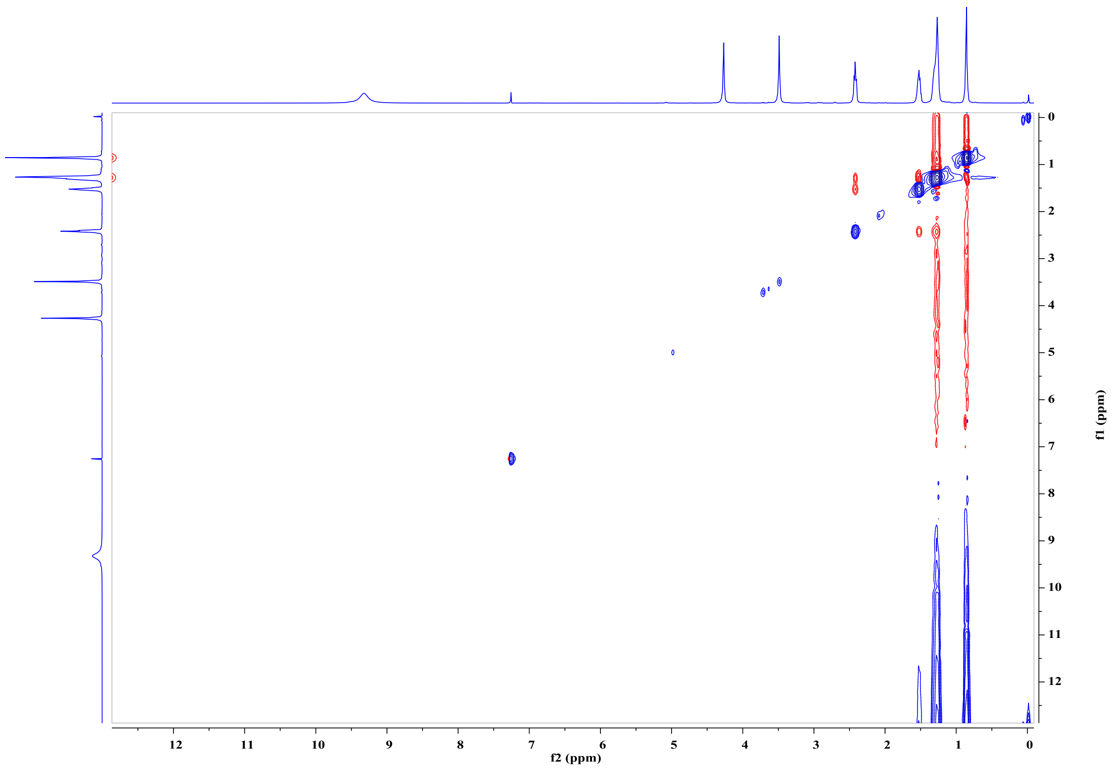

Fig. S58 UV spectrum of xylariahgin F (**6**)

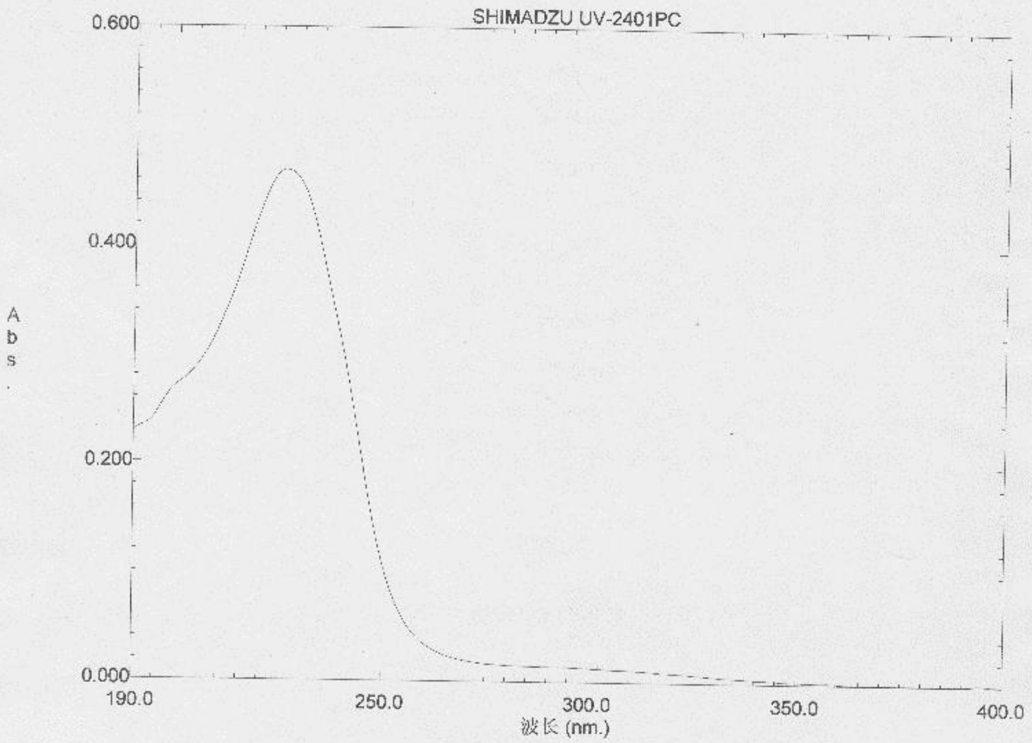

文件名: 17082200
样品名称：SCR32

17082200

样品浓度：0.0099毫克/毫升
溶剂：甲醇

创建于: 10:29 17-08-22
数据: 原始

测量模式: Abs.
扫描速度: 中速
狭缝: 5.0
采样间隔: 0.2

否.	波长 (nm.)	Abs.
1	201.40	0.2739
2	225.80	0.4686

Fig. S59 IR spectrum of xylariahgin F (**6**)

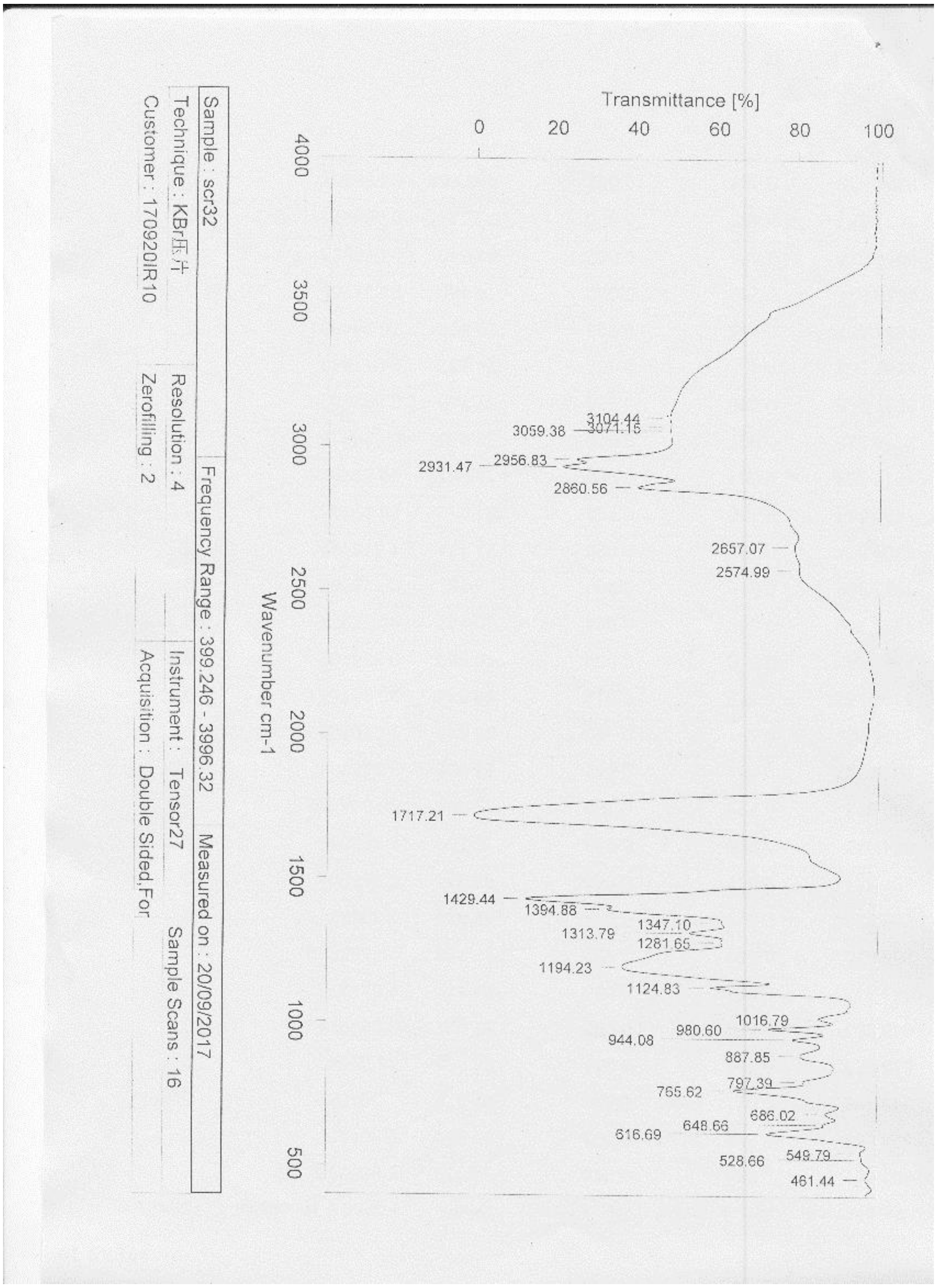

Fig. S60 EI$^+$ spectrum of xylariahgin F (**6**)

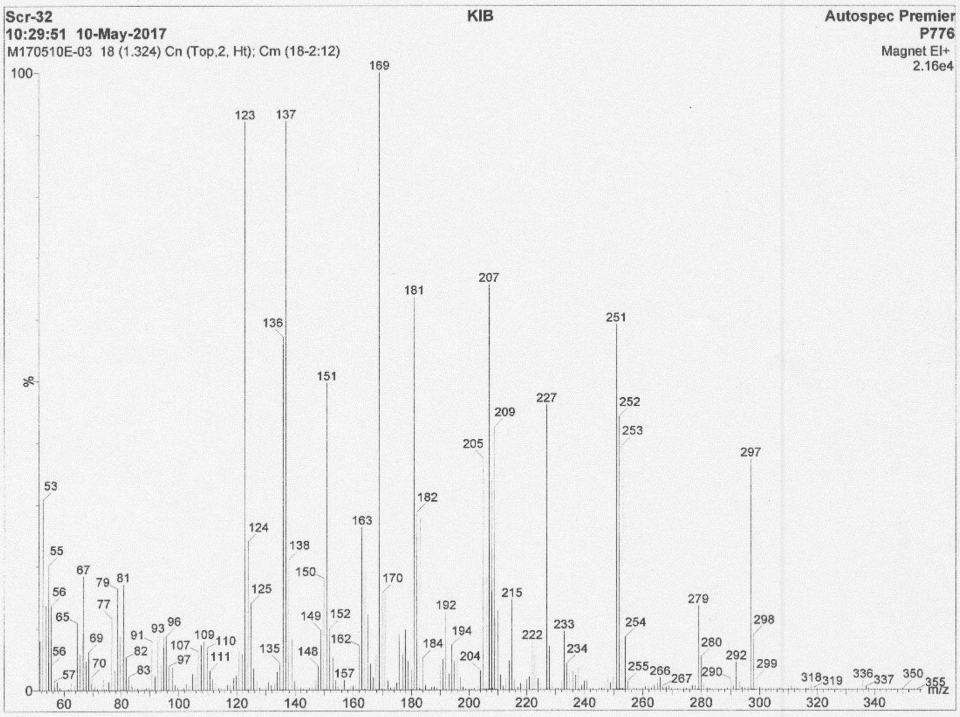

Fig. S61 Analysis of EI^+ spectrum of xylariahgin F (**6**)

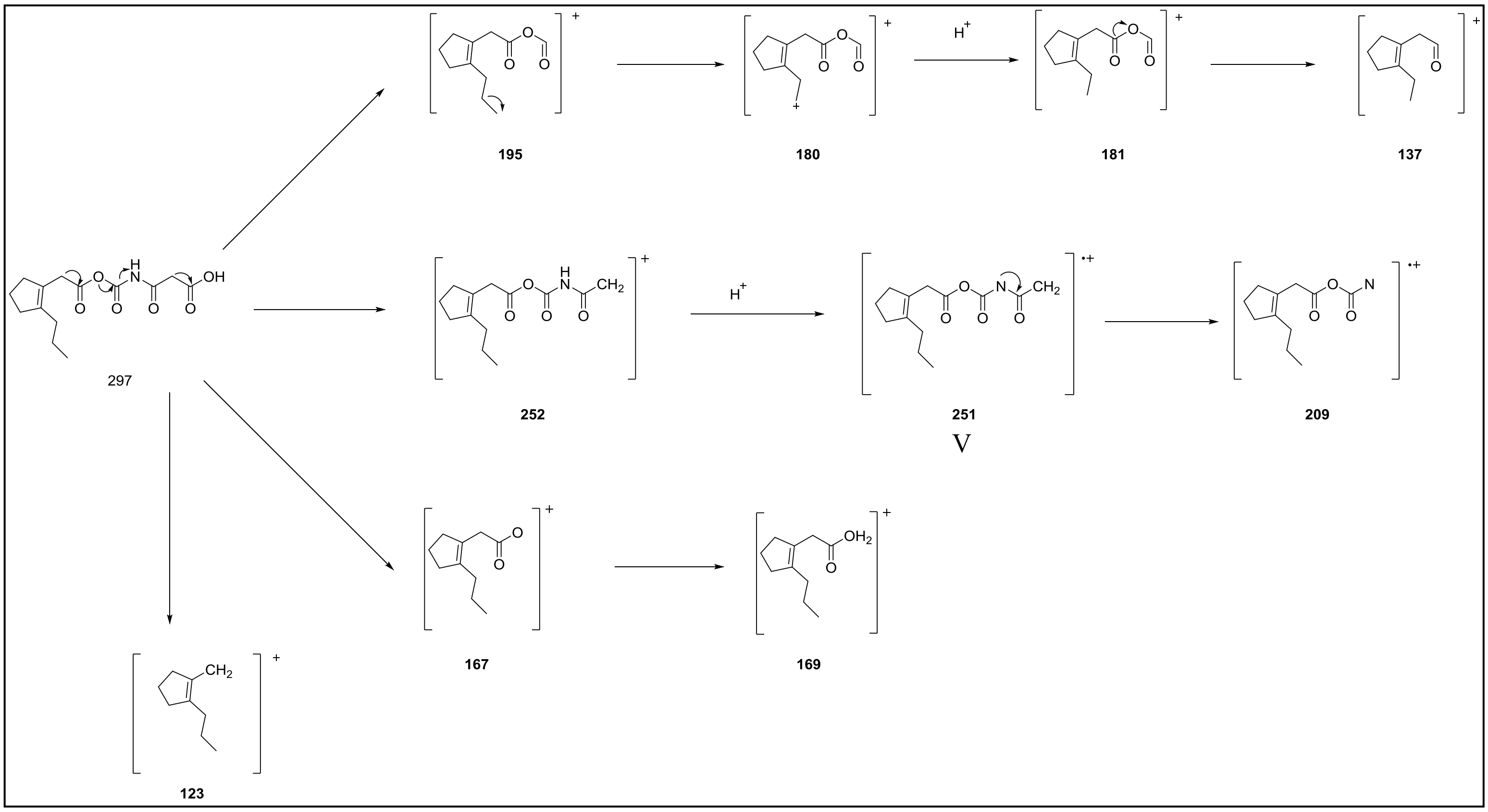

Fig. S62 HRESIMS spectrum of **7**

[illegible]

Data File: E:\DATA\2017\0814\scr28.lcd

Elmt	Val.	Min	Max	Elmt	Val.	Min	Max	Elmt	Val.	Min	Max	Elmt	Val.	Min	Max	Use Adduct
H	1	0	150	O	2	0	20	P	3	0	0	Br	1	0	0	Na
B	3	0	0	F	1	0	0	S	2	0	0	I	3	0	0	
C	4	0	100	Na	1	0	0	Cl	1	0	0	Pt	2	0	0	
N	3	0	10	Mg	2	0	0	Fe	2	0	0					

Error Margin (ppm): 10
HC Ratio: unlimited
Max Isotopes: all
MSn Iso RI (%): 75.00

DBE Range: -2.0 - 100.0
Apply N Rule: yes
Isotope RI (%): 1.00
MSn Logic Mode: AND

Electron Ions: both
Use MSn Info: yes
Isotope Res: 10000
Max Results: 10

Event#: 1 MS(E+) Ret. Time : 0.410 Scan# : 83

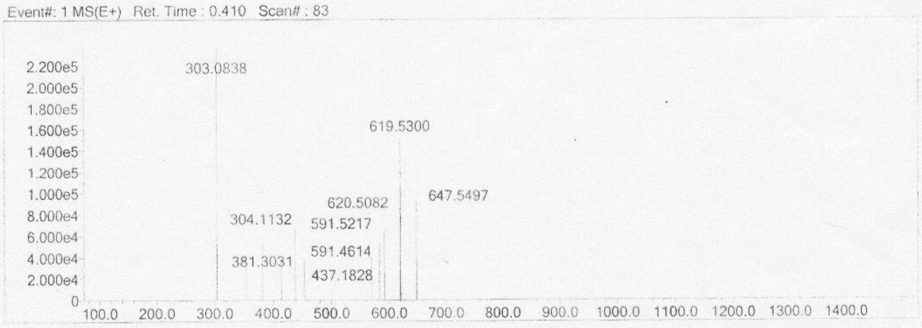

Measured region for 303.0838 m/z

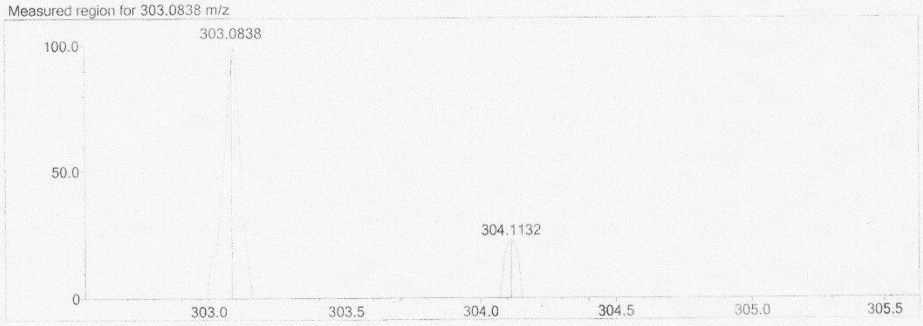

C14 H16 O6 [M+Na]+ : Predicted region for 303.0839 m/z

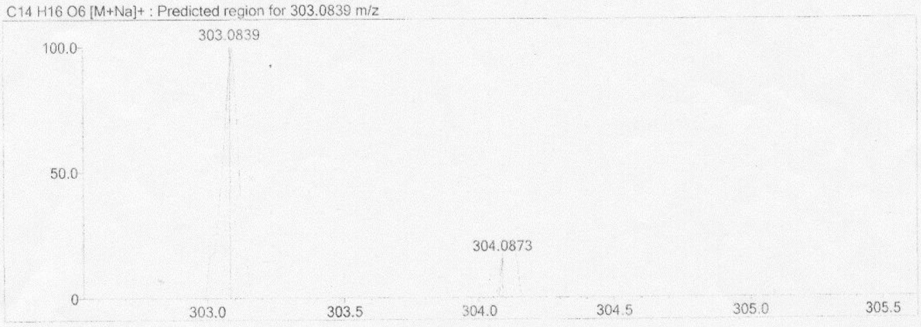

Formula (M)	Ion	Meas. m/z	Pred. m/z	Df. (mDa)	Df. (ppm)	DBE
C14 H16 O6	[M+Na]+	303.0838	303.0839	-0.1	-0.33	7.0

Fig. S63 ^{1}H NMR spectrum (CD_3OD, 600 MHz) of **7**

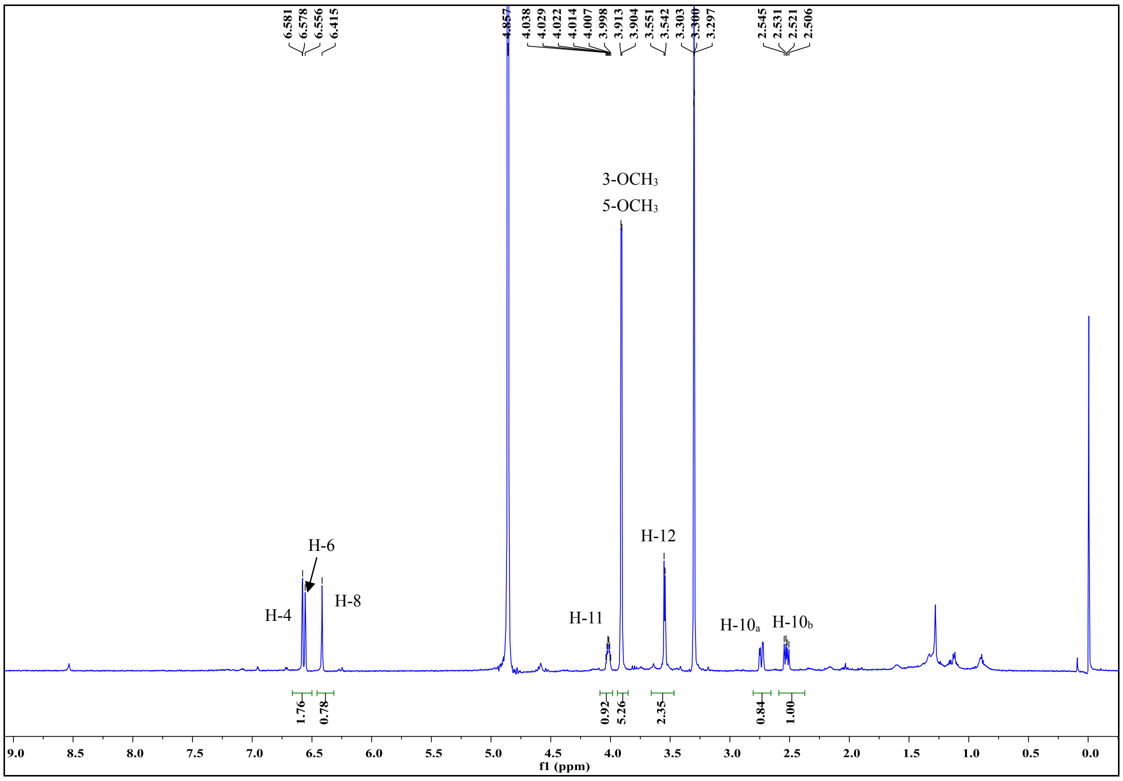

Fig. S64 ^{13}C NMR spectrum (CD_3OD, 600 MHz) of **7**

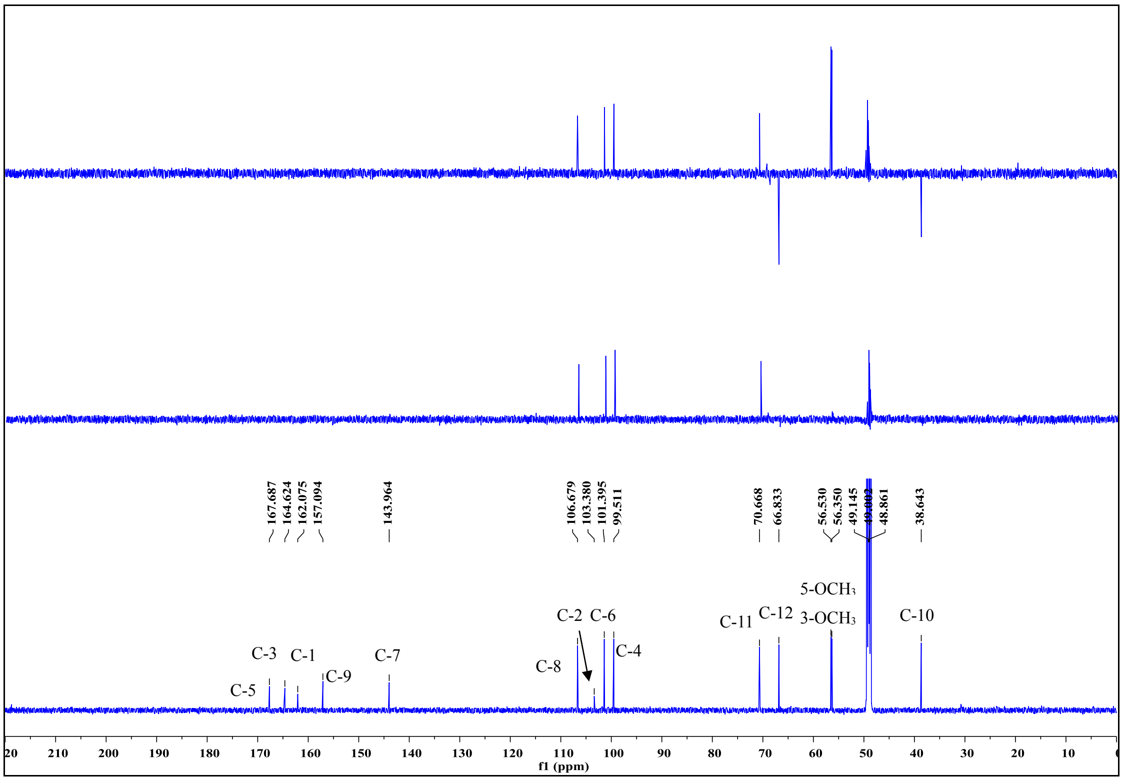

Fig. S65 HSQC spectrum (CD_3OD, 600 MHz) of **7**

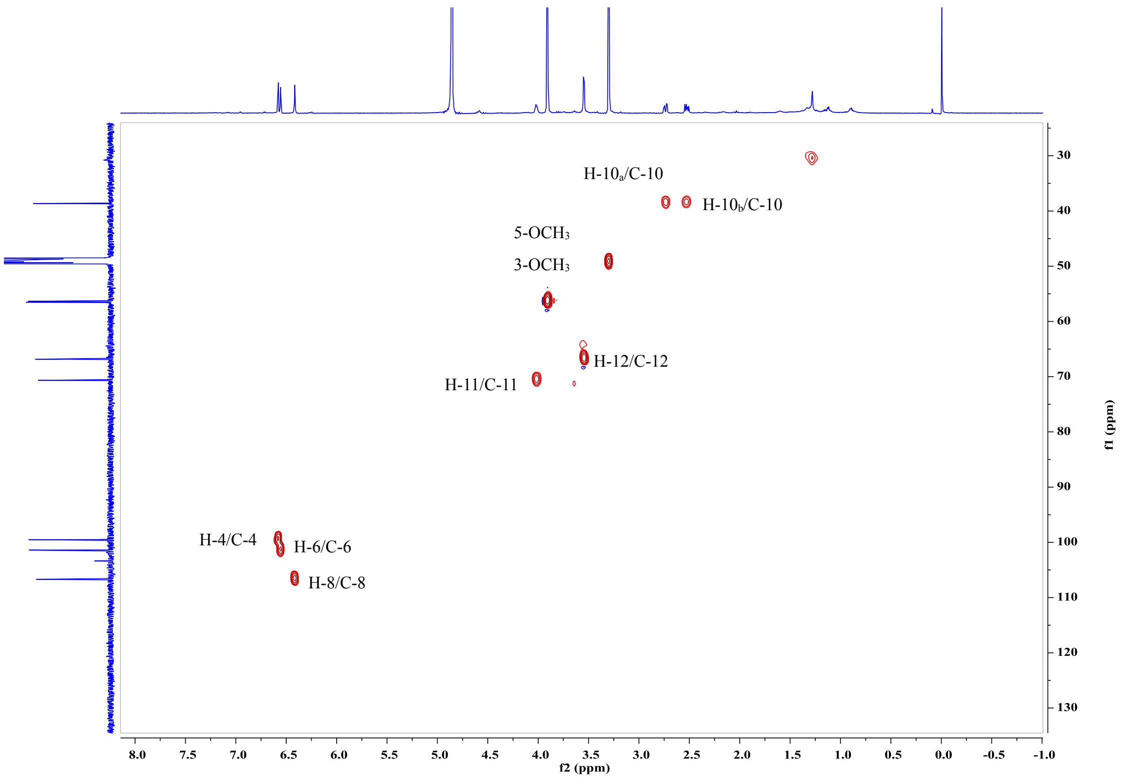

Fig. S66 ^{1}H-^{1}H COSY spectrum (CD_3OD, 600 MHz) of **7**

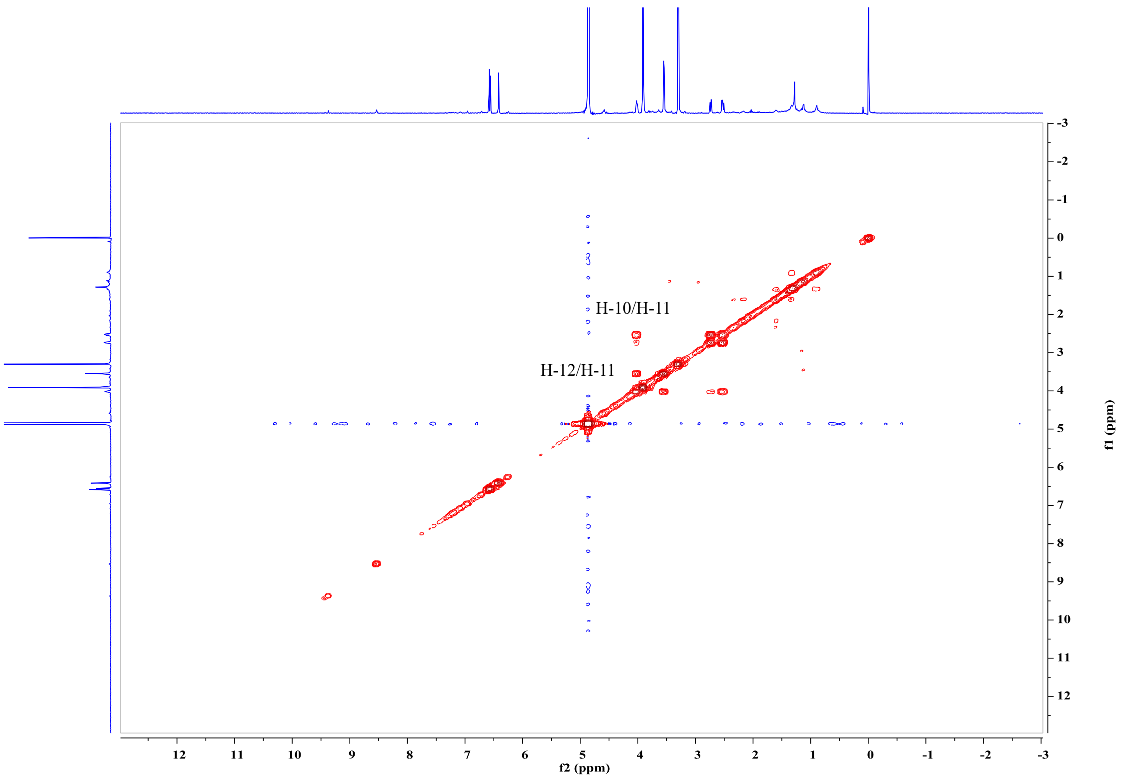

Fig. S67 HMBC spectrum (CD_3OD, 600 MHz) of **7**

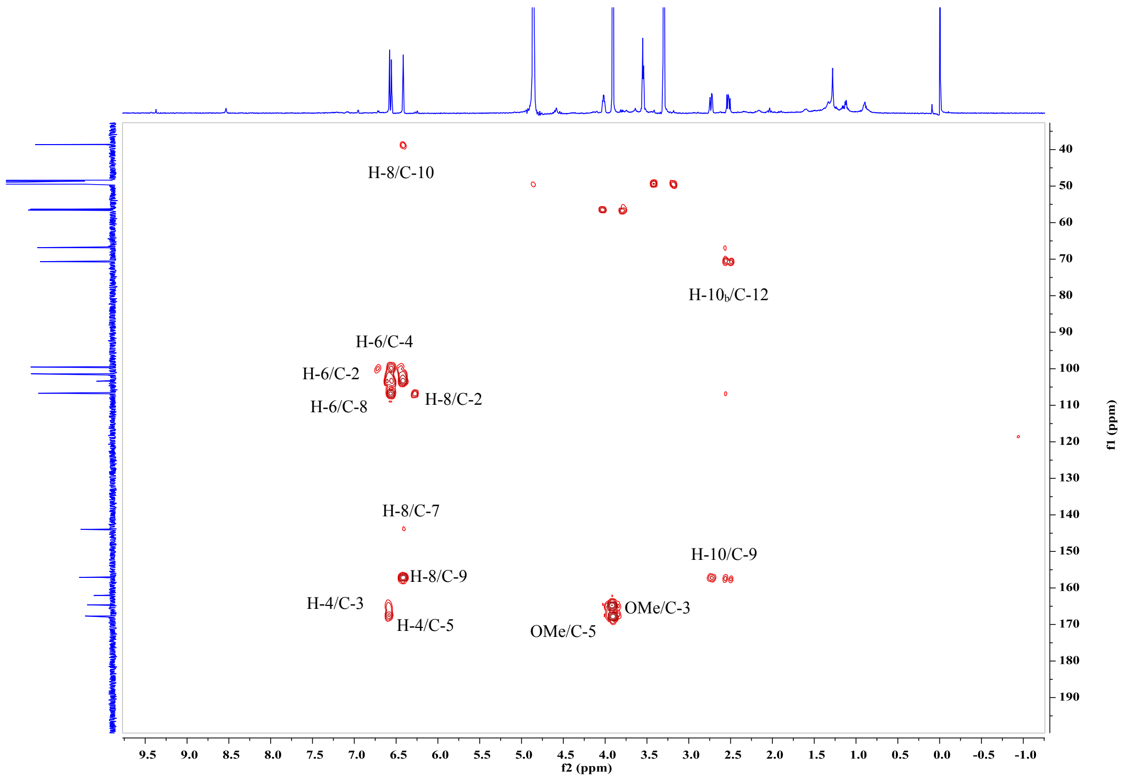

Fig. S68 ROESY spectrum (CD_3OD, 600 MHz) of **7**

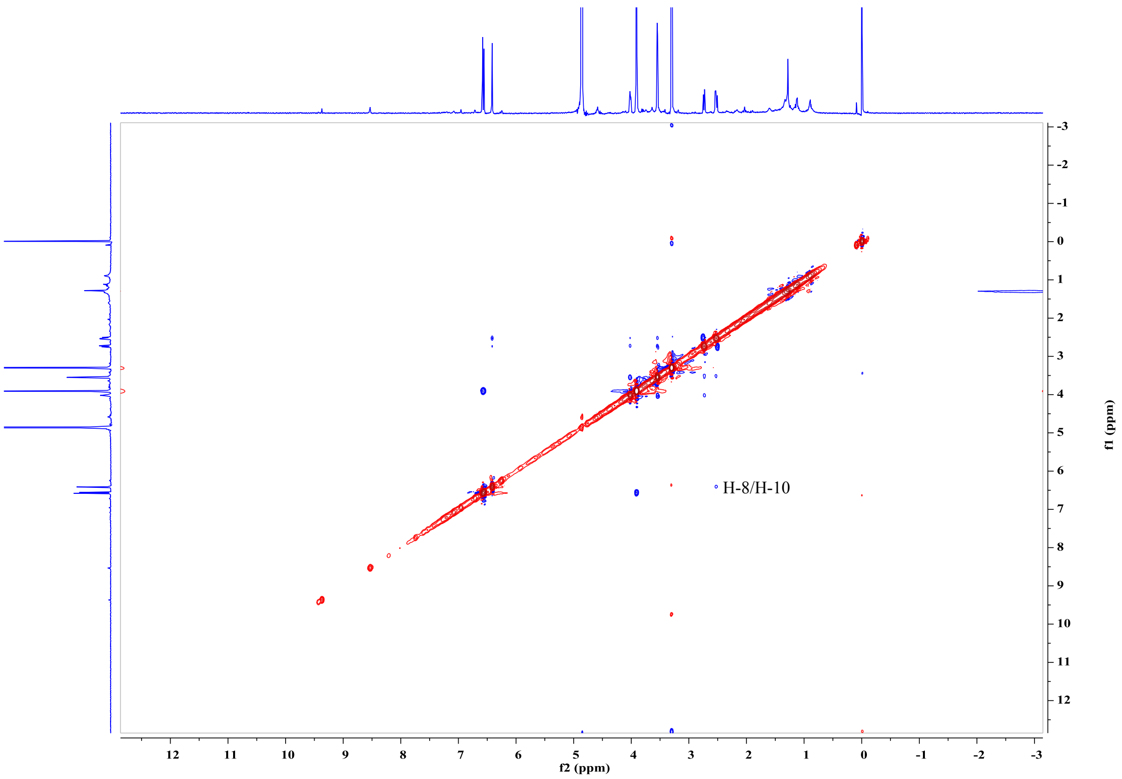

Fig. S69 UV spectrum of **7**

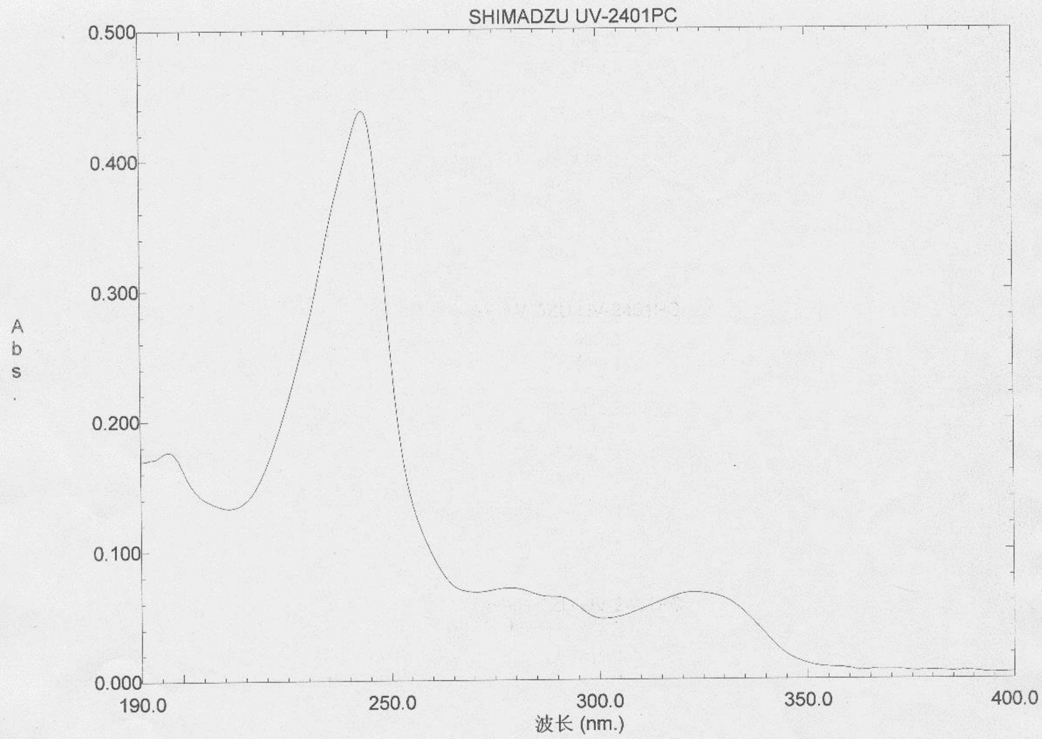

文件名: SCR28

SCR28 ———

样品浓度：0.0034毫克/毫升
溶剂：甲醇

创建于: 17:11 17-08-28
数据: 原始

测量模式: Abs.
扫描速度: 中速
狭缝: 5.0
采样间隔: 0.5

否.	波长 (nm.)	Abs.
1	196.50	0.1762
2	242.50	0.4349
3	278.50	0.0711
4	287.50	0.0644
5	323.00	0.0669

Fig. S70 [α] spectrum of **7**

Optical rotation measurement

Model : P-1020 (A060460638)

No.	Sample	Mode	Data	Monitor<br>Blank	Temp.<br>Cell<br>Temp Point	Date<br>Comment<br>Sample Name	Light<br>Filter<br>Operator	Cycle Time<br>Integ Time
No.1	10 (1/3)	Sp.Rot	-7.1430	-0.0010<br>0.0000	23.8<br>10.00<br>Cell	Mon Jul 24 11:51:03 2017<br>0.00140g/mL MeOH<br>SCR28	Na<br>589nm	2 sec<br>2 sec
No.2	10 (2/3)	Sp.Rot	-5.7140	-0.0008<br>0.0000	23.8<br>10.00<br>Cell	Mon Jul 24 11:51:08 2017<br>0.00140g/mL MeOH<br>SCR28	Na<br>589nm	2 sec<br>2 sec
No.3	10 (3/3)	Sp.Rot	-7.1430	-0.0010<br>0.0000	23.7<br>10.00<br>Cell	Mon Jul 24 11:51:14 2017<br>0.00140g/mL MeOH<br>SCR28	Na<br>589nm	2 sec<br>2 sec

-6.6667°

Fig. S71 IR spectrum of **7**

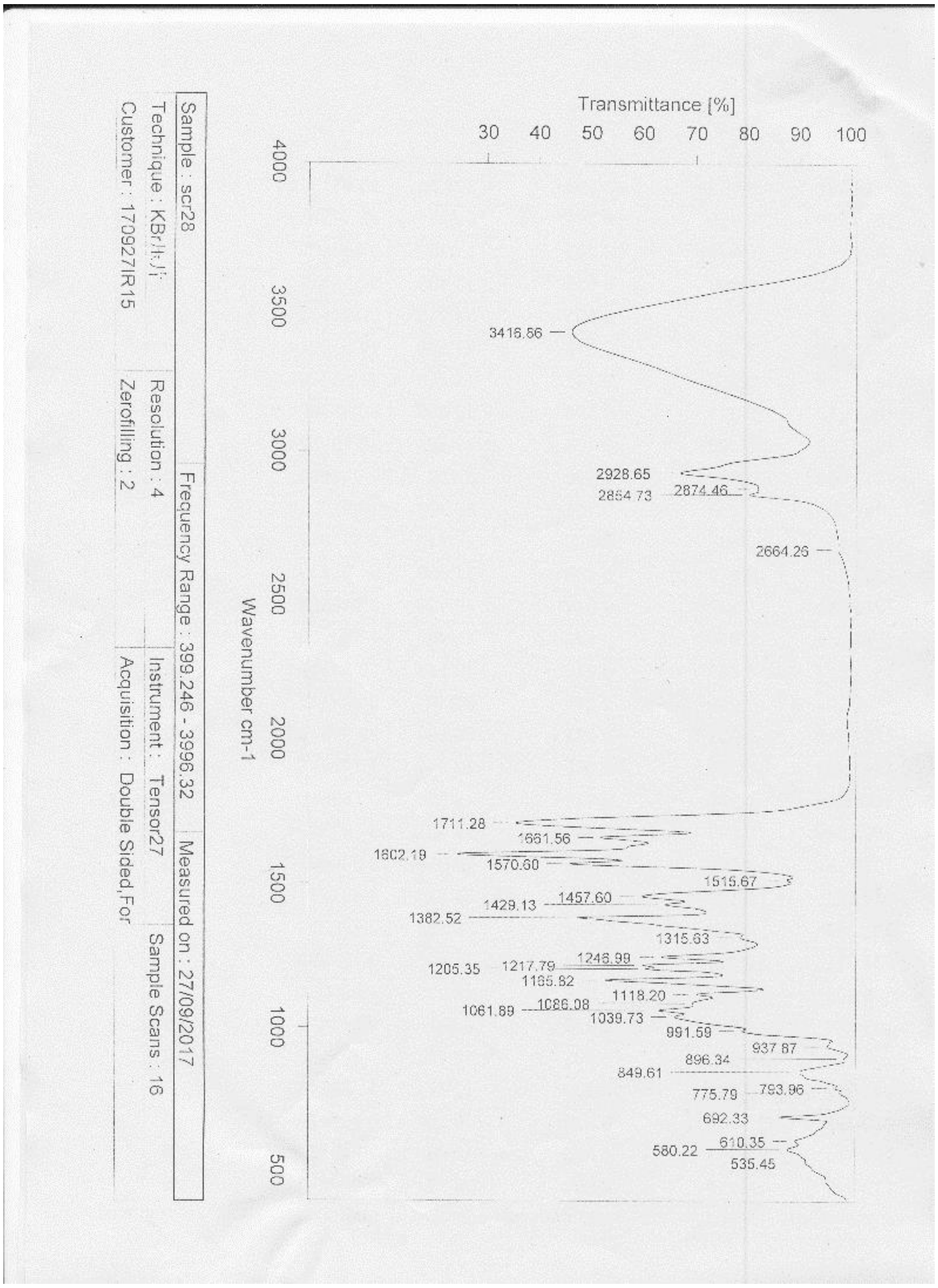

Fig. S72 HRESIMS spectrum of **8**

Qualitative Analysis Report

**Data Filename**	sh917c2-5.d	**Sample Name**	sh917c2-5
**Sample Type**	Sample	**Position**	P1-F8
**Instrument Name**	Instrument 1	**User Name**	
**Acq Method**	SIBU.m	**Acquired Time**	3/24/2016 3:29:48 PM
**IRM Calibration Status**	Success	**DA Method**	ESI+.m
**Comment**			

Sample Group **Info.**
Acquisition SW Version 6200 series TOF/6500 series Q-TOF B.05.01 (B5125.2)

User Spectra

Fragmentor Voltage	Collision Energy	Ionization Mode
135	0	ESI

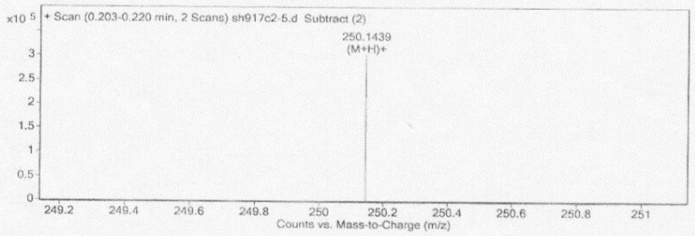

Peak List

m/z	z	Abund	Formula	Ion
250.1439	1	304652.63	C14 H19 N O3	(M+H)+
251.1471	1	41812.66	C14 H19 N O3	(M+H)+
272.1258	1	125902.4		
273.1292	1	20086.51		
288.0997	1	43481		
499.2803	1	44661.1		
521.2624	1	114888.23		
522.2661	1	33530.77		

Formula Calculator Element Limits

Element	Min	Max
C	3	60
H	0	120
O	0	30
N	0	10

Formula Calculator Results

Formula	CalculatedMass	CalculatedMz	Mz	Diff. (mDa)	Diff. (ppm)	DBE
C14 H19 N O3	249.1365	250.1438	250.1439	-0.1	-0.3	6.0000

--- End Of Report ---

Fig. S73 ^{1}H NMR spectrum (Acetone-d_6, 600 MHz) of **8**

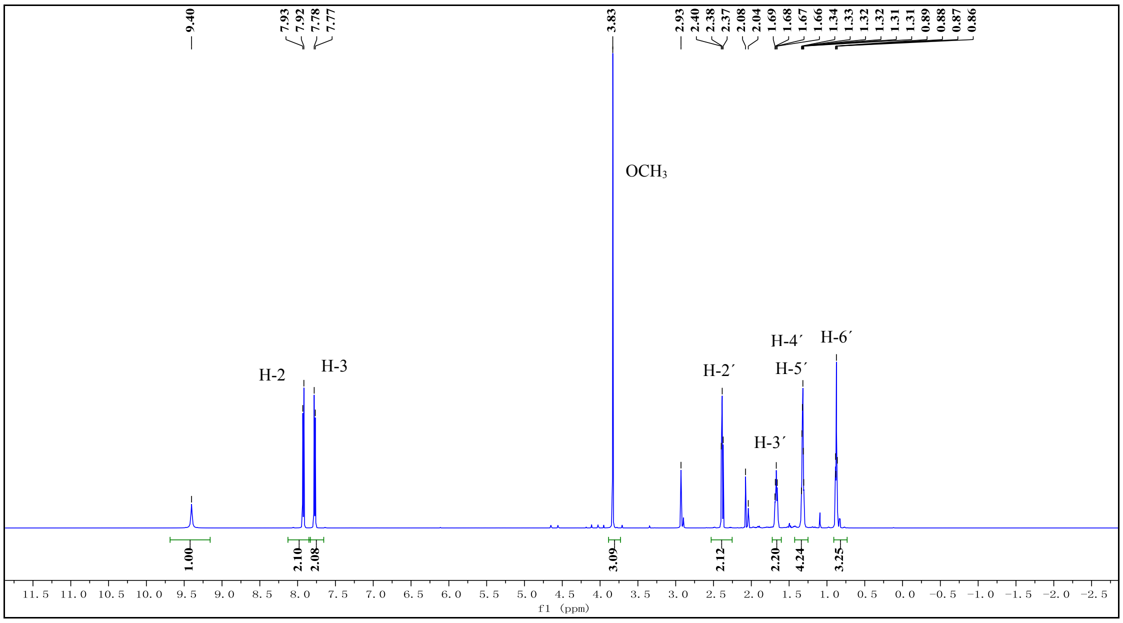

Fig. S74 ^{13}C NMR spectrum (Acetone-d_6, 600 MHz) of **8**

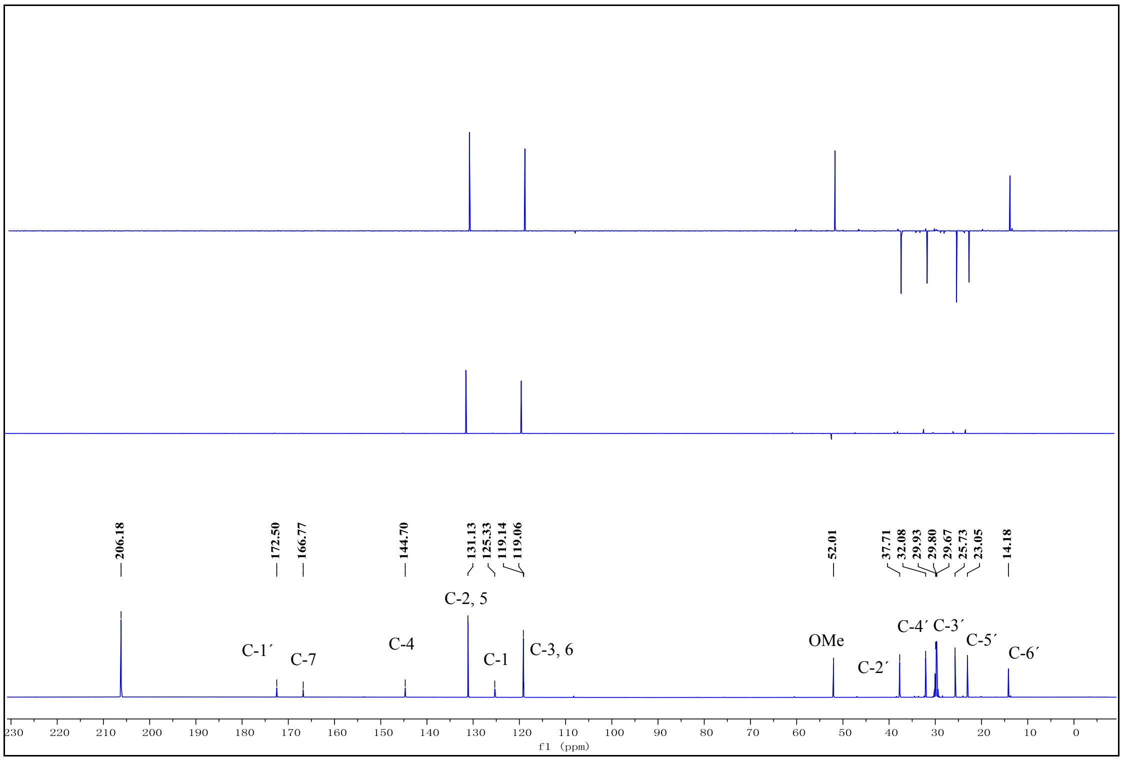

Fig. S75 HSQC spectrum (Acetone-d_6, 600 MHz) of **8**

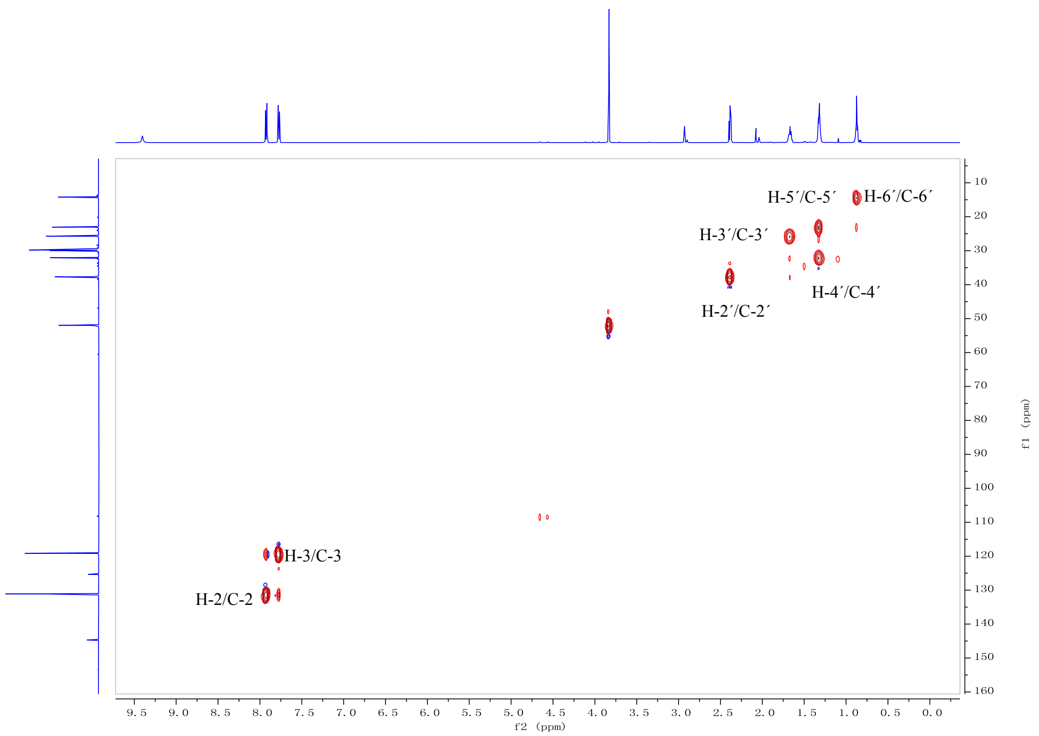

Fig. S76 ^{1}H-^{1}H COSY spectrum (Acetone-d_6, 600 MHz) of **8**

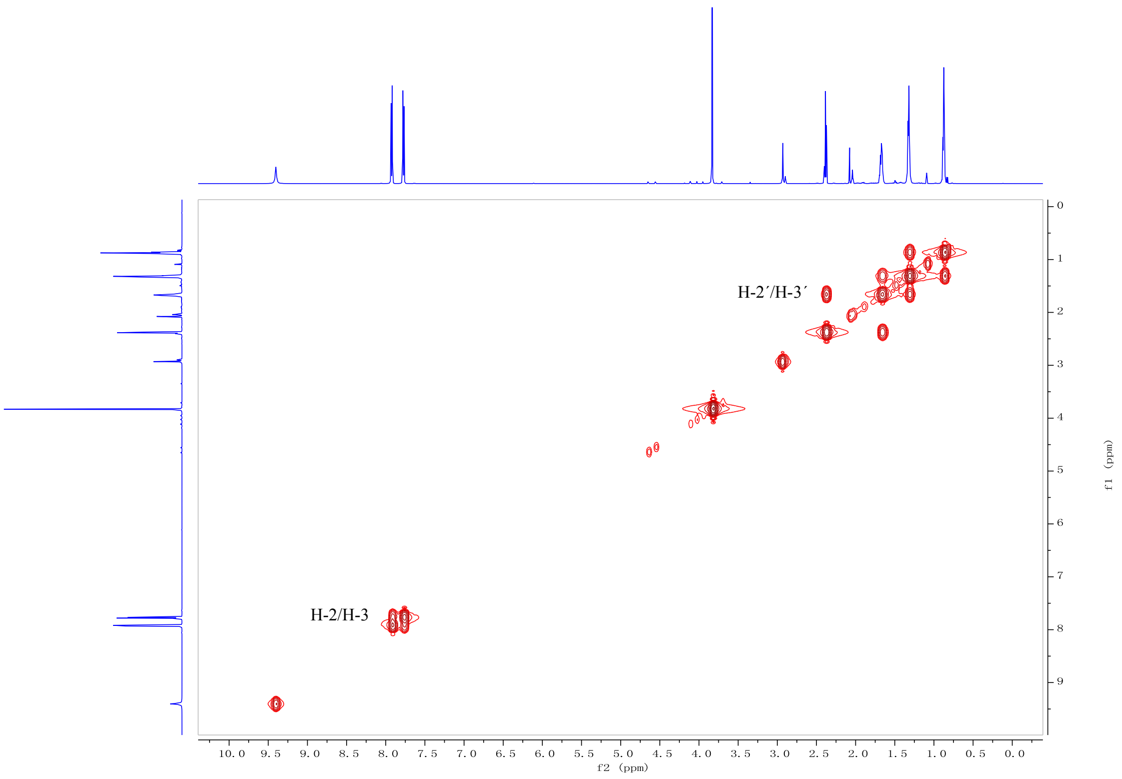

Fig. S77 HMBC spectrum (Acetone-d_6, 600 MHz) of **8**

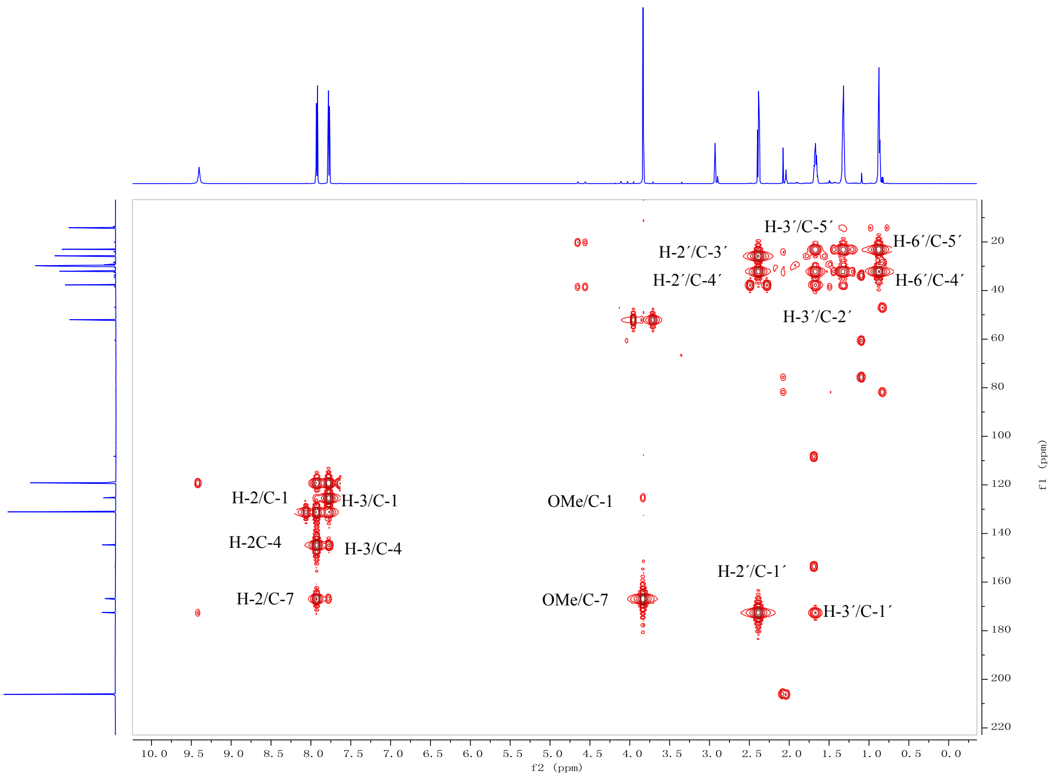

Fig. S78 UV spectrum of **8**

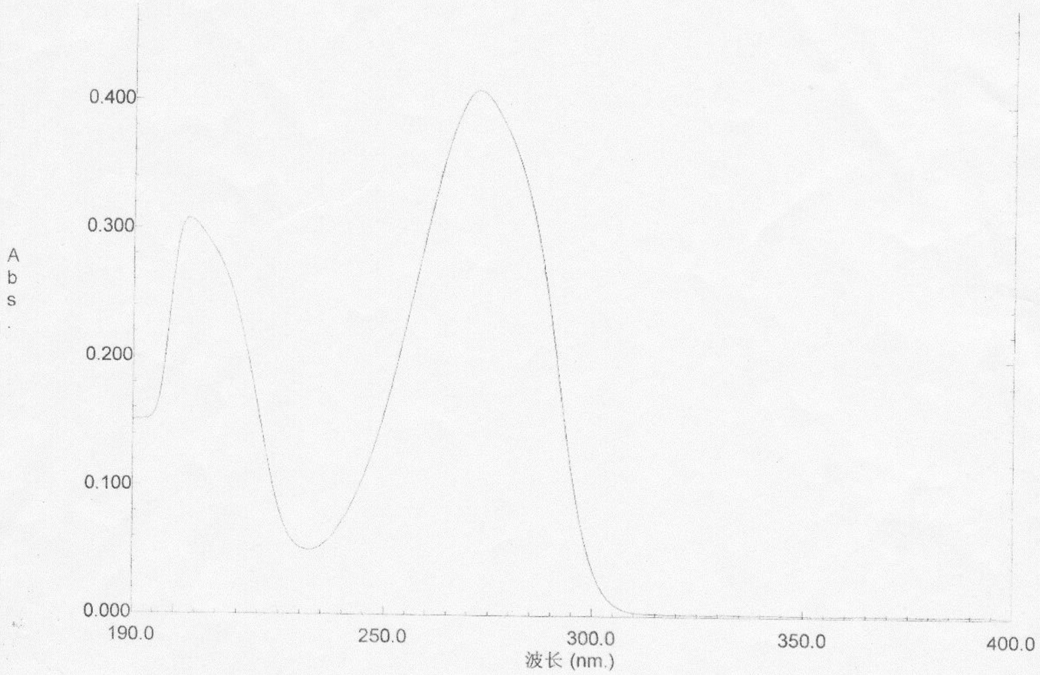

文件名: 15083100
样品名称：SH917C2-5

创建于: 09:45 15-08-31
数据: 原始

测量模式: Abs.
扫描速度: 中速
狭缝: 5.0
采样间隔: 0.2

否.	波长 (nm.)	Abs.
1	272.20	0.4091
2	203.20	0.3076

15083100

样品浓度：0.0037毫克/毫升
溶剂：甲醇

Fig. S79 IR spectrum of **8**

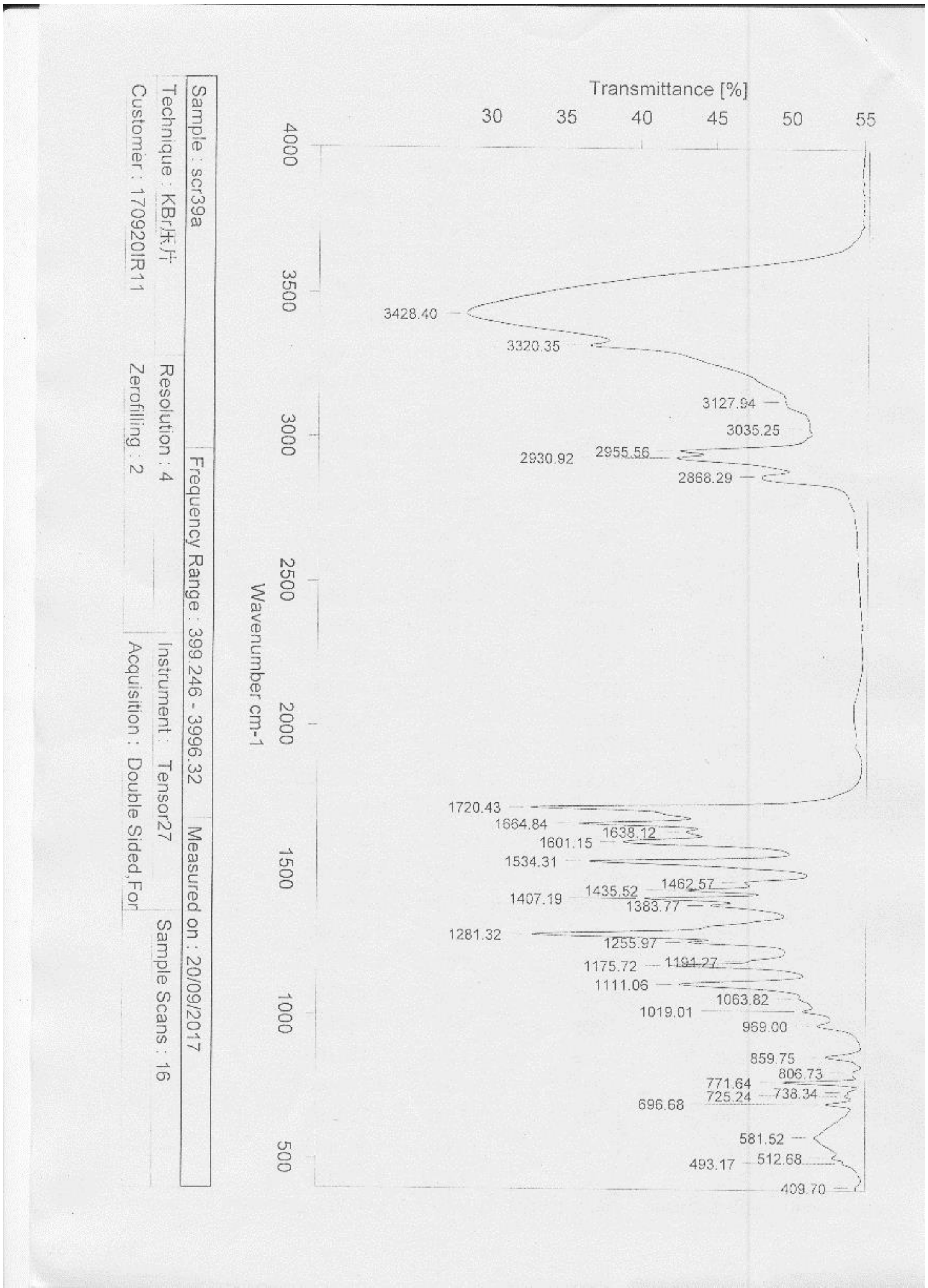

Supplement: Supplementary file 1 — Supplementary material 1 (PDF 11123 kb). Supplementary data associated with this article including 1D and 2D NMR, HRESIMS, UV, IR of 1–8 are available [file 13659_2018_158_MOESM1_ESM.pdf]
